# Association of urinary ketamine and APOA1 levels with bladder dysfunction in ketamine abusers revealed via proteomics and targeted metabolite analyses

**DOI:** 10.1038/s41598-021-89089-4

**Published:** 2021-05-05

**Authors:** Jo-Chuan Liu, Yi-Ting Chen, Ya-Ju Hsieh, Chia-Chun Wu, Ming-Chyi Huang, Yu-Chao Hsu, Chun-Te Wu, Chih-Ken Chen, Srinivas Dash, Jau-Song Yu

**Affiliations:** 1 Graduate Institute of Biomedical Sciences, College of Medicine, Chang Gung University, Taoyuan, Taiwan; 2 Molecular Medicine Research Center, Chang Gung University, Taoyuan, Taiwan; 3 Department of Biomedical Sciences, College of Medicine, Chang Gung University, Taoyuan, Taiwan; 4 Department of Addiction Sciences, Taipei City Psychiatric Center, Taipei City Hospital, Taipei, Taiwan; 5 Department of Psychiatry, School of Medicine, College of Medicine, Taipei Medical University, Taipei, Taiwan; 6 Department of Urology, Linkou Chang Gung Memorial Hospital, Taoyuan, Taiwan; 7 College of Medicine, Chang Gung University, Taoyuan, Taiwan; 8 Department of Urology, Chang Gung Memorial Hospital, Keelung, Taiwan; 9 Department of Psychiatry, Chang Gung Memorial Hospital, Keelung, Taiwan; 10 Liver Research Center, Linkou Chang Gung Memorial Hospital, Taoyuan, Taiwan; 11 Research Center for Food and Cosmetic Safety, College of Human Ecology, Chang Gung University of Science and Technology, Taoyuan, 33303 Taiwan

**Keywords:** Urogenital diseases, Diseases, Urology, Bladder

## Abstract

Chronic ketamine abuse is associated with bladder dysfunction and cystitis. However, the effects of ketamine abuse on the urinary proteome profile and the correlations among urinary proteins, urinary ketamine (and metabolites) and clinicopathological features of ketamine-induced bladder dysfunction remain to be established. Here, we recruited 56 ketamine abusers (KA) and 40 age-matched healthy controls (HC) and applied the iTRAQ-based proteomics approach to unravel quantitative changes in the urine proteome profile between the two groups. Many of the differentially regulated proteins are involved in the complement and coagulation cascades and/or fibrotic disease. Among them, a significant increase in APOA1 levels in KA relative to control samples (392.1 ± 59.9 ng/ml vs. 13.7 ± 32.6 ng/ml, *p* < 0.0001) was detected via ELISA. Moreover, urinary ketamine, norketamine and dehydronorketamine contents (measured via LC-SRM-MS) were found to be positively correlated with overactive bladder syndrome score (OABSS) and APOA1 levels with urinary RBC, WBC, OABSS and numeric pain rating scale in KA. Collectively, our results may aid in developing new molecular tool(s) for management of ketamine-induced bladder dysfunction. Moreover, information regarding the differentially regulated proteins in urine of KA provides valuable clues to establish the molecular mechanisms underlying ketamine-induced cystitis.

## Introduction

Ketamine hydrochloride, initially synthesized in 1962 by Calvin Stevens, a scientist at Parke-Davis Laboratories, is a noncompetitive antagonist of N-methyl-d-aspartic acid receptor used as a general anesthetic with short-term effect in both human and veterinary settings^1^. Ketamine undergoes N-demethylation by liver microsomal cytochrome P450 enzymes to generate the primary metabolite norketamine^2^. Dehydrogenation of norketamine generates dehydronorketamine, which is conjugated to glucuronic acid before excretion in urine. Levels of ketamine and its metabolites are detectable in urine up to 2 weeks after consumption using enzyme-linked immunosorbent assay (ELISA)^3^ or liquid chromatography-tandem mass spectrometry (LC–MS/MS)^4^.

In addition to its medical applications, ketamine is widely used as a recreational drug, especially in nightclubs and parties. Besides several negative effects (increased heart and respiratory rates, convulsions, temporary paralysis, nausea, vomiting, and hallucinations), use of ketamine can cause addictive effects, such as altered sensations, out-of-body experiences and a euphoric rush^5^. Since the first report of “ketamine-associated ulcerative cystitis” as a new clinical entity in 2007^6^, accumulating evidence has supported an association of chronic ketamine abuse with harmful physical effects, such as acute cardiac risk, ulcerative cystitis, lower urinary tract symptoms (LUTS), kidney dysfunction, intense abdominal pain and unexplained deaths^7^. Secondary and irreversible renal damage may occur in critical cases, leading to complete dependence on dialysis. Street ketamine abuse is not only a drug problem but also correlated with severe urological conditions that cause a significant burden to the healthcare system^8,9^. This highlights the critical need to clarify the mechanisms underlying development of ketamine-induced lower urinary tract dysfunction, which could provide useful clues for its clinical management.

Bladder dysfunction is one of the main characteristics of ketamine-induced LUTS, including irregular voiding frequency, decreased intercontraction intervals and bladder capacity, tissue fibrosis, injury to the urinary barrier and reduced urothelium areas of the bladder wall^10–12^. Serious bladder inflammation induces overactive bladder syndrome, irritative bladder symptoms and bladder pain. For evaluation of overactive bladder syndrome, scientists have developed a symptom assessment tool designated ‘overactive bladder symptom score’ (OABSS) that includes symptoms such as urgency, frequency (daytime and nighttime) and urinary incontinence^13^. It has been reported that overactive bladder syndrome caused by serious bladder inflammation is frequently observed in KA^14^. These observations support the correlation between OABSS and urinary ketamine and its metabolites.

Ketamine and its metabolites are excreted via urine. Direct toxic effects of these compounds on uroepithelial cells have been documented as one of the possible mechanisms underlying ketamine-induced bladder dysfunction^15,16^. Urine is in direct contact with uroepithelial cells and proteins released from damaged uroepithelial cells and/or bladder wall during long-term exposure to ketamine and its metabolites may be enriched in urine of KA. Therefore, non-invasive urine specimens of KA represent an ideal sample to identify useful indicators and/or the pathological mechanisms underlying ketamine-induced bladder dysfunction. Although a number of investigations have focused on the global changes in gene or protein expression levels in tissue specimens from bladders of mice subjected to long-term consumption of ketamine^17–19^ and KA^20^, to our knowledge, no studies have analyzed the proteome profile of urine samples from KA and relevant associations with ketamine-induced LUTS or urinary ketamine and its metabolites.

Chronic ketamine abuse can induce bladder dysfunction but the molecular mechanism still remains unclear. We supposed that direct toxic effects of ketamine and its metabolites may strongly affect uroepithelial cells or bladder wall, cause release of proteins into urine, and induce bladder dysfunction, including overactive bladder syndrome. The effects of ketamine abuse on the urinary proteome profile and the correlations among urinary proteins, urinary ketamine (and metabolites) and clinicopathological features of ketamine-induced bladder dysfunction need to be established. In the present investigation, we collected urine samples from KA and age-matched healthy controls along with clinical information, including laboratory data on urine/serum samples, OABSS and pain rating scales. LC–MS/MS was applied to measure the ketamine and metabolite contents in urine samples of KA and the urine proteome profile alterations in KA versus healthy controls investigated using the iTRAQ-based quantitative proteomics approach. Proteins displaying significantly altered levels were subjected to bioinformatics analysis for disrupted biological pathways in KA, and the urinary levels of two target proteins, APOA1 and SAA4, determined via ELISA in all enrolled subjects. Finally, the potential associations among urinary target protein levels, ketamine and metabolite contents and clinicopathological features of KA were evaluated.

## Results

### Study population and experimental design

**Figure 1:**
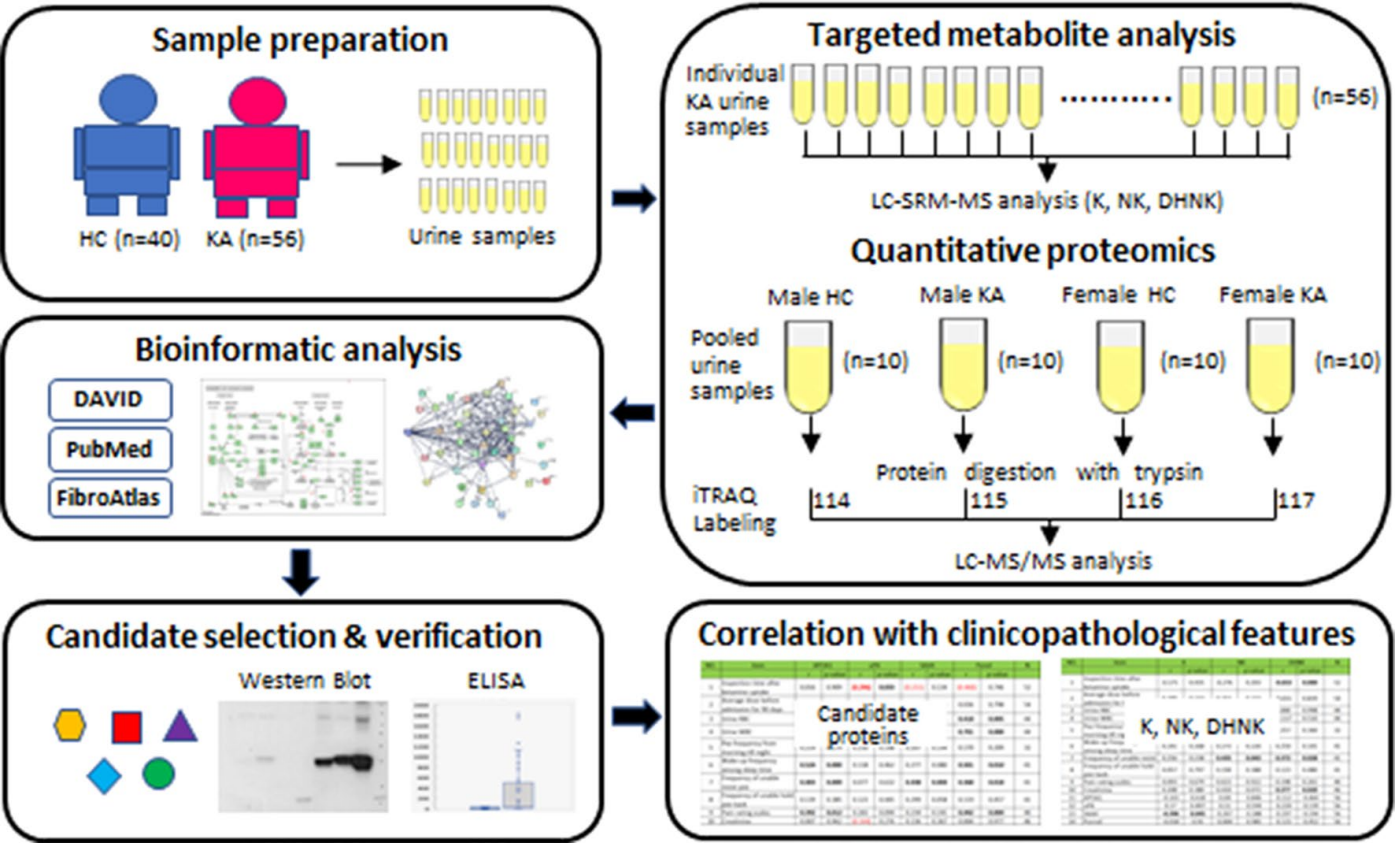
Workflow for exploring urine proteome alteration in KA and the association between urine protein levels, contents of ketamine and its metabolites and clinicopathlogical features of KA. Please refer to the text for details.

We recruited 56 KA (41 males and 15 females) and 40 age-matched healthy controls (25 males and 15 females) for study. Examination times after ketamine uptake for male and female KA were 6.0 ± 6.0 and 6.0 ± 4.8 days and average doses of ketamine uptake in the 90 days before admission were 4.0 ± 2.2 and 3.4 ± 2.6 g per day, respectively (Table 1). Urine samples were collected from all subjects for measurement of ketamine (and metabolite) contents as well as proteome profile analysis. A schematic presentation of the strategy used is shown in Fig. 1. We initially assessed the ketamine, norketamine and dehydronorketamine contents in individual KA urine samples via targeted metabolite analysis using LC-SRM-MS. Subsequently, pooled urine protein samples from 4 subgroups (male HC, male KA, female HC and female KA; 10 cases per group) were prepared for iTRAQ-based quantitative proteomics analysis to compare the urinary proteome profiles between HC and KA for both genders. Proteins showing differential expression patterns between HC and KA groups were selected for subsequent bioinformatics analysis to determine the biological network(s) potentially related to ketamine-induced bladder dysfunction. Several candidate proteins were further selected for verification using western blot and ELISA in pooled and individual urine samples. The correlations among urinary protein levels, contents of ketamine and its metabolites and clinicopathological features of KA were additionally evaluated.

**Table 1:** Demographic characteristics of enrolled subjects.

	Male		Female	
**Total enrolled subjects in this study**				
Group	HC	KA	HC	KA
Case no	25	41	15	15
Age (year)	31.4 ± 6.7	31.4 ± 6.7	31.6 ± 3.6	30.9 ± 5.1
Inspection time after ketamine uptake (day)	NA	6.0 ± 6.0	NA	6.0 ± - 4.8
Average dose before admission for 90 days (g/day)	NA	4.0 ± 2.2	NA	3.4 ± 2.6
**Enrolled subjects used for ITRAQ-based quantitative proteomics study**				
Group	HC	KA	HC	KA
Case no	10	10	10	10
Age (year)	31.9 ± 5.2	31.8 ± 13.7	31.6 ± 1 3.6	28.9 ± 4.5
Inspection time after ketamine uptake (day)	NA	6.0 ± 15.6	NA	6.8 ± 5.3
Average dose before admission for 90 days (g/day)	NA	4.9 ± 2.8	NA	3.8 ± 3.0

*HC* healthy controls, *KA* ketamine abusers, *NA* not applicable.

### Clinicopathological features of KA

Clinical data on the 56 KA detailed in Table S1 are summarized in Table 2. Different clinical data were available from 40 to 54 cases. Forty cases (77%, 40/52) visited the addiction center for ketamine detoxification 1 day or more after ketamine uptake and 46 cases (85%, 46/54) had consumed more than 2 g ketamine per day over the 3 months before admission. Laboratory data showed that 10 (23%, 10/44) and 17 (39%, 17/44) subjects contained urine RBC and WBC > 5/HPF, respectively, implicating inflammation in the lower urinary tract for more than a third of KA cases. Questionnaire-based survey data revealed that 21 (51%, 21/41) and 14 (35%, 14/40) cases had overactive bladder syndrome score (OABSS) > 6 and pain rating scale > 50 mm, respectively, suggesting that a significant proportion of the KA group developed ketamine-induced LUTS.

**Table 2:** Clinicopathological features of ketamine abusers enrolled in this study.

Features	Case no.^a^	Mean ± SD
**Inspection time after ketamine uptake (day)**	(n = 52)	
< 1	12	0.75 ± 0.45
2–7	24	3.88 ± 1.62
> 7	16	13.13 ± 4.65
**Average dose before admission for 90 days (g/day)**	(n = 54)	
< 1	8	0.89 ± 0.21
2–5	37	3.56 ± 1.46
> 5	9	7.44 ± 1.59
**Urine RBC (RBC/HPF)**	(n = 44)	
< 5	34	1.16 ± 0.67
> 5	10	142.54 ± 164.68
**Urine WBC (WBC/HPF)**	(n = 44)	
< 5	27	1.78 ± 1.30
> 5	17	94.15 ± 137.26
**Serum creatinine (mg/dL)**	(n = 46)	
< 1	41	0.75 ± 0.14
> 1	5	4.24 ± 6.22
**OABSS (overactive bladder syndrome score)**	(n = 41)	
0–5	20	2.45 ± 1.61
6–9	13	7.15 ± 1.14
10–15	8	11.63 ± 1.77
**Pain rating scale (mm)**^**b**^	(n = 40)	
0–50	26	11.19 ± 13.87
51–100	14	77.32 ± 13.85

^a^Data from 2 to 16 cases of KA are not available for each feature.

^b^A person rates his/her pain on a scale of 0 to 100 nm. Zero means “no pain”, and 100 means “the worst possible pain”.

### Urinary ketamine, norketamine and dehydronorketamine contents in KA

Ketamine and its metabolites can be detected in biofluids, such as urine and plasma. Using tetra-deuterated ketamine, norketamine and dehydronorketamine as internal standards, we developed a LC-SRM-MS method to estimate the contents of ketamine and its metabolites in KA urine samples (“Material and methods” section). Figure S1 depicts the response curves of ketamine, norketamine and dehydronorketamine in a urine matrix background. The limit of detection (LOD) of ketamine, norketamine and dehydronorketamine with LC-SRM-MS was determined as 7.6, 36 and 176.8 ng/ml, respectively. Levels of ketamine and its metabolites in urine specimens of each KA were determined in triplicate (Table S2). Ketamine, norketamine and dehydronorketamine were detected and quantified in 26, 17 and 29 KA subjects, respectively, with concentration ranges of 47–7121 (ketamine), 64–5461 (norketamine) and 202–12,953 (dehydronorketamine) ng/ml. Notably, levels of ketamine and its metabolites were highly correlated (ketamine vs. norketamine (r = 0.96, *p* < 0.001), ketamine vs. dehydronorketamine (r = 0.924, *p* < 0.001), norketamine vs. dehydronorketamine (r = 0.943, *p* < 0.001); Fig. S2). In general, higher levels of ketamine and metabolites were detected in urine samples from KA with higher ketamine uptake before hospital admission and shorter examination time after ketamine uptake.

### Quantitative proteome profiling of urine samples from KA and healthy controls

**Figure 2:**
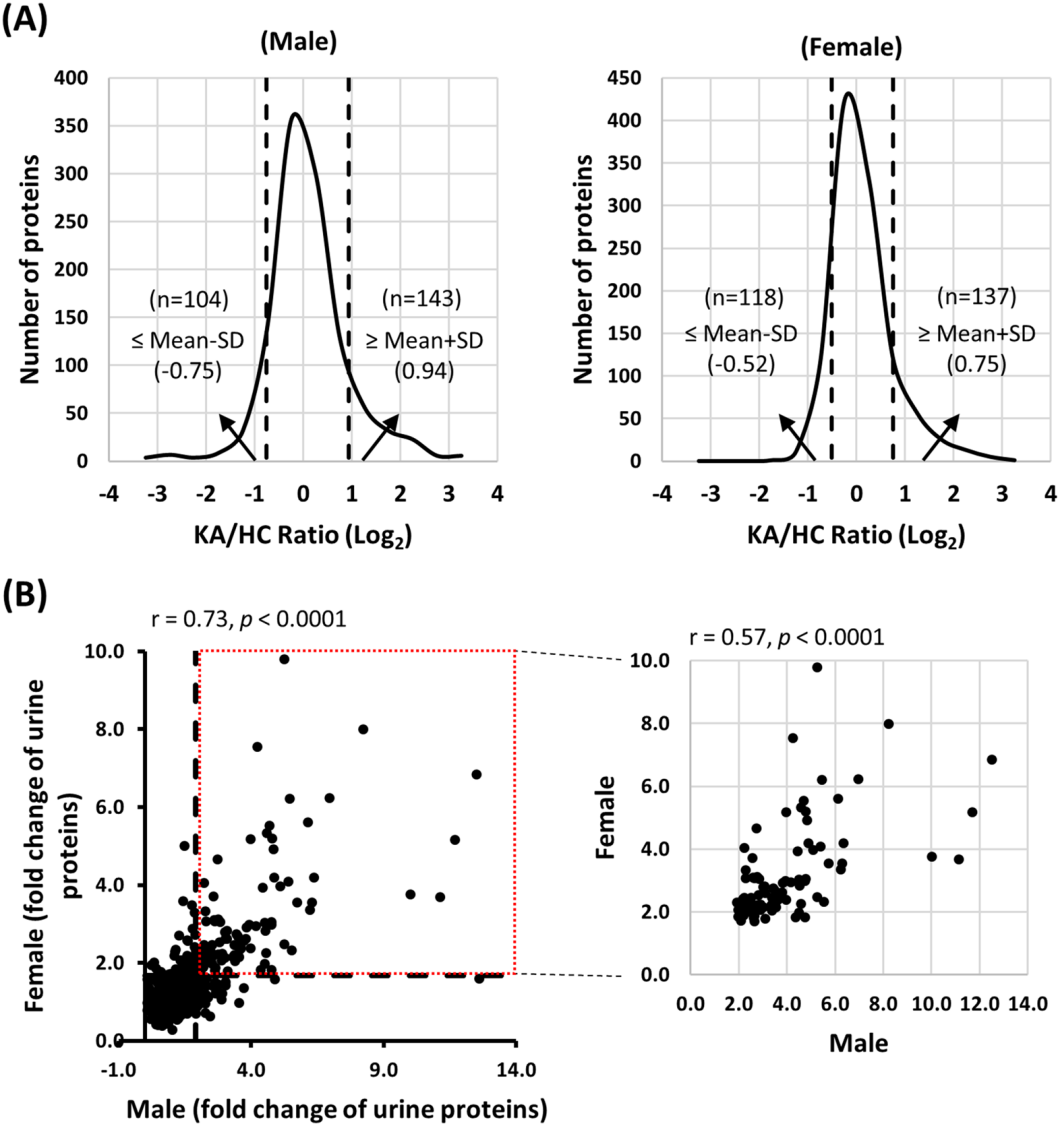
Quantitative comparison of urine proteome profiles between KA and HC for both genders. (**A**) Log_2_ ratio distribution of the 1113 quantified urine proteins between KA and HC of male (left panel) and female (right panel) groups. The dashed lines indicate the boundaries at mean ± SD. (**B**) Assessment of the correlation between fold changes of up- and downregulated proteins in both genders. Good correlation (r = 0.73, p < 0.0001) was observed for all the quantified proteins (left panel). Correlations of proteins showing > twofold changes in both genders were further analyzed (r = 0.57, p < 0.0001; right panel).

We applied the iTRAQ-based proteomics approach for quantitative comparison of the urinary proteome profiles for both genders of HC and KA groups using pooled urine protein samples from 4 subgroups (male HC, male KA, female HC and female KA; 10 cases for each group) as shown in Table 1. Prior to iTRAQ labeling, each urine sample was assessed via SDS-PAGE and silver staining to ensure protein quality. Starting from 36 μg, a total of 1113 urinary proteins were quantified. Among the quantified proteins, levels of 143 and 137 proteins were elevated (> mean + 1SD) and those of 104 and 118 proteins were reduced (< mean-1SD) in urine samples of male and female KA, respectively, with 93 elevated and 33 diminished proteins common to both genders (Table S3, Fig. 2A). A complete list of quantified proteins in urine samples of KA and healthy controls is presented in Table S4, and up- and downregulated proteins listed in Tables S5 and S6, respectively. The results showed that 1/10–1/5 urinary proteins are significantly altered in KA compared to healthy subjects, clearly indicating a profound effect of ketamine abuse on the urine protein profile. Assessment of the quantification data further revealed a good correlation between fold changes of up- and down-regulated proteins in both men and women (Fig. 2B), suggesting a similar effect of ketamine abuse on the urine protein profile in both genders. Determination of urinary proteins that are significantly altered in KA may be helpful in clarifying the mechanisms underlying ketamine induced cystitis frequently observed in cases of long-term ketamine abuse.

### Biological process network analysis of differentially regulated urinary proteins in KA

**Figure 3:**
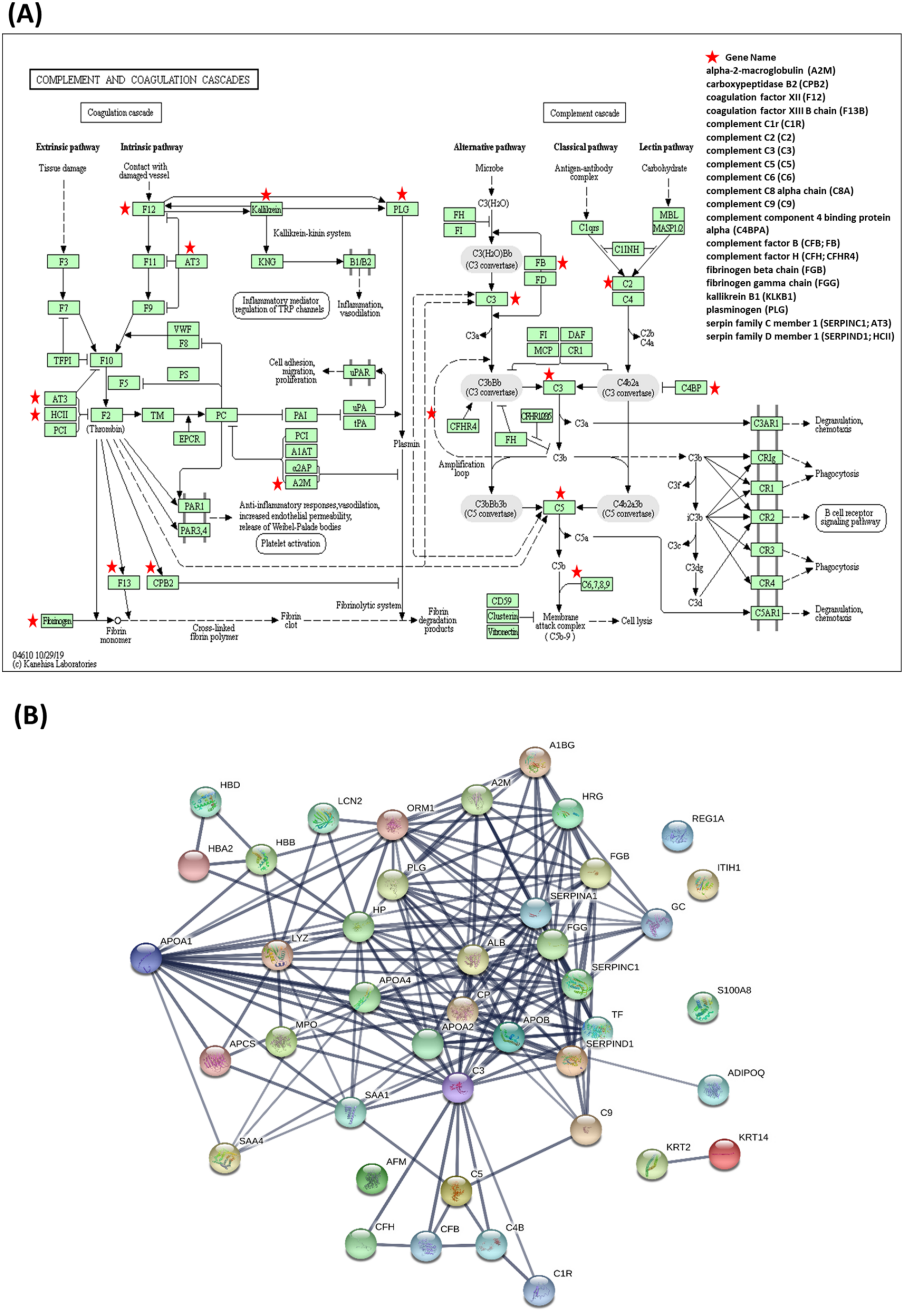
Biological process network analysis of differentially regulated urine proteins in KA. (**A**) KEGG pathway map for the complement and coagulation cascades identified as the top pathway enriched from a proportion of differentially expressed proteins (protein count = 20, denoted by asterisks). (**B**) Protein–protein interaction networks for 46 fibrosis-related proteins analyzed using STRING (PPI enrichment p-value < 10e−16, average local clustering coefficient of 0.583). Line thickness indicates the strength of data support.

To explore the biological processes potentially involved in the pathogenesis of ketamine cystitis, we applied DAVID, an integrated software suite for functional analysis of experimental data, to analyze the 126 differentially regulated urine proteins (93 elevated and 33 diminished) in both genders of the KA group. Gene ontology (GO) network analysis showed involvement of these differentially regulated proteins in networks of complement activation, proteolysis, fibrinolysis, innate immune response, platelet degranulation, and blood coagulation, among other functions (Table S7). Further KEGG pathway analysis confirmed ‘complement and coagulation cascades’ as the top pathway enriched from a proportion of differentially expressed proteins (protein count = 20, *p* value = 1.001E−25, FDR = 1.04E−22; Table S8), which involves interactions among coagulation, complement system and fibrinolysis (Fig. 3A).

Bladder fibrosis resulting from irreversible injury to the bladder is one of the major symptoms of ketamine cystitis in heavy ketamine users. We searched FibroAtlas, a database of fibrotic diseases and associated genes^21^, to examine for fibrosis-related proteins in differentially expressed urinary proteins of KA. More than one-third (46 of 126) of the proteins were annotated as fibrosis-related proteins in the FibroAtlas database (Table S9). Among the 46 proteins, 32 were found to be related to hepatic fibrosis, cystic fibrosis, pulmonary fibrosis, idiopathic pulmonary fibrosis, cardiac fibrosis, renal fibrosis and/or acute interstitial pneumonia. Notably, ten have been identified as fibrosis biomarkers, specifically, apolipoprotein A-I, alpha-2-macroglobulin, serotransferrin, vitamin D-binding protein, chitinase-3-like protein 1, protein S100-A9, matrilysin, hemopexin, retinol-binding protein 4 and osteopontin. Further analysis using the functional protein association network database (STRING) with a high confidence setting (0.7) showed tight/complex associations between fibrosis-related proteins (PPI enrichment *p*-value < 10e−16, average local clustering coefficient of 0.583; Fig. 3B).

Interestingly, several complement proteins, including complement C2, C3 and C5, have strong links to other fibrosis-related proteins (n = 16, 0.749 < score < 0.998), some of which (APOA1, GC, SPP1, TF) are known biomarkers in idiopathic pulmonary, hepatic and cardiac fibrosis. The complement system stimulates immune functions, including inflammation, phagocytosis and membrane attack, and a series of immune responses^22^. Additionally, APOA1 acts as a hub linking other fibrosis-related proteins in this network analysis (n = 16, 0.740 < score < 0.999; Fig. 3B), such as alpha-2-macroglobulin (A2M), apolipoprotein C-I (APOC1), fibrinogen gamma chain (FGG), haptoglobin (HP), hemopexin (HPX), and osteopontin (SPP1), most of which are activated in hepatic fibrosis. These results support an important role of APOA1 in ketamine-induced disorders.

### Verification of differentially regulated proteins in urine samples of healthy controls and KA

**Figure 4:**
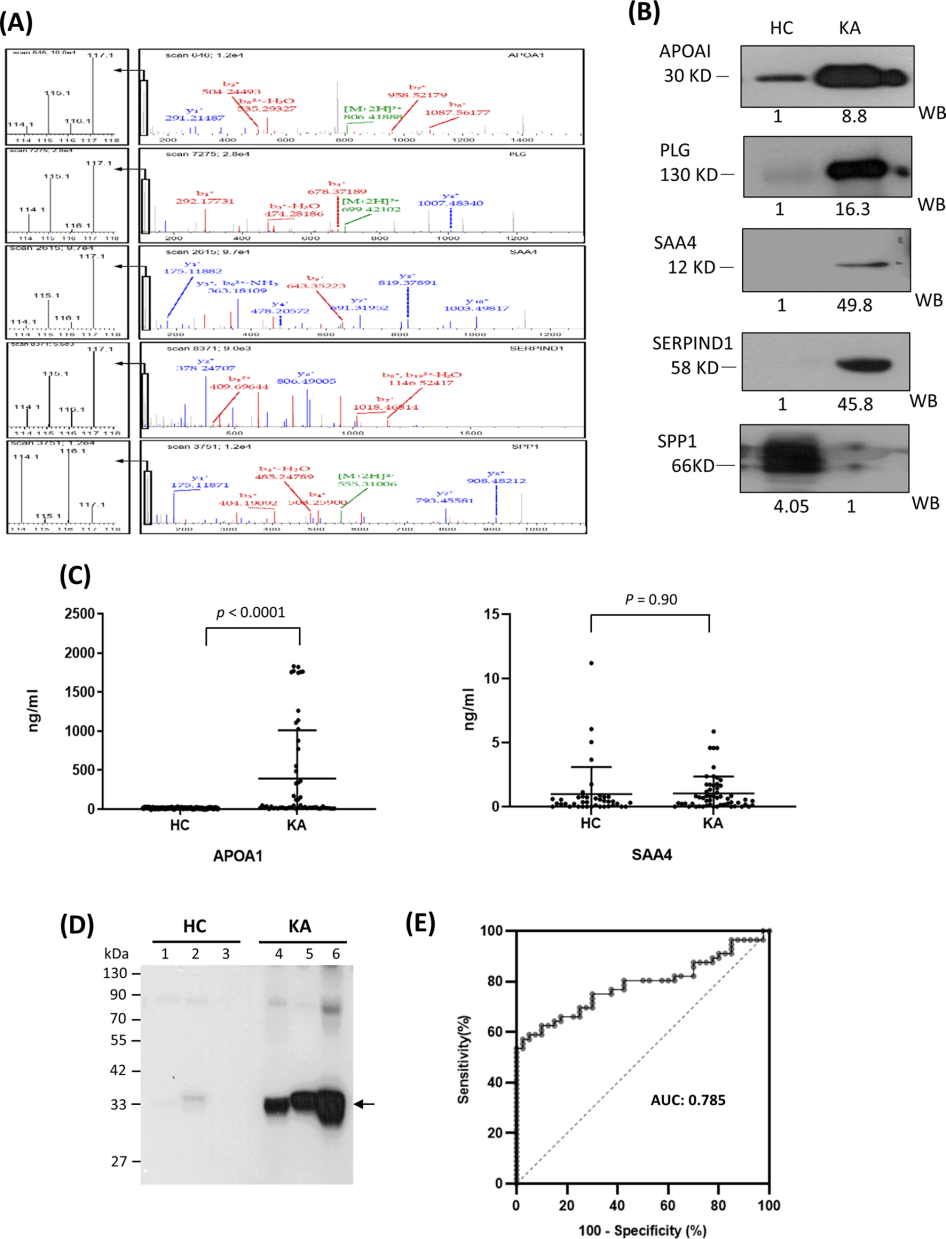
Validation of differentially regulated proteins in urine samples of HC and KA groups. (**A**) LC–MS/MS quantification of five selected proteins (APOA1, PLG, SAA4, SERPIND1 and SPP1) with significantly altered urinary levels between HC and KA. The low mass reporter ion region (used for quantification) in the right panel is enlarged in the left panel. (**B**) Western blot analysis of the five selected proteins (50 μg) in urine samples pooled respectively from HC and KA (three males and three females per group). Relative levels of each protein quantified from the Western blot are denoted below the respective image. Full images of the cropped blots are detailed in Fig. S3. (**C**) ELISA analysis of APOA1 and SAA4 levels in individual urine samples from 40 HC and 56 KA. (**D**) Western blot analysis of APOA1 in urine samples (50 μg protein) from three HC and three KA. The position of APOA1 is denoted with an arrow. (**E**) ROC curve analysis of APOA1 with utility in differentiating KA from HC.

According to iTRAQ results and relevance to ketamine cystitis, four upregulated (apolipoprotein A-I (APOA1), serum amyloid A-4 protein (SAA4), heparin cofactor 2 (SERPIND1) and plasminogen (PLG)) and one down-regulated (osteopontin (SPP1)) candidate proteins were selected for further verification in urine samples. Figure 4 depicts the results of MS analyses for quantification and identification of the five selected candidates (Fig. 4A) as well as western blot of urine samples originally used for iTRAQ experiments using specific antibodies against the five proteins (Fig. 4B). Our findings clearly showed consistency between MS-based and antibody-based quantification results, although the fold difference for target proteins was generally higher using western blot.

Next, we assessed levels of the five proteins in the 96 urine samples collected from 40 healthy controls and 56 KA via ELISA. However, upon testing of several commercially available ELISA kits, only two (for APOA1 and SAA4) could be confidently used for measuring levels in urine. Notably, levels of APOA1, but not SAA4, were significantly increased in KA relative to control samples (392.1 ± 59.9 ng/ml vs. 13.7 ± 32.6 ng/ml for APOA1, p < 0.0001; 1.0 ± 1.3 ng/ml vs. 0.99 ± 2.1 ng/ml for SAA4, p = 0.9) (Fig. 4C). Three representative cases of increased APOA1 levels in individual urine samples detected via western blot are shown in Fig. 4D. The AUC value of ROC curve analysis for APOA1 in discrimination of KA (n = 56) from controls (n = 40) was 0.785(Fig. 4E), supporting the utility of APOA1 as a potential urinary indicator of chronic ketamine abuse.

### Associations between urinary levels of ketamine and its metabolites and clinicopathological features

Data on associations between urinary ketamine and metabolite levels and clinicopathological features (5 items) are shown in Table 3. OABSS of the KA group were significantly associated with levels of ketamine (r = 0.338, *p* = 0.038), norketamine (r = 0.348, *p* = 0.030) and dehydronorketamine (r = 0.463, *p* = 0.003). Ketamine (r = 0.358, *p* = 0.030), but not norketamine and dehydronorketamine, showed significant association with the pain rating scale of KA. Urinary RBC and WBC counts and serum creatinine levels were not significantly associated with levels of ketamine and its metabolites.

**Table 3:** Associations between urinary contents of ketamine and metabolites and clinicopathological features of KA.

Item	Feature (case no.)	K		NK		DHNK	
		r	p-value	r	p-value	r	p-value
1	Urine RBC (n = 44)	0.209	0.207	0.083	0.610	0.177	0.257
2	Urine WBC (n = 44)	0.127	0.447	− 0.045	0.781	0.061	0.699
3	Serum creatinine (n = 46)	0.210	0.193	0.226	0.149	0.210	0.166
4	OABSS (n = 41)	0.338	0.038	0.348	0.030	0.463	0.003
5	Pain rating scale (n = 40)	0.358	0.030	0.255	0.122	0.231	0.158

*K* ketamine, *NK* norketamine, *DHNK* dehydronorketamine, *OABSS* overactive bladder syndrome score.

### Associations between urinary APOA1/SAA4 levels and clinicopathological features of KA

Urinary APOA1 was significantly associated with RBC (r = 0.463, *p* = 0.002), WBC (r = 0.721, *p* < 0.001), OABSS (r = 0.426, *p* = 0.006) and pain rating scale (r = 0.392, *p* = 0.012), but not serum creatinine, or urinary levels of ketamine and its metabolites (Table 4). Urinary SAA4 was significantly correlated with OABSS (r = 0.415, *p* = 0.007) but no other clinicopathological features or urinary levels of ketamine and its metabolites.

**Table 4:** Correlations among clinicopathological features, APOA1 and SAA4 urine levels in 56 KA.

Item	Feature (case no.)	APOA1		SAA4	
		r	p-value	r	p-value
1	Urine RBC (n = 44)	0.463	0.002	0.108	0.485
2	Urine WBC (n = 44)	0.721	0.000	0.090	0.562
3	Serum creatinine (n = 46)	0.007	0.962	0.136	0.367
4	OABSS (n = 41)	0.426	0.006	0.415	0.007
5	Pain rationg scale (n = 40)	0.392	0.012	0.279	0.082
6	[K] (n = 56)	0.202	0.16	0.101	0.486
7	[NK] (n = 56)	0.066	0.641	0.017	0.906
8	[DHNK] (n = 56)	0.196	0.151	0.159	0.247

## Discussion

Urine is an information-rich fluid containing numerous proteins produced or shed in the kidney and urogenital tract. Alterations of the human urinary proteome profile have been reported in response to disease or drug toxicity, particularly those affecting the kidney and urogenital tract^23^. Although the detrimental effect of ketamine abuse on the lower urinary tract has been well established for more than a decade, very little is known about the urinary proteome profile and its association with ketamine-induced LUTS in KA. To our knowledge, the present study is the first to explore this issue by simultaneously evaluating the levels of urine ketamine and its metabolites in KA, comparing the urinary proteome profiles between KA and control subjects, and assessing their relationships with the clinicopathological features of KA.

Using LC-SRM-MS, the concentration ranges of ketamine and its metabolites in urine specimens of KA collected in this study were determined as 47–7121 ng/ml (for ketamine, median 584 ng/ml), 64–5461 ng/ml (for norketamine, median 730 ng/ml) and 202–12,953 ng/ml (for dehydronorketamine, median 2212 ng/ml). Our data are consistent with the findings of Cheng et al^24^ on levels of ketamine and its metabolites in 22 urine specimens collected from suspected drug users in Taiwan in 2004 determined via gas chromatography–mass spectrometry (GC–MS). The group found that the concentration range of ketamine was the narrowest (20–7196 ng/ml) with the lowest median (332 ng/ml) while that of dehydronorketamine was the widest (36–17,629 ng/ml) with the highest median (891 ng/ml). The concentration range of NK was between 25 and 7685 ng/ml^24^. It is noted that only in half of the KA subjects that ketamine, norketamine and/or dehydronorketamine were detected (Table S2). The reasons for this observation can be manifold. First, inspection time after ketamine uptake varied significantly between KA subjects (0–24 days). Longer inspection time after ketamine uptake would allow metabolization of more ketamine and removal of more metabolized ketamine from the body via urinary excretion. Second, average dose of ketamine consumption for 90 days prior to admission differed significantly between KA subjects (0.5–10 g/day). Clearing of ketamine from the body would be faster in KA subjects with less dose of ketamine consumption. Third, personal variation of KA subjects in the ability to metabolize ketamine and remove metabolized ketamine from the body. All these factors can affect the detection/quantification of ketamine, norketamine and dehydronorketamine in the urine specimens of KA subjects enrolled in the present cross-sectional study.

Overactive bladder syndrome caused by serious bladder inflammation is frequently observed in KA. A recent survey on 106 KA cases in Taiwan reported that 84% ketamine users developed LUTS after two years, with an estimated OABSS of 5.25 ± 4.43^14^. Among the 41 KA with available OABSS data enrolled in this study, 21 (51%, 21/41) had OABSS > 6 (Table 2), indicating moderate to severe overactive bladder syndrome in more than half the KA participants. Based on both sets of available data (urinary levels of ketamine and metabolites and clinicopathological features of KA), we observed a positive association between overactive bladder syndrome (assessed via OABSS) and urinary levels of ketamine and its metabolites in KA (Table 3). While the reason for this correlation is currently unknown, recent findings on the ability of ketamine to induce inflammation and promote apoptosis in bladder tissues of a rat model and human SV-HUC-1 uroepithelial cells through regulating the NLRP3/TXNIP axis may provide an important clue^18^. It is possible that urinary ketamine directly activates the TXNIP/NLRP3 signaling pathway in uroepithelial cells to generate an inflammatory microenvironment in the affected bladder, which serves as one of the main factors causing overactive bladder syndrome^25^. However, some confounding factors need to be considered in the study of association between OABSS and urinary levels of ketamine and its metabolites in KA, including high variation of examination times after ketamine uptake, detoxification treatment, duration of ketamine use, and any other diseases which might influence the results potentially, such as urinary infection and sexually transmitted diseases.

Using pooled urine protein samples and the iTRAQ-based proteomics approach, we generated the first quantitative urine proteome map comparing KA and healthy control subjects of both genders. Consequently, a total of 126 differentially regulated proteins were identified (Table S3). Further bioinformatics analysis revealed 'complement and coagulation cascades’ as the top pathway enriched from these differentially regulated proteins (Table S7, Fig. 3A). Moreover, one-third (46 out of 126) of the differentially regulated proteins were annotated as fibrosis-related proteins in the FibroAtlas database, including several (A2M, FGG, CPB2, C3, F13B, CFB, C2 and C5) in the complement and coagulation cascades (Table S8). An association between the complement and coagulation cascades and specific fibrotic diseases has been previously reported^26,27^. For example, the extrinsic coagulation pathway together with C5a can promote fibrosis in bronchopulmonary dysplasia (a serious pulmonary fibrotic disorder) via the endothelin-1 signaling pathway^26^. Moreover, significant upregulation of proteins in the complement and coagulation cascades has been observed in colon and rectal tissues in a rat model of radiation-induced colorectal fibrosis^27^. These observations, together with our findings, raise the intriguing possibility that the complement and coagulation cascades are involved in the process of bladder fibrosis frequently observed in heavy ketamine users. Further experiments are essential to validate this hypothesis and the associated mechanisms.

Among the upregulated candidate proteins in urine samples, we successfully detected increased levels of APOA1 via ELISA in KA (n = 56) relative to healthy controls (n = 40) (Fig. 4C–E). This increase was significant, since all 40 healthy controls contained low urinary APOA1 levels (0–26.2 ng/ml) whereas more than half (30 out of 56) KA displayed markedly higher urinary APOA1 levels (27.2–1830.8 ng/ml), indicating good power of urinary APOA1 to discriminate KA from control subjects (AUC = 0.785). Notably, we observed significant association of urinary APOA1 levels with OABSS and pain rating scale in addition to urinary RBC/WBC levels (Table 4). To our knowledge, APOA1 represents the first urine protein component associated with ketamine-induced bladder dysfunction. Moreover, among the clinical parameters, urinary APOA1 showed the highest correlation with WBC level (r = 0.721, *p* < 0.001) (Table 4), an important quantitative indicator of inflammation in the kidney or lower urinary tract. This finding suggests that ketamine-induced inflammation in the kidney or lower urinary tract serves as the major factor underlying elevation of urinary APOA1.

The issue of whether the drastic increase in urinary APOA1 plays a pathophysiological role in the disease process of ketamine-induced bladder dysfunction remains unclear. APOA1, a major component of high-density lipoprotein (HDL) in plasma, mediates the reverse transport of cholesterol from peripheral cells to the liver for excretion^28,29^. In addition to its potential protective effects on the cardiovascular system and lowering of cardiovascular disease risk, accumulating evidence over the past decade supports the multifunctional nature of APOA1 with immunity, anti-inflammation, apoptosis, anti-clotting and anti-aggregatory effects^30^. Regarding anti-inflammation activity, APOA1 acts as a “negative” acute-phase protein that inhibits the production of interleukin-1beta and tumor necrosis factor-alpha by blocking contact-mediated activation of monocytes by T lymphocytes^31^. Interestingly, an idiopathic pulmonary fibrosis (IPF) animal model study showed that intranasal treatment with APOA1 protein reduced the bleomycin-induced increase in the number of inflammatory cells and collagen deposition in sham-treated mice in a dose-dependent manner, demonstrating anti-inflammatory and anti-fibrotic effects of APOA1 protein on experimental lung injury and fibrosis^32^. These previous findings raise another intriguing possibility that elevated urinary APOA1 modulates acute or chronic inflammation during ketamine-induced stress on bladder cells, which warrants further in-depth investigation.

One of the main limitations of this study is the small sample size used to verify the differentially regulated proteins identified via iTRAQ proteomics. The validity of our findings should be tested with larger collections of samples from different hospitals. Another limitation is the cross-sectional design of our study in which only one urine specimen was obtained for each KA. Multiple urine samples longitudinally collected from a set of KA subjects during the entire detoxification period should allow in-depth analysis of the correlations among urine ketamine and its metabolites, urinary proteins and clinical features for individual subjects and minimize the effects of personal variability.

Using the iTRAQ-based proteomics approach, we have demonstrated quantitative changes in the urine proteome profile in KA relative to healthy controls. Many of the differentially regulated proteins are involved in the complement and coagulation cascades and/or fibrotic disease. Among numerous up- and downregulated candidate proteins, a significant increase in APOA1 expression in urine samples of KA was detected via ELISA. This information, together with the urinary levels of ketamine, norketamine and dehydronorketamine (measured via LC-SRM-MS) and clinical data (assessed via laboratory tests and a questionnaire-based survey), supports positive associations of urinary ketamine and APOA1 with OABSS and pain rating scale, two of the main bladder dysfunction symptoms of KA. Identification of APOA1 as the first urinary protein component associated with ketamine-induced bladder dysfunction may aid in developing new molecular tool(s) for management of ketamine-associated LUTS. Moreover, information regarding the differentially regulated proteins in urine of KA provides valuable clues to establish the molecular mechanisms underlying ketamine-induced cystitis.

## Material and methods

### Study subjects and sample collection

All ketamine abusers (KA) and age-matched healthy controls (HC) included for study were over 20 years of age. All HC urine samples (n = 40) were collected at Chang Gung University, Taoyuan, Taiwan, and urine samples of KA (n = 56) admitted to an addiction center for ketamine detoxification obtained at Taipei City Psychiatric Center between 2014 and 2017. All urine samples were stored at − 80 °C. The study protocol was approved by the Medical Ethics and Human Clinical Trial Committee at Chang Gung Memorial Hospital, Taiwan, and Taipei City Hospital (Taipei, Taiwan). Experiments were conducted according to the principles of the Declaration of Helsinki and reviewed and approved by the Institutional Review Board (IRB) of Chang Gung Medical Foundation (Taoyuan, Taiwan; IRB no:106-0191C), and Taipei City Hospital (Taipei, Taiwan; IRB no: TCHIRB-1030408). Prior to sample collection, an IRB-approved informed consent form was signed by each participant. The inclusion criteria were as follows: (1) age between 18 and 60 years; (2) fulfilling DSM-IV-TR criteria for ketamine dependence as verified by two board-certified psychiatrists; (3) last ketamine use within 30 days (by self-report) prior to admission; (4) an ability to read Chinese and provide informed consent. The exclusion criteria were: (1) other substance use disorder (including abuse and dependence) in the past year except nicotine; (2) history of schizophrenia, bipolar disorder, or major depressive disorder, or having been treated with antipsychotics, mood stabilizers (including lithium, valproic acid, carbamazepine, and quetiapine), or antidepressants; (3) history of systemic medical illnesses such as hypertension, metabolic disorders (e.g., diabetes mellitus), or renal or liver diseases; (4) history of head injury, loss of consciousness, or neurological disorders; (5) inability or refusal to provide urine sample. Healthy controls were enrolled from the volunteers in Chang Gung University with inclusion criteria as follows: (1) age between 18 and 60 years; (2) no other substance use disorder (including abuse and dependence) in the past year, except nicotine; (3) no known systemic or neurological diseases such as hypertension, metabolic disorders (e.g., diabetes mellitus), or renal or liver diseases; (4) an ability to read Chinese and provide informed consent. All the participants were given a comprehensive description of the study and then recruited after giving written informed consent. The demographic characteristics of the 96 enrolled subjects are shown in Table 1 and clinical information on 56 KA (including laboratory data on urine/serum samples, OABSS and pain rating scales) in Table S1. For evaluation of overactive bladder syndrome, a total OABS score of ≤ 5 is defined as mild, 6–11 as moderate, and ≥ 12 as severe^13^. The first morning urine samples were collected in the presence of a protease inhibitor cocktail tablet (one tablet per 50 ml urine; Roche, Mannheim, Germany) and 1 mM sodium azide. Samples were centrifuged at 5000×*g* for 30 min at 4 °C within 5 h to remove cells and debris, and supernatant fractions stored at − 20 °C until further use.

### LC-SRM-MS analysis of ketamine and metabolites in urine samples

Ketamine and its metabolites (norketamine and dehydronorketamine) were quantified using the LC–MS/MS-based assay according to the method of Parkin et al*.*^33^, with slight modifications. Briefly, urine samples were spiked with an equal volume of a mixture of tetra-deuterated ketamine (ketamine-D4) (Cerilliant, Round Rock, TX, USA) and its derivatives (norketamine-D4 and dehydronorketamine-D4) (Toronto Research Chemicals, Ontario, Canada) at 1 pmol/μl in 0.1% formic acid in methanol. Urine samples were centrifuged at 10,000×*g* for 10 min at 4 °C, and the collected supernatant fractions subjected to LC–MS/MS analysis in the selected reaction monitoring (SRM) mode using Waters ACQUITY UPLC (ultra-performance liquid chromatography; Hertfordshire, UK) coupled with a HCT ultra mass spectrometer (Bruker Daltonik GmbH, Bremen, Germany). The mobile system was as follows: A, 0.1% formic acid in water and B, acetonitrile with 0.1% formic acid. The flow rate was 60 μl/min and a linear gradient was set as follows: 0 min, 8% B; 2 min, 8% B; 12 min, 18% B; 14 min, 20% B; 15 min, 95% B; 18 min, 95% B. MS data were acquired in the SRM mode: isolation with a 10 amu peak width the first time and 1 amu the second time, followed by smart fragmentation ramping from 0.3 to 2 V. The peak areas of fragments were detected and integrated using the software package DataAnalysis 4.2. (Bruker Corporation, MA, USA).

Quantification was performed by calculating the peak area ratio of the product ion for ketamine (m/z 220.1) to its tetra-deuterated analog (m/z 224.1) ketamine-d4, norketamine (m/z 207.1) to norketamine-d4 (m/z 211.1), and likewise, dehydronorketamine (m/z 205.0) to dehydronorketamine-d4 (m/z 209.0). Each urine sample was analyzed in triplicate, and levels of ketamine, norketamine and dehydronorketamine calculated as mean ± SD (ng/ml). Assay linearity for ketamine, norketamine and dehydronorketamine was determined by constructing response curves from urine to which no or nine different concentrations (fivefold serial dilutions ranging from 50 to 0.128 fmol/μl) of ketamine, norketamine and dehydronorketamine were added as calibrants (0.5 pmol/μl tetra-deuterated analog). A linear regression model with a weighting function of log-transformed light to heavy ratio (X axis) and peak area (Y axis) was applied and linearity achieved with R^2^ ≥ 0.995. The limit of detection (LOD) was defined as the lowest concentration with a mean signal-to-noise ratio of ≥ 3 based on the peak height of all three ion transitions and limit of quantification (LOQ) as signal-to-noise ratio ≥ 10 of the quantifying ion and at least 3 for the two qualifying transitions.

### Concentration and desalting of urine samples

Urine proteins were concentrated using a 10 kDa centrifugal filter (Millipore, Carrigtwohill, Ireland) as described previously^34^. Briefly, urine samples (12.5 ml) were centrifuged at 5000×*g* for 30 min at 4 °C in a filter tube, followed by the addition of 12.5 ml of 20% acetonitrile/H_2_O and re-centrifugation. This process was repeated once using pure water for desalting. Samples were subjected to an additional desalting step using 4 ml H_2_O to avoid possible interference from metabolites in the labeling reaction for iTRAQ labeling. After estimation of protein quantity with a Pierce BCA protein assay kit (Thermo Scientific, MA, USA), each concentrated/desalted urine sample was lyophilized and stored at − 80 °C for subsequent processing.

### Tryptic digestion of urinary proteins and iTRAQ reagent labeling

We applied pooled urine samples from different groups (male HC, male KA, female HC and female KA; 10 cases per group) for iTRAQ-based quantitative proteomics analysis to minimize individual variations and enhance signals. The 10 cases were randomly selected from each subgroup with age between 26 and 36 to generate gender- and age-matched subgroups. Equal amounts of protein (10 μg) from individual samples were pooled into a subgroup. Pooled urinary protein (100 μg) from each subgroup was reduced, cysteine-blocked and digested with trypsin at 37 °C for 16 h as described previously^34^. Digested peptides were labeled with iTRAQ reagent (Applied Biosystems, Foster City, CA, USA) according to the manufacturer’s protocol. Peptide mixtures of male HC, male KA, female HC and female KA were labeled with iTRAQ tags 114, 115, 116 and 117, respectively. After incubation at room temperature for 1 h, peptide mixtures were pooled and desalted via SPE (solid phase extraction) of Oasis HLB (30 μm) cartridges (Waters, Massachusetts, USA) and vacuum dried.

### Two-dimensional LC–MS/MS analysis

Dried iTRAQ labeling peptides (30 μg) were reconstituted in 50 μl HPLC mobile phase A (30% acetonitrile/0.1% formic acid) and loaded onto a homemade strong cation exchange chromatography (SCX) column (Luna SCX 5 μm, 0.5 × 255 mm, Phenomenex, Torrance, CA, USA) at flow rate of 5 µl/min for 30 min. Peptides were eluted with 0–100% HPLC mobile phase B (0.5 M ammonium chloride/30% acetonitrile/0.1% formic acid) and separated into 66 fractions using online 2D-HPLC (Dionex Ultimate 3000; Thermo Fisher, San Jose, CA, USA). Each SCX fraction was further diluted in-line prior to trap of reverse-phase column (Zorbax 300SB-C18 5 μm, 0.3 × 5 mm; Agilent Technologies, Wilmington, DE, USA) and diluted peptides were resolved on an analytical C18 column (Synergi Hydro-RP 2.5 µm, 0.075 × 200 mm with a 15 μm tip; Phenomenex, Torrance, CA, USA). A linear gradient of fractionation was applied as follows: 3–28% HPLC mobile phase C (99.9% acetonitrile/0.1% formic acid) for 37 min, 28–50% mobile phase C for 12 min, 50–95% mobile phase C for 2 min, 95% mobile phase C for 5 min, and 3% mobile phase C for 9 min), with a flow rate of 0.3 μl/min. LC apparatus was coupled with a two-dimensional linear ion trap mass spectrometer (LTQ-Orbitrap ELITE; Thermo Fisher, San Jose, CA, USA) controlled by Xcalibur 2.2 software (Thermo Fisher, San Jose, CA, USA). Full-scan MS was performed in the Orbitrap over a range of 400 to 2000 Da and a resolution of 60,000 at m/z 400. Internal calibration was performed using the ion signal of [Si (CH3)2O]6H + at m/z 445.120025, 462.146574, and 536.165365 as lock masses. The twelve data-dependent MS/MS scan events of six collision-induced dissociation (CID) mode and six high-energy collision induced dissociation (HCD) mode were followed with one MS scan for the six most abundant precursor ions in a preview MS scan.

### Sequence database search and quantitative data analysis

Data analysis was performed using Proteome Discoverer software (version 1.4, Thermo Fisher Scientific) involving the reporter ion quantifier node for iTRAQ quantification. MS/MS spectra were searched against the Swiss-Prot human sequence database (released on 20,150,429, selected for *Homo sapiens*, 20,199 entries) using the Mascot search engine (Matrix Science, London, UK; version 2.2.6). For protein identification, the precursor mass tolerance was set to 10 ppm and fragment ion mass tolerance to 0.5 Da for CID mode by ion analysis and 0.05 Da for HCD mode by orbitap analysis, with allowance for one missed cleavage from tryptic digestion. Fixed modification was set to methylthiolation at cysteine (+ 45.99 Da) and variable modification to acetylation at protein N-terminus (+ 42.01 Da), oxidation at methionine (+ 15.99 Da), pyroglutamate conversion at N-terminal glutamine (− 17.03 Da) and iTRAQ4-plex labeling at lysine and peptide N-terminus (+ 144.10 Da). Data were filtered based on medium confidence of peptide identification to ensure an overall false discovery rate below 0.01. Proteins with a single peptide hit were removed, and quantitative data exported as Excel files from Proteome Discoverer and manually normalized, such that log2 of iTRAQ ratio displayed a median value of zero for all peptides in a single given protein. Proteins with log2 ratios above the mean of all log2 ratios plus one standard deviation (SD) of all log2 ratios were considered upregulated, and those with log2 ratios below the mean minus one SD were classified as downregulated.

### Bioinformatics and network analysis

The Database for Annotation, Visualization, and Integrated Discovery (DAVID, v6.7, http://david.abcc.ncifcrf.gov/) and Kyoto Encyclopedia of Genes and Genomes (KEGG) database resource were used to test for the enrichment of biological processes^35,36^. Proprietorial gene ontology (GO) biological process with a false-discovery rate (FDR) < 0.05 was considered significant in enrichment analysis. Fibrosis-related proteins were retrieved from FibroAtlas, a database for the exploration of fibrotic diseases and their genes^21^. Protein–protein interaction networks were analyzed using STRING: functional protein association networks (version 11.0)^37^.

### Western blot analysis

Western blot was analysis performed as described previously^34^. Briefly, urine samples (containing 50 μg protein) were resolved on 10% SDS gels and transferred to PVDF membrane that were probed using primary antibodies against the candidate proteins of interest, including anti-APOA1 (Proteintech, Rosemont, USA), anti-SERPIND1 (Proteintech), anti-osteopontin (Proteintech), anti-plasminogen (Thermo Fisher, Rockford, USA) and anti-SAA4 (Abnova, Taipei, Taiwan). The relative signal intensity of each target protein detected in the blots was quantified using a computing densitometer (Molecular Dynamics, Sunnyvale, CA).

### Quantification of APOA1 and SAA4 by ELISA

APOA1 and SAA4 levels in urine samples were measured using sandwich ELISA kits (R&D Systems, MN, USA) and Elabscience (Hubei, China), respectively, according the manufacturers’ protocols. Briefly, 100 μl of each urine sample (2- and fourfold diluted for HC and KU subjects, respectively) was added to the wells of antibody-coated microplates and incubated at room temperature for 2 h, followed by five washes with wash buffer. Biotinylated antibody was added to the wells and incubated for 1 h. After a further five washes with wash buffer, 200 μl substrate solution was added to each well and incubated for 30 min at room temperature. Finally, 50 μl stop solution was added to each well and the color intensity measured at different times at a wavelength of 450 nm using a Spectra Max M5 microplate reader (Molecular Devices, Sunnyvale, CA).

### Statistical analysis

The correlations between ELISA data and clinicopathological features of patients were analyzed with the non-parametric Mann–Whitney U test using the statistical package SPSS 15.0 (SPSS Inc., Chicago, IL, USA). Receiver operator characteristic (ROC) curve and area under the curve (AUC) analyses were utilized to detect the optimal cutoff point that produced the greatest total accuracy for clinical classification. Spearman correlation analysis was applied to assess the correlations between urine contents of ketamine (and metabolites), ELISA data and clinicopathological features of ketamine users. Results are presented as coefficient of correlation (r) and the corresponding *p*-value. Statistical assessments were two-tailed and significance set at *p* < 0.05.

### Ethics approval and consent to participate

The study protocol was approved by the Medical Ethics and Human Clinical Trial Committee at Chang Gung Memorial Hospital, Taiwan and Taipei City Hospital, Taipei, Taiwan. This study was conducted according to the principles expressed in the Declaration of Helsinki and was reviewed and approved by the Institutional Review Board (IRB) of Chang Gung Medical Foundation, Taoyuan, Taiwan (IRB no:106-0191C) and Taipei City Hospital, Taipei, Taiwan (IRB no: TCHIRB-1030408). All subjects gave their written informed consent.

## Supplementary Information

Supplementary Information.

## Data Availability

The datasets generated and/or analyzed in the current study are not publicly available due to patient privacy, however they are available from the corresponding author on reasonable request.

## Abbreviations

APOA1: Apolipoprotein A1
AUC: Area under the ROC curve
GO: Gene ontology
HPF: High power field
iTRAQ: Isobaric tag for relative and absolute quantitation
HC: Healthy control
KA: Ketamine abuser
LC–MS/MS: Liquid chromatography tandem mass spectrometry
RBC: Red blood cell
ROC: Receiver operating characteristic curve
SAA4: Serum amyloid A-4 protein
SRM: Selected reaction monitoring
WBC: White blood cell
K: Ketamine
NK: Norketamine
DHNK: Dehydronorketamine
