# Supplementary material for: Association of urinary ketamine and APOA1 levels with bladder dysfunction in ketamine abusers revealed via proteomics and targeted metabolite analyses
Source: Sci Rep. 2021 May 5;11:9583. doi: 10.1038/s41598-021-89089-4 (PMC8099891; doi:10.1038/s41598-021-89089-4)
Supplement: Supplementary file 1 — Supplementary Information. [file 41598_2021_89089_MOESM1_ESM.pdf]

Supplementary information

Association of urinary ketamine and APOA1 levels with bladder dysfunction in ketamine abusers revealed via proteomics and targeted metabolite analyses

Jo-Chuan Liu [a], *Yi-Ting Chen* [a,b,c], *Ya-Ju Hsieh* [b], *Chia-Chun Wu*[b], *Ming-Chyi Huang* [d,e], *Yu-Chao Hsu* [f,g], *Chun-Te Wu* [g,h], *Chih-Ken Chen* [g,i], *Srinivas Dash* [a], *and Jau-Song Yu* [a,b,j,k*]

[a] Graduate Institute of Biomedical Sciences, College of Medicine, Chang Gung University, Taoyuan, Taiwan
[b] Molecular Medicine Research Center, Chang Gung University, Taoyuan, Taiwan
[c] Department of Biomedical Sciences, College of Medicine, Chang Gung University, Taoyuan, Taiwan.
[d] Department of Addiction Sciences, Taipei City Psychiatric Center, Taipei City Hospital, Taipei, Taiwan
[e] Department of Psychiatry, School of Medicine, College of Medicine, Taiwan Medical University, Taipei, Taiwan
[f] Department of Urology, Linkou Chang Gung Memorial Hospital, Taoyuan, Taiwan.
[g] College of Medicine, Chang Gung University, Taoyuan, Taiwan.
[h] Department of Urology, Chang Gung Memorial Hospital, Keelung, Taiwan.
[i] Department of Psychiatry, Chang Gung Memorial Hospital, Keelung, Taiwan
[j] Liver Research Center, Linkou Chang Gung Memorial Hospital, Taoyuan, Taiwan
[k] Research Center for Food and Cosmetic Safety, College of Human Ecology, Chang Gung University of Science and Technology, Taoyuan 33303, Taiwan

*Correspondence: yusong@mail.cgu.edu.tw; Tel.: +886-3-2118800 (ext. 5171); Fax: +886-3-2118891

Table S1. Clinical information on ketamine abusers enrolled in this study

Case No.	Sample name	Age	Sex	Inspection time after ketamine uptake (day)	Average dose before admission for 90 days (g/day)	Laboratory test results			Overactive Bladder Symptom Scores (OABSS)[a]	Pain rating scales (mm)[b]
						Urine RBC/ml /HPF (normal arrange : <5 /HPF)	Urine WBC/ml /HPF (normal arrange : <5/HPF)	Serum creatinine (mg/dL) (normal arrange : 0.5 to 1.5 mg/dL)		
1	NSC13-001	28	Female	10	6	1	4	NA	NA	NA
2	NSC13-002	38	Male	12	0.6	1	1	1	NA	NA
3	NSC13-007	25	Female	12	4	1	37.5	2.7	NA	NA
4	NSC13-009	35	Male	8	5	3.5	115	0.7	NA	NA
5	NSC13-011	23	Female	5	6	1	300	0.6	5	88
6	NSC13-013	36	Male	14	10	1	3.5	0.7	NA	NA
7	NSC13-014	43	Male	3	5	4	4	0.9	NA	NA
8	NSC13-015	30	Male	6	7.5	1	1	0.8	NA	NA
9	NSC13-017	24	Male	5	5	1	1	0.9	NA	NA
10	NSC13-022	35	Female	5	5	1	1	0.5	1	93
11	NSC13-023	40	Female	6	5	1	3.5	0.6	NA	NA
12	NSC13-025	38	Male	6	5	200	115	0.6	NA	NA
13	NSC13-026	31	Male	6	5	1	7.5	0.9	1	0
14	NSC13-029	27	Male	14	5	1	1	0.9	2	0
15	NSC13-030	26	Female	3	10	1	7.5	0.5	NA	NA
16	NSC13-034	34	Male	8	2	1	1	0.9	NA	NA
17	NSC13-036	34	Male	24	1	NA	NA	0.7	6	0
18	NSC13-040	30	Male	1	3	1	2	0.87	12	67
19	NSC13-042	24	Male	1	5	200	3.5	0.6	8	79
20	NSC13-044	18	Male	8	3	115	37.5	0.7	5	4
21	NSC13-056	30	Female	17	2	6	183	1.01	13	90.5
22	NSC13-059	34	Male	4	3	1	1	0.8	2	2
23	NSC13-061	37	Female	NA(<30)	1	1	115	0.7	8	79
24	NSC13-062	38	Male	1	5	1	1	0.8	1	28
25	NSC13-069	32	Male	3	6	1	1	1	6	53
26	NSC13-071	31	Male	4	5	NA	NA	0.8	3	0
27	NSC13-074	43	Male	14	5	1	1	0.6	12	42
28	NSC13-078	20	Male	4	4	1	1	0.6	0	0
29	NSC13-081	28	Male	0	6.5	1	5	1.11	NA	NA
30	NSC13-084	30	Female	7	2	108	84	0.76	7	65
31	NSC13-085	31	Female	0	2	NA	NA	NA	6	0
32	NSC13-086	24	Male	NA(<30)	NA	NA	NA	NA	3	0
33	NSC13-088	34	Male	1	2	1	115	NA	8	24
34	NSC13-090	38	Male	NA(<30)	2.5	6	18	0.81	10	71
35	NSC13-093	33	Male	2	7.5	21	6	15.3	10	NA
36	NSC13-094	30	Male	2	5	NA	NA	0.8	2	0
37	NSC13-097	37	Female	10	1.2	1	3.5	0.5	6	30
38	NSC13-105	31	Male	2	5	1	1	0.9	1	20
39	NSC13-107	50	Male	1	1.5	1	1	1	11	4
40	NSC13-108	41	Male	5	2	NA	NA	NA	NA	NA
41	NSC13-110	29	Female	2	1	1	7.5	0.6	3	70
42	NSC13-114	25	Male	2	5	1	1	0.8	5	11
43	NSC13-117	22	Male	1	5	1	1	0.6	7	20
44	NSC13-118	32	Male	13	2	500	17.5	0.8	9	40
45	NSC13-120	34	Male	1	7.5	1	1	0.8	15	98
46	NSC13-122	36	Male	3	5	93	500	0.72	10	75
47	NSC13-124	29	Male	2	1	1	1	0.8	6	35
48	NSC13-126	25	Female	4	1	1	1	0.6	0	4
49	NSC13-133	32	Male	1	1	1	1	0.7	3	0
50	NSC13-134	30	Male	9	1.5	NA	NA	NA	4	9
51	NSC13-138	26	Male	NA(<30)	NA	5.34	13.3	1.07	9	60
52	NSC13-140	29	Male	0	2	NA	NA	NA	NA	NA
53	NSC13-142	33	Female	1	2	NA	NA	NA	4	8
54	NSC13-145	34	Female	2	3	NA	NA	0.8	7	94
55	NSC13-151	23	Male	16	0.5	NA	NA	NA	1	0
56	NSC13-160	22	Male	21	2	NA	NA	NA	3	10

a, Homma Y, et al. Urology 2006; 68:318-323.

b, a tool to measure pain level, with a total legth of 100 mm (0 mm < pain level < 100 mm)

NA, Not applicable

Table S2. LC-SRM-MS analysis of levels of ketamine (K), norketamine (NK) and dehydronorketamine (DHNK) in urine samples of katamine users

Case No.	K (mean ± SD; ng/ml)	NK (mean ± SD; ng/ml)	DHNK (mean ± SD; ng/ml)	Inspection time after ketamine uptake (day)	Average dose before admission for 90 days (g/day)
1	<7.6	<36	<176.8	10	6
2	<7.6	<36	<176.8	12	0.6
3	<7.6	<36	<176.8	12	4
4	<7.6	<36	<176.8	8	5
5	<7.6	<36	<176.8	5	6
6	<7.6	<36	<176.8	14	10
7	<7.6	<36	<176.8	3	5
8	<7.6	<36	<176.8	6	7.5
9	<7.6	<36	<176.8	5	5
10	<7.6	<36	<176.8	5	5
11	<7.6	<36	<176.8	6	5
12	<7.6	<36	<176.8	6	5
13	<7.6	<36	<176.8	6	5
14	<7.6	<36	<176.8	14	5
15	<7.6	<36	345.6±14.7	3	10
16	<7.6	<36	<176.8	8	2
17	<7.6	<36	<176.8	24	1
18	1250.8±169.9	298.9±29.5	8316.4±1384.8	1	3
19	584.0±13.5	356.3±28.2	1832.6±254.4	1	5
20	<7.6	<36	<176.8	8	3
21	<7.6	<36	<176.8	17	2
22	<7.6	<36	<176.8	4	3
23	47.7±7.5	64.4±3.7	1260.4±192.3	NA	1
24	89.9±9.5	145.0±18.9	1380.9±221.1	1	5
25	191.3±13.8	113.0±2.5	700.2±26.9	3	6
26	<7.6	<36	<176.8	4	5
27	<7.6	<36	674.4±3.0	14	5
28	<7.6	<36	<176.8	4	4
29	1664.1±52.2	2383.7±192.6	3938.8±305.2	0	6.5
30	874.5±96.5	409.3±59.0	4165.3±477.9	7	2
31	5589.8±422.7	2220.1±217.0	4136.3±558.5	0	2
32	169.8±24.4	<36	799.1±52.9	NA	NA
33	7121.8±254.7	5461.2±148.0	9495.5±745.1	1	2
34	678.5±13.4	1203.5±53.5	12953.2±686.0	NA	2.5
35	454.7±11.3	260.4±15.2	8153.8±173.9	2	7.5
36	<7.6	<36	<176.8	2	5
37	5174.7±850.1	1530.6±269.0	4064.6±442.6	10	1.2
38	508.5±64.8	<36	265.3±25.2	2	5
39	212.8±36.7	<36	2906.5±574.1	1	1.5
40	2260.9±417.7	458.0±53.4	2212.2±235.2	5	2
41	183.4±12.0	<36	1290.1±67.5	2	1
42	98.2±18.2	<36	977.5±39.9	2	5
43	<7.6	<36	<176.8	1	5
44	68.6±6.8	<36	689.5±37.5	13	2
45	1452.7±35.2	1573.3±138.5	6971.0±550.4	1	7.5
46	1438.3±108.0	904.5±116.1	2297.9±369.3	3	5
47	109.4±11.4	<36	1595.1±86.4	2	1
48	<7.6	<36	<176.8	4	1
49	502.5±54.0	<36	2207.8±75.5	1	1
50	<7.6	<36	202.4±10.6	9	1.5
51	<7.6	<36	<176.8	NA	NA
52	2667.2±320.0	1261.7±123.1	3017.8±6.3	0	2
53	4092.5±805.0	703.2±125.0	3794.3±492.3	1	2
54	1427.0±99.8	<36	2230.0±146.7	2	3
55	<7.6	<36	<176.8	16	0.5
56	<7.6	<36	<176.8	21	2

NA, not applicable

LOD (ng/ml): K (7.6 ng/ml); NK (36.0 ng/ml); DHNK (176.8 ng/ml)

LOQ (ng/ml): K (37.9 ng/ml); NK (178.5 ng/ml); DHNK (176.8 ng/ml)

Table S3. Protein identification and quantification results from urine samples of ketamine abusers and healthy controls via iTRAQ.

Classification	Definition	Protein number		
		KA vs. HC (male)	KA vs. HC (female)	KA vs. HC (male & female)
Quantified proteins (QP)		1106	1108	1104
(I) KA[a] > HC[b]	Ratio[c] ≧ mean+1SD (male 1.915; female 1.682)	143	137	93
(II) KA < HC	Ratio ≦ mean-1SD (male 0.594; female 0.699)	104	118	33
(III) Non-significant change	mean-1SD < Ratio < mean+1SD	859	853	978

[a]KA, Ketamine abusers. [b]HC, Healthy controls. [c]Ratio, 115/114 (for male) or 117/116 (for female) ratio.

Table S4. List of proteins quantified in urine samples of ketamine abusers and healthy controls

(KA, ketamine user; HC, health control; med, median of exp for normalization; median of male KA/HC=0.989; median of female KA/HC =1.015; nor, normalization with median of exp.)

Accession	Protein name	Gene name	Coverage	Unique Peptides	Peptides	PSMs	AAs	MW [kDa]	calc. pI	Male KA / HC	Female KA / HC	Male KA/HC (nor.)	Female KA/HC (nor.)
Q8WZ42	Titin	TTN	0.19	5	5	8	34350	3813.7	6.35	1.012	0.285	1.022	0.281
Q71RC9	Small integral membrane protein 5	SMIM5	24.68	2	2	4	77	8.5	7.96	0.644	0.396	0.651	0.390
Q16849	Receptor-type tyrosine-protein phosphatase-like N	PTPRN	3.68	3	3	18	979	105.8	7.11	0.491	0.418	0.496	0.411
P23471	Receptor-type tyrosine-protein phosphatase zeta	PTPRZ1	0.99	2	2	7	2315	254.4	4.88	0.518	0.429	0.524	0.422
P31431	Syndecan-4	SDC4	18.18	3	3	12	198	21.6	4.50	0.496	0.432	0.501	0.426
Q16363	Laminin subunit alpha-4	LAMA4	2.69	4	4	17	1823	202.4	6.28	0.415	0.435	0.419	0.428
Q86Y38	Xylosyltransferase 1	XYLT1	1.98	2	2	9	959	107.5	9.22	0.744	0.450	0.752	0.443
Q86X29	Lipolysis-stimulated lipoprotein receptor	LSR	3.08	2	2	2	649	71.4	7.97	0.862	0.451	0.871	0.444
P80370	Protein delta homolog 1	DLK1	4.44	2	2	22	383	41.3	5.67	0.578	0.457	0.584	0.450
Q16832	Discoidin domain-containing receptor 2	DDR2	2.81	2	2	2	855	96.7	5.36	0.525	0.486	0.531	0.479
O95865	N(G),N(G)-dimethylarginine dimethylaminohydrolase 2	DDAH2	37.19	7	8	13	285	29.6	6.01	0.640	0.490	0.647	0.483
Q96GW7	Brevican core protein	BCAN	2.52	2	2	12	911	99.1	4.64	0.472	0.498	0.477	0.490
Q9BRT3	Migration and invasion enhancer 1	MIEN1	15.65	2	2	3	115	12.4	4.37	0.598	0.511	0.605	0.504
P05060	Secretogranin-1	CHGB	8.57	4	4	53	677	78.2	5.07	0.574	0.528	0.580	0.520
P25940	Collagen alpha-3(V) chain	COL5A3	6.25	6	6	10	1745	172.0	6.87	0.457	0.538	0.462	0.530
P22891	Vitamin K-dependent protein Z	PROZ	29.00	10	10	36	400	44.7	5.97	0.542	0.541	0.547	0.533
Q9NNX6	CD209 antigen	CD209	8.66	3	3	3	404	45.7	5.47	0.819	0.543	0.828	0.535
P39059	Collagen alpha-1(XV) chain	COL15A1	7.71	7	7	38	1388	141.6	5.00	0.465	0.543	0.470	0.535
Q8TDY8	Immunoglobulin superfamily DCC subclass member 4	IGDCC4	1.60	2	2	2	1250	134.1	6.20	0.588	0.549	0.594	0.541
Q6FHJ7	Secreted frizzled-related protein 4	SFRP4	8.09	3	3	11	346	39.8	8.82	0.928	0.552	0.938	0.544
Q9Y279	V-set and immunoglobulin domain-containing protein 4	VSIG4	7.02	2	2	3	399	44.0	6.35	1.190	0.558	1.203	0.550
A6NL88	Protein shisa-7	SHISA7	4.65	2	2	2	538	56.2	10.02	0.214	0.571	0.216	0.563
P35613	Basigin	BSG	5.45	2	2	2	385	42.2	5.66	0.716	0.575	0.724	0.566
Q8WWV6	High affinity immunoglobulin alpha and immunoglobulin mu Fc receptor	FCAMR	4.89	3	3	5	532	57.1	9.23	0.276	0.576	0.279	0.567
O14798	Tumor necrosis factor receptor superfamily member 10C	TNFRSF10C	7.34	2	2	2	259	27.4	4.82	0.572	0.577	0.578	0.568
P51693	Amyloid-like protein 1	APLP1	3.23	2	2	2	650	72.1	5.80	0.880	0.582	0.890	0.573
Q14982	Opioid-binding protein/cell adhesion molecule	OPCML	30.43	6	8	19	345	38.0	6.87	0.751	0.584	0.759	0.575
P13727	Bone marrow proteoglycan	PRG2	10.81	2	2	2	222	25.2	6.76	1.255	0.585	1.268	0.576
Q96FE7	Phosphoinositide-3-kinase-interacting protein 1	PIK3IP1	15.59	3	3	226	263	28.2	5.01	0.504	0.587	0.510	0.578
Q86TY3	Armadillo-like helical domain-containing protein 4	ARMH4	2.45	2	2	6	774	84.1	4.34	0.458	0.588	0.463	0.579
Q96RW7	Hemicentin-1	HMCN1	1.99	7	7	26	5635	613.0	6.49	0.912	0.591	0.922	0.582
Q9ULI3	Protein HEG homolog 1	HEG1	7.60	8	8	21	1381	147.4	6.18	0.445	0.593	0.449	0.584
O15240	Neurosecretory protein VGF	VGF	13.98	7	7	12	615	67.2	4.78	0.703	0.598	0.710	0.589
Q9P121	Neurotrimin	NTM	16.57	3	5	19	344	37.9	7.81	0.804	0.600	0.813	0.591
Q8N3J6	Cell adhesion molecule 2	CADM2	9.43	4	4	9	435	47.5	5.33	0.741	0.601	0.749	0.592
P01210	Proenkephalin-A	PENK	9.36	2	2	7	267	30.8	5.53	0.456	0.601	0.460	0.592
P05026	Sodium/potassium-transporting ATPase subunit beta-1	ATP1B1	8.25	2	2	3	303	35.0	8.53	1.179	0.604	1.191	0.595
P15151	Poliovirus receptor	PVR	9.83	4	4	8	417	45.3	6.52	0.496	0.606	0.501	0.596
P21695	Glycerol-3-phosphate dehydrogenase [NAD(+)], cytoplasmic	GPD1	23.50	5	5	7	349	37.5	6.18	0.831	0.606	0.840	0.597
P10451	Osteopontin	SPP1	49.68	15	15	350	314	35.4	4.58	0.527	0.609	0.533	0.600
P09210	Glutathione S-transferase A2	GSTA2	33.78	7	7	19	222	25.6	8.59	0.694	0.609	0.702	0.600
P21810	Biglycan	BGN	15.76	5	5	8	368	41.6	7.52	0.652	0.609	0.659	0.600
Q93088	Betaine--homocysteine S-methyltransferase 1	BHMT	22.41	6	8	34	406	45.0	7.03	1.025	0.612	1.035	0.603
Q96DA0	Zymogen granule protein 16 homolog B	ZG16B	38.46	7	7	27	208	22.7	7.39	0.692	0.612	0.699	0.603
Q8WU39	Marginal zone B- and B1-cell-specific protein	MZB1	17.99	2	2	2	189	20.7	5.57	0.905	0.615	0.914	0.605
P09564	T-cell antigen CD7	CD7	12.08	2	2	5	240	25.4	7.27	0.643	0.616	0.650	0.607
P55287	Cadherin-11	CDH11	13.07	9	9	23	796	87.9	4.91	0.911	0.616	0.921	0.607
P41217	OX-2 membrane glycoprotein	CD200	17.63	3	3	5	278	31.2	8.51	0.590	0.621	0.596	0.612
Q9H159	Cadherin-19	CDH19	2.46	2	2	4	772	86.9	4.73	0.735	0.622	0.742	0.612
P09619	Platelet-derived growth factor receptor beta	PDGFRB	8.32	7	7	21	1106	123.9	4.98	0.749	0.622	0.757	0.613
P40197	Platelet glycoprotein V	GP5	10.71	4	4	6	560	60.9	9.63	0.777	0.622	0.785	0.613
Q16769	Glutaminyl-peptide cyclotransferase	QPCT	54.85	11	11	157	361	40.9	6.61	0.592	0.625	0.598	0.615
Q13445	Transmembrane emp24 domain-containing protein 1	TMED1	13.22	2	2	2	227	25.2	4.48	0.529	0.625	0.534	0.616
P31997	Carcinoembryonic antigen-related cell adhesion molecule 8	CEACAM8	8.88	2	3	4	349	38.1	7.39	0.738	0.628	0.745	0.618
P05937	Calbindin	CALB1	46.36	10	10	29	261	30.0	4.83	0.852	0.628	0.861	0.619
P16070	CD44 antigen	CD44	8.49	5	5	231	742	81.5	5.33	0.695	0.630	0.702	0.620
O94856	Neurofascin	NFASC	1.71	2	2	2	1347	149.9	6.65	0.797	0.630	0.805	0.621
Q6UXB8	Peptidase inhibitor 16	PI16	16.20	7	7	31	463	49.4	5.39	1.177	0.633	1.190	0.624
P36639	7,8-dihydro-8-oxoguanine triphosphatase	NUDT1	19.80	3	3	7	197	22.5	5.27	0.573	0.635	0.579	0.625
Q6UXB4	C-type lectin domain family 4 member G	CLEC4G	21.50	4	4	10	293	32.5	6.61	1.011	0.639	1.022	0.629
Q9NU53	Glycoprotein integral membrane protein 1	GINM1	11.52	4	4	7	330	36.8	4.91	0.747	0.641	0.755	0.631
P21802	Fibroblast growth factor receptor 2	FGFR2	6.21	4	4	10	821	92.0	5.88	0.387	0.643	0.392	0.633
P19013	Keratin, type II cytoskeletal 4	KRT4	12.17	6	6	8	534	57.2	6.61	2.419	0.644	2.445	0.634
Q08334	Interleukin-10 receptor subunit beta	IL10RB	7.38	2	2	3	325	37.0	5.17	0.635	0.646	0.642	0.637
Q96J84	Kin of IRRE-like protein 1	KIRREL1	17.57	9	9	18	757	83.5	5.73	0.964	0.649	0.974	0.639
P07148	Fatty acid-binding protein, liver	FABP1	33.86	4	4	11	127	14.2	7.18	0.588	0.649	0.594	0.640
A6NI73	Leukocyte immunoglobulin-like receptor subfamily A member 5	LILRA5	25.42	5	5	13	299	32.7	6.99	0.853	0.650	0.862	0.640
Q6UXD5	Seizure 6-like protein 2	SEZ6L2	3.08	3	3	6	910	97.5	4.89	0.539	0.654	0.544	0.644
P11362	Fibroblast growth factor receptor 1	FGFR1	5.60	4	4	7	822	91.8	6.21	0.787	0.654	0.795	0.644
P05062	Fructose-bisphosphate aldolase B	ALDOB	33.79	11	12	47	364	39.4	7.87	1.122	0.656	1.134	0.646
P56537	Eukaryotic translation initiation factor 6	EIF6	36.33	5	5	9	245	26.6	4.68	0.308	0.656	0.312	0.646
Q07954	Prolow-density lipoprotein receptor-related protein 1	LRP1	1.52	6	6	11	4544	504.3	5.39	0.885	0.656	0.894	0.646
O43493	Trans-Golgi network integral membrane protein 2	TGOLN2	15.79	5	5	18	437	45.9	5.63	0.810	0.658	0.819	0.648
Q96DR8	Mucin-like protein 1	MUCL1	8.89	2	2	5	90	9.0	4.64	0.545	0.660	0.550	0.651
P51884	Lumican	LUM	30.77	8	8	32	338	38.4	6.61	1.067	0.661	1.079	0.651
O00592	Podocalyxin	PODXL	7.17	4	4	11	558	58.6	5.49	0.609	0.662	0.615	0.652
Q96JQ0	Protocadherin-16	DCHS1	3.31	7	7	10	3298	346.0	4.94	0.534	0.662	0.539	0.652
Q496F6	CMRF35-like molecule 2	CD300E	14.63	3	3	5	205	22.9	8.00	0.665	0.664	0.672	0.654
P45877	Peptidyl-prolyl cis-trans isomerase C	PPIC	13.21	3	3	7	212	22.7	8.40	0.752	0.667	0.760	0.657
P13611	Versican core protein	VCAN	2.71	8	8	18	3396	372.6	4.51	0.569	0.670	0.575	0.660
P06734	Low affinity immunoglobulin epsilon Fc receptor	FCER2	15.58	4	4	5	321	36.4	5.57	1.336	0.671	1.350	0.661
Q15746	Myosin light chain kinase, smooth muscle	MYLK	1.25	2	2	7	1914	210.6	6.15	0.596	0.673	0.602	0.663
Q6GTX8	Leukocyte-associated immunoglobulin-like receptor 1	LAIR1	14.98	4	4	27	287	31.4	5.63	0.713	0.673	0.721	0.663

P02462	Collagen alpha-1(IV) chain	COL4A1	2.34	3	3	5	1669	160.5	8.28	0.721	0.675	0.729	0.665
Q9UGM3	Deleted in malignant brain tumors 1 protein	DMBT1	4.68	3	3	4	2413	260.6	5.44	0.966	0.676	0.976	0.666
Q8TAB3	Protocadherin-19	PCDH19	3.48	3	3	7	1148	126.2	5.36	0.645	0.676	0.652	0.666
P01589	Interleukin-2 receptor subunit alpha	IL2RA	7.35	2	2	3	272	30.8	6.52	0.948	0.676	0.958	0.666
P05556	Integrin beta-1	ITGB1	3.01	2	2	4	798	88.4	5.39	1.305	0.677	1.319	0.667
P22304	Iduronate 2-sulfatase	IDS	8.00	3	3	6	550	61.8	5.45	0.570	0.677	0.576	0.667
Q12864	Cadherin-17	CDH17	4.57	3	3	5	832	92.2	5.14	0.826	0.680	0.835	0.670
Q96S96	Phosphatidylethanolamine-binding protein 4	PEBP4	47.14	7	7	14	227	25.7	6.54	0.831	0.680	0.840	0.670
P49747	Cartilage oligomeric matrix protein	COMP	14.66	7	7	22	757	82.8	4.60	0.832	0.681	0.841	0.670
Q9BY67	Cell adhesion molecule 1	CADM1	28.73	8	8	46	442	48.5	5.07	0.697	0.682	0.705	0.671
P11279	Lysosome-associated membrane glycoprotein 1	LAMP1	11.51	5	5	16	417	44.9	8.75	1.026	0.684	1.036	0.673
Q9BRK3	Matrix remodeling-associated protein 8	MXRA8	16.97	6	6	21	442	49.1	7.23	0.833	0.685	0.842	0.675
P19256	Lymphocyte function-associated antigen 3	CD58	8.00	3	3	6	250	28.1	6.76	0.891	0.686	0.900	0.676
O95460	Matrilin-4	MATN4	7.07	3	3	5	622	68.4	6.10	0.267	0.686	0.269	0.676
Q6UXE8	Butyrophilin-like protein 3	BTNL3	4.08	2	2	2	466	52.2	6.24	0.723	0.687	0.731	0.676
P07195	L-lactate dehydrogenase B chain	LDHB	45.81	13	14	37	334	36.6	6.05	0.669	0.688	0.676	0.678
P55017	Solute carrier family 12 member 3	SLC12A3	11.07	10	10	20	1021	113.1	7.88	0.919	0.691	0.929	0.680
Q04756	Hepatocyte growth factor activator	HGFAC	6.41	4	4	8	655	70.6	7.24	0.884	0.692	0.893	0.681
Q9H6X2	Anthrax toxin receptor 1	ANTXR1	8.69	3	3	13	564	62.7	7.61	1.033	0.693	1.044	0.682
Q14894	Ketimine reductase mu-crystallin	CRYM	17.20	4	4	6	314	33.8	5.14	0.690	0.694	0.697	0.683
P01034	Cystatin-C	CST3	47.95	6	6	63	146	15.8	8.75	0.564	0.694	0.570	0.684
P78380	Oxidized low-density lipoprotein receptor 1	OLR1	25.64	7	7	25	273	30.9	7.28	0.988	0.694	0.999	0.684
P25189	Myelin protein P0	MPZ	7.26	2	2	2	248	27.5	9.54	1.028	0.695	1.039	0.685
P30086	Phosphatidylethanolamine-binding protein 1	PEBP1	74.87	9	9	43	187	21.0	7.53	0.624	0.697	0.631	0.687
P08123	Collagen alpha-2(I) chain	COL1A2	6.44	7	7	19	1366	129.2	8.95	0.898	0.698	0.907	0.688
P16112	Aggrecan core protein	ACAN	3.75	7	7	11	2530	261.2	4.13	0.603	0.699	0.610	0.688
Q14019	Coactosin-like protein	COTL1	34.51	6	6	14	142	15.9	5.67	0.690	0.699	0.697	0.689
Q8WZ75	Roundabout homolog 4	ROBO4	17.28	11	11	37	1007	107.4	6.64	0.715	0.700	0.722	0.689
Q8TDQ0	Hepatitis A virus cellular receptor 2	HAVCR2	13.29	4	4	21	301	33.4	5.72	0.706	0.701	0.714	0.690
P09958	Furin	FURIN	4.66	2	2	2	794	86.6	6.47	0.472	0.701	0.477	0.691
Q96C23	Aldose 1-epimerase	GALM	16.37	4	4	6	342	37.7	6.65	1.571	0.703	1.588	0.692
P36957	Dihydrolipoyllysine-residue succinyltransferase component of 2 oxoglutarate dehydrogenase complex, mitochondria	DLST	7.06	3	3	13	453	48.7	8.95	0.781	0.703	0.790	0.693
Q9NY15	Stabilin-1	STAB1	1.01	2	2	2	2570	275.3	6.49	0.737	0.705	0.745	0.695
P49189	4-trimethylaminobutyraldehyde dehydrogenase	ALDH9A1	9.51	5	5	10	494	53.8	5.87	0.985	0.707	0.996	0.696
P02538	Keratin, type II cytoskeletal 6A	KRT6A	27.48	8	19	44	564	60.0	8.00	1.900	0.708	1.920	0.698
Q13332	Receptor-type tyrosine-protein phosphatase S	PTPRS	5.65	8	8	15	1948	216.9	6.46	0.756	0.710	0.764	0.699
Q01974	Tyrosine-protein kinase transmembrane receptor ROR2	ROR2	3.29	2	3	11	943	104.7	6.55	0.722	0.712	0.730	0.701
Q9HBB8	Cadherin-related family member 5	CDHR5	8.28	5	5	7	845	88.2	4.93	0.809	0.713	0.818	0.703
P23468	Receptor-type tyrosine-protein phosphatase delta	PTPRD	1.41	2	2	4	1912	214.6	6.57	1.325	0.714	1.339	0.703
Q7Z5L0	Vitelline membrane outer layer protein 1 homolog	VMO1	44.55	5	5	49	202	21.5	5.07	0.626	0.714	0.633	0.703
P30039	Phenazine biosynthesis-like domain-containing protein	PBLD	30.21	6	6	11	288	31.8	6.52	1.104	0.714	1.116	0.703
P14384	Carboxypeptidase M	CPM	28.44	12	12	54	443	50.5	7.36	0.701	0.714	0.708	0.704
P13598	Intercellular adhesion molecule 2	ICAM2	11.64	3	3	17	275	30.6	7.43	0.664	0.716	0.671	0.705
P24855	Deoxyribonuclease-1	DNASE1	47.16	7	7	129	282	31.4	4.91	0.841	0.716	0.850	0.705
O00533	Neural cell adhesion molecule L1-like protein	CHL1	16.64	17	17	32	1208	135.0	5.76	0.778	0.717	0.786	0.706
O95998	Interleukin-18-binding protein	IL18BP	22.16	4	4	36	194	21.1	7.39	0.737	0.720	0.745	0.709
Q15223	Nectin-1	NECTIN1	2.13	2	2	6	517	57.1	6.10	0.816	0.721	0.825	0.710
Q8WVV5	Butyrophilin subfamily 2 member A2	BTN2A2	8.41	6	6	35	523	59.0	6.01	0.523	0.723	0.528	0.712
Q13477	Mucosal addressin cell adhesion molecule 1	MADCAM1	15.97	3	3	8	382	40.1	5.12	1.199	0.723	1.212	0.712
Q96A22	Uncharacterized protein C11orf52	C11orf52	23.58	2	2	4	123	13.9	9.41	1.346	0.724	1.361	0.713
O60939	Sodium channel subunit beta-2	SCN2B	7.91	2	2	2	215	24.3	6.39	0.754	0.726	0.762	0.715
Q9BX67	Junctional adhesion molecule C	JAM3	13.87	4	4	9	310	35.0	7.59	0.983	0.726	0.993	0.715
Q9H1U4	Multiple epidermal growth factor-like domains protein 9	MEGF9	2.82	2	2	2	602	62.9	5.67	0.931	0.727	0.940	0.716
P35443	Thrombospondin-4	THBS4	7.08	5	5	9	961	105.8	4.68	0.804	0.728	0.813	0.717
Q7Z5N4	Protein sidekick-1	SDK1	2.85	4	4	8	2213	242.0	6.39	0.734	0.728	0.742	0.717
P01258	Calcitonin	CALCA	23.40	3	3	5	141	15.5	6.16	0.868	0.728	0.877	0.717
Q13508	Ecto-ADP-ribosyltransferase 3	ART3	8.23	3	3	5	389	43.9	6.06	0.634	0.729	0.641	0.718
O75144	ICOS ligand	ICOSLG	21.52	5	5	24	302	33.3	5.31	0.535	0.729	0.541	0.718
P40925	Malate dehydrogenase, cytoplasmic	MDH1	6.89	2	2	4	334	36.4	7.36	0.688	0.731	0.696	0.720
A0A0B4J1	Immunoglobulin heavy variable 3-74	IGHV3-74	37.61	2	4	5	117	12.8	8.66	2.259	0.731	2.283	0.720
Q8NBJ4	Golgi membrane protein 1	GOLM1	48.63	19	19	57	401	45.3	4.97	0.562	0.732	0.568	0.721
Q9Y653	Adhesion G-protein coupled receptor G1	ADGRG1	3.90	2	2	3	693	77.7	8.48	0.389	0.732	0.394	0.721
Q13449	Limbic system-associated membrane protein	LSAMP	10.95	3	3	8	338	37.4	6.98	0.390	0.734	0.394	0.723
P13987	CD59 glycoprotein	CD59	28.13	6	6	597	128	14.2	6.48	0.645	0.734	0.652	0.723
Q9HD42	Charged multivesicular body protein 1a	CHMP1A	8.67	2	2	3	196	21.7	8.06	1.335	0.735	1.350	0.723
P13521	Secretogranin-2	SCG2	3.73	2	2	16	617	70.9	4.75	1.268	0.735	1.282	0.723
O75071	EF-hand calcium-binding domain-containing protein 14	EFCAB14	10.71	3	3	5	495	55.0	6.32	0.618	0.735	0.624	0.724
Q99816	Tumor susceptibility gene 101 protein	TSG101	18.72	8	8	13	390	43.9	6.46	0.970	0.737	0.980	0.726
Q6P9A2	Polypeptide N-acetylgalactosaminyltransferase 18	GALNT18	2.64	2	2	2	607	69.5	6.49	0.753	0.737	0.762	0.726
P09603	Macrophage colony-stimulating factor 1	CSF1	20.76	10	10	35	554	60.1	5.29	0.860	0.741	0.869	0.729
Q06418	Tyrosine-protein kinase receptor TYRO3	TYRO3	2.81	2	2	6	890	96.8	5.67	0.734	0.746	0.742	0.734
O00182	Galectin-9	LGALS9	16.06	4	4	9	355	39.5	9.17	0.859	0.746	0.868	0.734
P21926	CD9 antigen	CD9	21.49	4	4	17	228	25.4	7.15	0.711	0.746	0.719	0.735
P08962	CD63 antigen	CD63	7.56	2	2	6	238	25.6	7.81	0.668	0.747	0.675	0.735
Q9BVM4	Gamma-glutamylaminecyclotransferase	GGACT	24.84	2	2	2	153	17.3	6.87	0.963	0.747	0.973	0.736
Q8N271	Prominin-2	PROM2	12.11	8	8	10	834	91.8	6.15	0.773	0.748	0.781	0.736
Q9NR99	Matrix-remodeling-associated protein 5	MXRA5	0.99	3	3	4	2828	312.0	8.32	0.791	0.748	0.799	0.737
P00568	Adenylate kinase isoenzyme 1	AK1	9.79	2	2	2	194	21.6	8.63	0.659	0.749	0.667	0.737
P54760	Ephrin type-B receptor 4	EPHB4	7.80	6	6	12	987	108.2	6.90	1.106	0.749	1.118	0.738
P05067	Amyloid-beta precursor protein	APP	19.48	11	12	37	770	86.9	4.82	0.579	0.750	0.585	0.739
P08138	Tumor necrosis factor receptor superfamily member 16	NGFR	9.60	3	3	9	427	45.2	4.70	0.731	0.752	0.738	0.741
P34913	Bifunctional epoxide hydrolase 2	EPHX2	9.01	3	3	4	555	62.6	6.28	0.637	0.752	0.644	0.741
Q9NPG4	Protocadherin-12	PCDH12	4.73	4	4	6	1184	128.9	5.29	0.761	0.752	0.770	0.741
P43121	Cell surface glycoprotein MUC18	MCAM	17.18	9	9	23	646	71.6	5.76	1.077	0.753	1.088	0.741
Q9Y287	Integral membrane protein 2B	ITM2B	30.83	6	6	14	266	30.3	5.14	0.667	0.753	0.674	0.741
P55290	Cadherin-13	CDH13	18.79	12	12	59	713	78.2	4.98	0.905	0.753	0.914	0.742
P0DJD8	Pepsin A-3	PGA3	10.82	5	5	15	388	41.9	4.41	1.092	0.755	1.104	0.744
P22105	Tenascin-X	TNXB	18.83	36	36	88	4244	458.1	5.17	0.815	0.755	0.823	0.744
Q99574	Neuroserpin	SERPINI1	23.17	9	9	14	410	46.4	4.91	0.714	0.755	0.722	0.744

Q9UQV4	Lysosome-associated membrane glycoprotein 3	LAMP3	8.41	3	3	3	416	44.3	8.41	1.372	0.755	1.386	0.744
P10586	Receptor-type tyrosine-protein phosphatase F	PTPRF	3.36	6	6	9	1907	212.7	6.30	0.578	0.757	0.585	0.745
Q9UIB8	SLAM family member 5	CD84	19.42	5	5	6	345	38.8	7.06	0.730	0.758	0.738	0.746
Q8NFT8	Delta and Notch-like epidermal growth factor-related receptor	DNER	5.97	3	3	4	737	78.4	5.17	0.666	0.759	0.673	0.747
P07477	Trypsin-1	PRSS1	11.34	2	2	2	247	26.5	6.51	0.954	0.759	0.964	0.747
Q9HB40	Retinoid-inducible serine carboxypeptidase	SCPEP1	16.59	7	7	26	452	50.8	5.81	0.866	0.759	0.875	0.748
P35527	Keratin, type I cytoskeletal 9	KRT9	35.63	15	16	42	623	62.0	5.24	1.454	0.759	1.470	0.748
Q03154	Aminoacylase-1	ACY1	48.77	14	14	30	408	45.9	6.18	1.062	0.761	1.073	0.749
Q00796	Sorbitol dehydrogenase	SORD	9.24	2	2	2	357	38.3	7.97	1.184	0.761	1.197	0.750
P48745	CCN family member 3	CCN3	13.45	4	4	8	357	39.1	7.72	0.740	0.762	0.748	0.750
Q13228	Methanethiol oxidase	SELENBP1	35.17	13	13	33	472	52.4	6.37	0.853	0.762	0.862	0.751
Q05707	Collagen alpha-1(XIV) chain	COL14A1	6.18	8	8	15	1796	193.4	5.30	0.985	0.763	0.996	0.751
Q9UBX1	Cathepsin F	CTSF	3.51	2	2	2	484	53.3	8.22	0.837	0.764	0.846	0.752
Q86UD1	Out at first protein homolog	OAF	8.42	2	2	6	273	30.7	6.84	0.930	0.764	0.940	0.753
Q06828	Fibromodulin	FMOD	11.17	2	2	2	376	43.2	6.04	0.534	0.765	0.540	0.753
Q01973	Inactive tyrosine-protein kinase transmembrane receptor ROR1	ROR1	3.42	2	3	4	937	104.2	7.17	1.381	0.767	1.395	0.755
Q14126	Desmoglein-2	DSG2	6.53	6	6	12	1118	122.2	5.24	0.833	0.768	0.842	0.756
P06870	Kallikrein-1	KLK1	45.42	7	7	209	262	28.9	4.83	0.608	0.769	0.614	0.758
P53990	IST1 homolog	IST1	27.47	11	11	26	364	39.7	5.35	0.794	0.771	0.803	0.759
Q9UMR5	Lysosomal thioesterase PPT2	PPT2	7.62	2	2	2	302	34.2	6.33	0.973	0.771	0.983	0.759
Q9Y6W3	Calpain-7	CAPN7	25.71	18	18	39	813	92.6	7.65	1.055	0.771	1.066	0.759
Q9NRX4	14 kDa phosphohistidine phosphatase	PHPT1	38.40	4	4	9	125	13.8	6.07	0.863	0.771	0.872	0.760
Q92859	Neogenin	NEO1	7.26	8	8	15	1461	159.9	6.54	0.504	0.771	0.510	0.760
Q9UQN3	Charged multivesicular body protein 2b	CHMP2B	27.23	8	8	19	213	23.9	8.76	0.760	0.772	0.768	0.761
Q6UWP8	Suprabasin	SBSN	31.02	9	9	18	590	60.5	7.01	1.387	0.773	1.401	0.761
Q8TB96	T-cell immunomodulatory protein	ITFG1	9.31	5	5	9	612	68.1	5.39	0.699	0.773	0.706	0.762
P16035	Metalloproteinase inhibitor 2	TIMP2	23.64	6	6	13	220	24.4	7.49	0.853	0.773	0.862	0.762
P39060	Collagen alpha-1(XVIII) chain	COL18A1	6.78	8	8	34	1754	178.1	6.01	0.795	0.774	0.803	0.763
P30530	Tyrosine-protein kinase receptor UFO	AXL	12.86	8	8	43	894	98.3	5.39	0.594	0.774	0.601	0.763
Q9NZH0	G-protein coupled receptor family C group 5 member B	GPRC5B	5.96	2	2	14	403	44.8	8.24	0.660	0.774	0.667	0.763
Q02818	Nucleobindin-1	NUCB1	52.06	19	19	31	461	53.8	5.25	0.605	0.775	0.611	0.764
P04156	Major prion protein	PRNP	15.81	4	4	23	253	27.6	9.00	0.667	0.777	0.674	0.766
Q9UBP4	Dickkopf-related protein 3	DKK3	7.43	2	2	2	350	38.4	4.65	0.639	0.777	0.645	0.766
Q8NC42	E3 ubiquitin-protein ligase RNF149	RNF149	9.00	2	2	5	400	43.1	6.54	0.939	0.778	0.949	0.766
Q9BRG1	Vacuolar protein-sorting-associated protein 25	VPS25	14.20	2	2	2	176	20.7	6.34	0.973	0.779	0.984	0.767
Q14766	Latent-transforming growth factor beta-binding protein 1	LTBP1	5.40	8	8	15	1721	186.7	5.96	0.977	0.780	0.987	0.769
P02452	Collagen alpha-1(I) chain	COL1A1	8.06	9	10	29	1464	138.9	5.80	1.243	0.781	1.256	0.769
Q09328	Alpha-1,6-mannosylglycoprotein 6-beta-N acetylglucosaminyltransferase A	MGAT5	2.43	2	2	6	741	84.5	8.12	0.885	0.781	0.895	0.770
Q5VW32	BRO1 domain-containing protein BROX	BROX	22.38	7	7	24	411	46.4	7.65	0.841	0.782	0.850	0.770
Q6EMK4	Vasorin	VASN	26.30	11	11	150	673	71.7	7.39	0.914	0.782	0.924	0.770
P20333	Tumor necrosis factor receptor superfamily member 1B	TNFRSF1B	4.56	2	2	2	461	48.3	6.28	0.828	0.784	0.837	0.772
P27930	Interleukin-1 receptor type 2	IL1R2	13.07	4	4	6	398	45.4	7.90	0.972	0.784	0.982	0.772
P55285	Cadherin-6	CDH6	8.23	6	6	14	790	88.3	4.93	0.951	0.786	0.962	0.774
P08833	Insulin-like growth factor-binding protein 1	IGFBP1	9.65	2	2	3	259	27.9	5.19	1.014	0.788	1.025	0.776
Q3LXA3	Triokinase/FMN cyclase	TKFC	14.43	5	5	6	575	58.9	7.49	0.832	0.790	0.841	0.778
Q13621	Solute carrier family 12 member 1	SLC12A1	6.73	7	7	14	1099	121.4	7.39	0.730	0.790	0.738	0.778
Q8IUL8	Cartilage intermediate layer protein 2	CILP2	10.03	12	12	25	1156	126.2	8.22	0.733	0.792	0.741	0.780
P09417	Dihydropteridine reductase	QDPR	15.16	3	3	5	244	25.8	7.37	0.803	0.795	0.811	0.783
Q7Z7D3	V-set domain-containing T-cell activation inhibitor 1	VTCN1	12.77	4	4	6	282	30.9	5.31	0.615	0.795	0.621	0.783
O43633	Charged multivesicular body protein 2a	CHMP2A	23.87	5	5	14	222	25.1	5.97	1.390	0.796	1.405	0.784
Q969P0	Immunoglobulin superfamily member 8	IGSF8	13.21	7	7	15	613	65.0	8.00	1.062	0.797	1.074	0.784
Q14254	Flotillin-2	FLOT2	10.98	4	4	7	428	47.0	5.25	1.407	0.797	1.422	0.785
Q9UN70	Protocadherin gamma-C3	PCDHGC3	14.99	10	10	20	934	101.0	5.21	0.577	0.797	0.583	0.785
Q9H665	IGF-like family receptor 1	IGFLR1	15.77	4	4	7	355	37.9	7.08	0.751	0.798	0.759	0.786
P36871	Phosphoglucomutase-1	PGM1	8.19	4	4	6	562	61.4	6.76	0.928	0.799	0.938	0.787
Q9H8L6	Multimerin-2	MMRN2	6.22	4	4	22	949	104.3	5.86	0.739	0.799	0.747	0.787
P80303	Nucleobindin-2	NUCB2	29.05	10	10	23	420	50.2	5.12	0.091	0.800	0.092	0.788
P55259	Pancreatic secretory granule membrane major glycoprotein GP2	GP2	7.26	4	4	4	537	59.4	5.24	0.524	0.801	0.529	0.789
Q8IWU5	Extracellular sulfatase Sulf-2	SULF2	2.53	2	2	115	870	100.4	9.17	0.857	0.802	0.866	0.790
Q9NY25	C-type lectin domain family 5 member A	CLEC5A	9.57	2	2	2	188	21.5	8.81	0.533	0.803	0.538	0.791
Q02747	Guanylin	GUCA2A	18.26	3	3	9	115	12.4	4.59	1.235	0.803	1.248	0.791
Q13308	Inactive tyrosine-protein kinase 7	PTK7	11.21	8	8	11	1070	118.3	7.09	0.979	0.803	0.989	0.791
Q969Z4	Tumor necrosis factor receptor superfamily member 19L	RELT	10.47	4	4	13	430	46.1	8.35	0.691	0.804	0.698	0.792
Q16787	Laminin subunit alpha-3	LAMA3	0.63	2	2	3	3333	366.4	7.24	1.200	0.804	1.213	0.792
P11586	C-1-tetrahydrofolate synthase, cytoplasmic	MTHFD1	2.67	2	2	2	935	101.5	7.30	1.262	0.805	1.275	0.793
Q7LBR1	Charged multivesicular body protein 1b	CHMP1B	12.06	3	3	6	199	22.1	8.10	0.912	0.808	0.922	0.795
Q06481	Amyloid-like protein 2	APLP2	21.49	12	13	20	763	86.9	4.79	0.947	0.809	0.957	0.797
P11597	Cholesteryl ester transfer protein	CETP	15.21	6	6	16	493	54.7	6.09	1.200	0.809	1.213	0.797
Q14393	Growth arrest-specific protein 6	GAS6	16.81	9	9	14	678	74.9	5.69	0.647	0.810	0.654	0.798
Q9NY97	N-acetyllactosaminide beta-1,3-N-acetylglucosaminyltransferase 2	B3GNT2	15.87	5	5	12	397	46.0	8.54	0.992	0.811	1.003	0.799
Q6UXG3	CMRF35-like molecule 9	CD300LG	12.35	3	3	97	332	36.0	5.92	0.798	0.811	0.807	0.799
Q15274	Nicotinate-nucleotide pyrophosphorylase [carboxylating]	QPRT	21.21	5	5	13	297	30.8	6.21	0.787	0.811	0.796	0.799
P05155	Plasma protease C1 inhibitor	SERPING1	38.20	18	18	207	500	55.1	6.55	0.806	0.811	0.815	0.799
Q5JXA9	Signal-regulatory protein beta-2	SIRPB2	6.14	2	2	2	342	36.9	5.66	0.559	0.812	0.565	0.800
O95336	6-phosphogluconolactonase	PGLS	52.71	8	8	21	258	27.5	6.05	0.804	0.813	0.813	0.801
Q9NQ79	Cartilage acidic protein 1	CRTAC1	3.48	3	3	5	661	71.4	5.12	0.657	0.814	0.664	0.801
P98161	Polycystin-1	PKD1	0.56	2	2	2	4303	462.2	6.73	0.896	0.814	0.906	0.802
Q14118	Dystroglycan	DAG1	13.07	8	8	17	895	97.4	8.56	0.583	0.815	0.589	0.803
Q15375	Ephrin type-A receptor 7	EPHA7	8.42	7	7	14	998	112.0	5.80	0.904	0.816	0.914	0.803
Q9H299	SH3 domain-binding glutamic acid-rich-like protein 3	SH3BGRL3	39.78	5	5	34	93	10.4	4.93	1.166	0.817	1.178	0.804
P14151	L-selectin	SELL	12.10	4	4	7	372	42.2	6.60	1.096	0.817	1.108	0.804
P78504	Protein jagged-1	JAG1	2.55	3	3	3	1218	133.7	6.06	1.227	0.817	1.240	0.805
Q99538	Legumain	LGMN	12.47	4	4	7	433	49.4	6.55	0.853	0.818	0.862	0.805
Q15904	V-type proton ATPase subunit S1	ATP6AP1	4.68	2	2	4	470	52.0	6.14	0.651	0.818	0.658	0.805
Q7Z7H5	Transmembrane emp24 domain-containing protein 4	TMED4	14.98	2	2	3	227	25.9	8.28	0.737	0.820	0.745	0.808
Q08629	Testican-1	SPOCK1	6.15	2	2	2	439	49.1	6.10	0.734	0.821	0.742	0.809
Q9UM47	Neurogenic locus notch homolog protein 3	NOTCH3	1.16	3	3	3	2321	243.5	5.39	1.297	0.821	1.311	0.809
O75882	Attractin	ATRN	19.38	24	24	78	1429	158.4	7.31	0.836	0.821	0.845	0.809
O75369	Filamin-B	FLNB	2.96	6	6	19	2602	278.0	5.73	0.855	0.822	0.864	0.809

Q14315	Filamin-C	FLNC	4.95	11	11	28	2725	290.8	5.97	0.917	0.823	0.926	0.810
Q8IYS5	Osteoclast-associated immunoglobulin-like receptor	OSCAR	22.34	4	4	6	282	30.5	6.52	0.496	0.823	0.501	0.810
Q8N8N7	Prostaglandin reductase 2	PTGR2	5.70	2	2	2	351	38.5	5.41	1.091	0.823	1.102	0.811
P20930	Filaggrin	FLG	9.73	10	10	16	4061	434.9	9.25	1.082	0.823	1.094	0.811
Q9Y4L1	Hypoxia up-regulated protein 1	HYOU1	3.70	3	3	3	999	111.3	5.22	0.888	0.824	0.897	0.812
P54753	Ephrin type-B receptor 3	EPHB3	6.71	6	6	9	998	110.3	6.32	1.241	0.826	1.254	0.813
Q99519	Sialidase-1	NEU1	12.29	5	5	9	415	45.4	5.88	1.301	0.826	1.315	0.814
Q8IWA5	Choline transporter-like protein 2	SLC44A2	21.25	11	11	20	706	80.1	8.57	0.863	0.826	0.873	0.814
Q12929	Epidermal growth factor receptor kinase substrate 8	EPS8	14.60	8	8	14	822	91.8	7.50	1.237	0.828	1.250	0.815
Q16270	Insulin-like growth factor-binding protein 7	IGFBP7	51.77	13	13	148	282	29.1	7.90	0.677	0.829	0.685	0.816
P10253	Lysosomal alpha-glucosidase	GAA	35.71	24	24	345	952	105.3	6.00	1.142	0.829	1.154	0.817
P05543	Thyroxine-binding globulin	SERPINA7	46.51	15	15	71	415	46.3	6.30	0.993	0.831	1.004	0.819
Q9H444	Charged multivesicular body protein 4b	CHMP4B	35.71	6	6	9	224	24.9	4.82	1.232	0.834	1.245	0.822
P11117	Lysosomal acid phosphatase	ACP2	25.53	9	9	27	423	48.3	6.74	0.992	0.834	1.003	0.822
P32004	Neural cell adhesion molecule L1	L1CAM	9.55	10	10	15	1257	139.9	6.24	0.970	0.835	0.981	0.822
Q53RD9	Fibulin-7	FBLN7	7.29	3	3	5	439	47.3	7.62	0.840	0.838	0.849	0.825
O15393	Transmembrane protease serine 2	TMPRSS2	14.84	6	6	10	492	53.8	7.81	0.620	0.838	0.627	0.825
P26842	CD27 antigen	CD27	15.00	3	3	6	260	29.1	7.64	0.746	0.839	0.754	0.826
P08637	Low affinity immunoglobulin gamma Fc region receptor III-A	FCGR3A	11.42	3	3	17	254	29.1	8.07	0.733	0.839	0.741	0.826
P42702	Leukemia inhibitory factor receptor	LIFR	1.73	2	2	3	1097	123.7	5.72	1.008	0.839	1.018	0.827
Q9P2B2	Prostaglandin F2 receptor negative regulator	PTGFRN	4.78	4	4	8	879	98.5	6.61	0.811	0.840	0.820	0.827
Q6UX71	Plexin domain-containing protein 2	PLXDC2	5.48	4	4	10	529	59.5	6.46	1.116	0.840	1.128	0.828
P01133	Pro-epidermal growth factor	EGF	37.95	35	35	296	1207	133.9	5.85	0.719	0.841	0.727	0.828
Q96AP7	Endothelial cell-selective adhesion molecule	ESAM	13.08	4	4	6	390	41.2	9.32	1.464	0.841	1.480	0.828
Q02487	Desmocollin-2	DSC2	12.21	8	8	16	901	99.9	5.34	0.678	0.841	0.685	0.829
P00441	Superoxide dismutase [Cu-Zn]	SOD1	38.31	3	3	17	154	15.9	6.13	1.109	0.842	1.121	0.829
O14498	Immunoglobulin superfamily containing leucine-rich repeat protein	ISLR	12.15	4	4	8	428	46.0	5.15	0.842	0.842	0.851	0.830
Q9NQ84	G-protein coupled receptor family C group 5 member C	GPRC5C	20.41	7	7	35	441	48.2	8.43	0.710	0.842	0.718	0.830
P34896	Serine hydroxymethyltransferase, cytosolic	SHMT1	15.94	5	5	9	483	53.0	7.71	1.205	0.842	1.218	0.830
Q9HCU0	Endosialin	CD248	12.55	8	8	45	757	80.8	5.35	0.868	0.844	0.877	0.831
P14314	Glucosidase 2 subunit beta	PRKCSH	18.18	8	8	22	528	59.4	4.41	0.650	0.844	0.657	0.832
Q9UN74	Protocadherin alpha-4	PCDHA4	3.91	3	3	5	947	102.2	5.08	0.831	0.845	0.840	0.832
Q8WWA0	Intelectin-1	ITLN1	14.70	3	3	5	313	34.9	6.01	0.416	0.845	0.420	0.832
Q6UX15	Layilin	LAYN	10.99	4	4	10	382	43.1	4.94	1.121	0.845	1.133	0.832
Q9H2M3	S-methylmethionine--homocysteine S-methyltransferase BHMT2	BHMT2	23.14	5	7	10	363	40.3	5.87	1.671	0.845	1.689	0.832
P26992	Ciliary neurotrophic factor receptor subunit alpha	CNTFR	34.41	8	8	27	372	40.6	6.76	0.921	0.845	0.931	0.832
Q8TBP5	Membrane protein FAM174A	FAM174A	17.37	2	2	3	190	19.9	6.33	0.588	0.847	0.594	0.834
Q14847	LIM and SH3 domain protein 1	LASP1	15.33	4	4	4	261	29.7	7.05	0.627	0.848	0.633	0.835
P09488	Glutathione S-transferase Mu 1	GSTM1	18.35	5	5	10	218	25.7	6.70	0.703	0.849	0.711	0.836
Q9Y5Y7	Lymphatic vessel endothelial hyaluronic acid receptor 1	LYVE1	14.60	6	6	30	322	35.2	8.28	1.707	0.849	1.725	0.837
Q08257	Quinone oxidoreductase	CRYZ	27.96	6	6	11	329	35.2	8.44	1.113	0.850	1.125	0.837
P58499	Protein FAM3B	FAM3B	27.23	5	5	9	235	26.0	8.75	0.809	0.850	0.817	0.838
P63241	Eukaryotic translation initiation factor 5A-1	EIF5A	18.18	3	3	4	154	16.8	5.24	0.682	0.851	0.689	0.838
Q08174	Protocadherin-1	PCDH1	13.87	10	10	16	1060	114.7	5.03	0.493	0.852	0.499	0.839
P04264	Keratin, type II cytoskeletal 1	KRT1	43.01	22	26	88	644	66.0	8.12	1.911	0.852	1.931	0.840
O00159	Unconventional myosin-Ic	MYO1C	5.55	4	4	6	1063	121.6	9.41	1.066	0.853	1.077	0.840
Q9ULK6	RING finger protein 150	RNF150	6.85	3	3	5	438	48.0	5.27	0.757	0.853	0.765	0.840
P84095	Rho-related GTP-binding protein RhoG	RHOG	24.61	3	3	3	191	21.3	8.12	1.546	0.854	1.563	0.841
Q9Y624	Junctional adhesion molecule A	F11R	21.40	4	4	6	299	32.6	7.90	1.173	0.855	1.185	0.842
P09467	Fructose-1,6-bisphosphatase 1	FBP1	31.36	8	8	14	338	36.8	6.99	0.953	0.855	0.963	0.842
O43291	Kunitz-type protease inhibitor 2	SPINT2	8.73	2	2	18	252	28.2	8.29	0.601	0.855	0.608	0.842
Q06830	Peroxiredoxin-1	PRDX1	36.18	8	8	14	199	22.1	8.13	0.861	0.856	0.871	0.843
P23284	Peptidyl-prolyl cis-trans isomerase B	PPIB	27.78	6	6	12	216	23.7	9.41	0.606	0.856	0.612	0.843
P14784	Interleukin-2 receptor subunit beta	IL2RB	5.99	2	2	2	551	61.1	5.05	1.074	0.856	1.086	0.843
P01042	Kininogen-1	KNG1	40.99	33	33	1435	644	71.9	6.81	0.704	0.856	0.712	0.843
P16083	Ribosyldihydronicotinamide dehydrogenase [quinone]	NQO2	11.69	2	2	2	231	25.9	6.29	1.221	0.858	1.234	0.845
P33151	Cadherin-5	CDH5	2.42	2	2	2	784	87.5	5.43	0.954	0.859	0.964	0.846
P27824	Calnexin	CANX	9.63	5	5	12	592	67.5	4.60	1.112	0.860	1.124	0.847
P01036	Cystatin-S	CST4	61.70	6	6	19	141	16.2	5.02	0.139	0.860	0.140	0.847
Q8N114	Protein shisa-5	SHISA5	3.33	2	2	44	240	25.6	6.68	1.217	0.861	1.230	0.848
O43278	Kunitz-type protease inhibitor 1	SPINT1	18.90	9	9	18	529	58.4	6.29	0.798	0.861	0.806	0.848
P35052	Glypican-1	GPC1	21.15	8	8	17	558	61.6	7.30	0.745	0.862	0.752	0.849
Q5TFQ8	Signal-regulatory protein beta-1 isoform 3	SIRPB1	32.16	2	9	14	398	43.3	7.83	0.473	0.862	0.478	0.849
Q6YHK3	CD109 antigen	CD109	1.59	2	2	2	1445	161.6	5.85	0.719	0.863	0.727	0.850
Q8N4F0	BPI fold-containing family B member 2	BPIFB2	22.05	6	6	10	458	49.1	8.72	0.136	0.864	0.137	0.851
Q6V0I7	Protocadherin Fat 4	FAT4	4.22	15	15	21	4981	542.4	4.94	0.728	0.864	0.736	0.851
Q8NHL6	Leukocyte immunoglobulin-like receptor subfamily B member 1	LILRB1	6.15	3	3	7	650	70.8	6.05	1.031	0.865	1.042	0.852
P10645	Chromogranin-A	CHGA	6.13	2	2	34	457	50.7	4.60	0.811	0.866	0.820	0.853
O00499	Myc box-dependent-interacting protein 1	BIN1	4.72	2	2	2	593	64.7	5.06	0.523	0.866	0.529	0.853
P00352	Retinal dehydrogenase 1	ALDH1A1	13.57	6	6	14	501	54.8	6.73	0.896	0.869	0.905	0.856
O94910	Adhesion G protein-coupled receptor L1	ADGRL1	4.07	4	4	10	1474	162.6	6.60	0.869	0.869	0.878	0.856
P10153	Non-secretory ribonuclease	RNASE2	21.12	4	4	129	161	18.3	8.73	1.084	0.870	1.096	0.856
P19022	Cadherin-2	CDH2	16.89	8	8	17	906	99.7	4.81	0.688	0.870	0.695	0.857
P48960	CD97 antigen	CD97	2.75	2	2	3	835	91.8	6.87	1.541	0.871	1.558	0.858
Q9NZZ3	Charged multivesicular body protein 5	CHMP5	27.40	4	4	5	219	24.6	4.83	0.657	0.871	0.664	0.858
P55957	BH3-interacting domain death agonist	BID	27.69	4	4	7	195	22.0	5.44	0.857	0.872	0.866	0.859
P13284	Gamma-interferon-inducible lysosomal thiol reductase	IFI30	13.20	3	3	43	250	27.9	4.88	1.181	0.872	1.193	0.859
Q8WVN6	Secreted and transmembrane protein 1	SECTM1	20.97	3	3	212	248	27.0	7.43	1.006	0.872	1.017	0.859
O00462	Beta-mannosidase	MANBA	17.63	13	13	26	879	100.8	5.52	0.893	0.873	0.902	0.860
P08572	Collagen alpha-2(IV) chain	COL4A2	5.43	7	7	17	1712	167.4	8.66	0.823	0.875	0.832	0.861
P52565	Rho GDP-dissociation inhibitor 1	ARHGDIA	12.75	2	2	3	204	23.2	5.11	0.885	0.875	0.894	0.861
Q9Y696	Chloride intracellular channel protein 4	CLIC4	15.42	2	2	2	253	28.8	5.59	1.037	0.875	1.048	0.862
P00966	Argininosuccinate synthase	ASS1	22.82	9	9	15	412	46.5	8.02	0.808	0.875	0.817	0.862
P20711	Aromatic-L-amino-acid decarboxylase	DDC	3.96	2	2	2	480	53.9	7.20	0.676	0.876	0.683	0.863
P20062	Transcobalamin-2	TCN2	20.14	4	4	5	427	47.5	7.01	0.947	0.876	0.957	0.863
Q7Z3B1	Neuronal growth regulator 1	NEGR1	32.20	8	8	34	354	38.7	6.21	0.761	0.876	0.769	0.863
Q99835	Smoothened homolog	SMO	2.03	2	2	2	787	86.3	8.34	0.883	0.878	0.893	0.865
Q14515	SPARC-like protein 1	SPARCL1	27.11	14	14	67	664	75.2	4.81	0.804	0.878	0.813	0.865
O60888	Protein CutA	CUTA	25.70	4	4	13	179	19.1	5.50	0.909	0.878	0.918	0.865

Q5JS37	NHL repeat-containing protein 3	NHLRC3	26.80	5	5	7	347	38.3	6.43	1.253	0.878	1.267	0.865
Q99715	Collagen alpha-1(XII) chain	COL12A1	7.31	15	15	44	3063	332.9	5.53	0.810	0.880	0.819	0.866
Q9BUT1	3-hydroxybutyrate dehydrogenase type 2	BDH2	13.47	3	3	7	245	26.7	7.65	0.624	0.880	0.630	0.866
Q86XT2	Vacuolar protein sorting-associated protein 37D	VPS37D	6.77	2	2	3	251	27.7	9.01	0.992	0.880	1.003	0.867
Q13145	BMP and activin membrane-bound inhibitor homolog	BAMBI	7.31	2	2	4	260	29.1	7.75	1.107	0.881	1.119	0.867
P11766	Alcohol dehydrogenase class-3	ADH5	10.43	3	3	4	374	39.7	7.49	0.989	0.881	1.000	0.868
O43490	Prominin-1	PROM1	18.61	13	13	30	865	97.1	7.27	0.966	0.881	0.976	0.868
P08887	Interleukin-6 receptor subunit alpha	IL6R	4.70	2	2	2	468	51.5	8.22	1.026	0.881	1.037	0.868
P35555	Fibrillin-1	FBN1	10.83	26	26	70	2871	312.1	4.93	0.768	0.883	0.777	0.869
Q96MU8	Kremen protein 1	KREMEN1	5.92	2	2	3	473	51.7	7.11	0.513	0.883	0.518	0.869
Q9BZZ2	Sialoadhesin	SIGLEC1	3.22	5	5	7	1709	182.5	6.62	0.987	0.883	0.998	0.870
P11233	Ras-related protein Ral-A	RALA	16.50	3	3	6	206	23.6	7.11	1.102	0.883	1.114	0.870
Q8N2S1	Latent-transforming growth factor beta-binding protein 4	LTBP4	3.08	4	4	7	1624	173.3	5.43	0.803	0.883	0.811	0.870
Q9UQB8	Brain-specific angiogenesis inhibitor 1-associated protein 2	BAIAP2	13.22	6	6	11	552	60.8	8.90	0.967	0.885	0.978	0.871
P30041	Peroxiredoxin-6	PRDX6	29.02	5	5	9	224	25.0	6.38	0.721	0.885	0.729	0.871
P23526	Adenosylhomocysteinase	AHCY	15.97	5	5	9	432	47.7	6.34	1.020	0.885	1.031	0.872
P11047	Laminin subunit gamma-1	LAMC1	5.03	7	7	18	1609	177.5	5.12	0.740	0.886	0.748	0.872
P19961	Alpha-amylase 2B	AMY2B	48.14	3	17	369	511	57.7	7.09	0.798	0.886	0.806	0.873
Q92563	Testican-2	SPOCK2	8.73	4	4	5	424	46.7	4.83	1.026	0.887	1.037	0.874
Q01459	Di-N-acetylchitobiase	CTBS	36.88	11	11	44	385	43.7	6.64	1.345	0.888	1.359	0.874
Q96MM7	Heparan-sulfate 6-O-sulfotransferase 2	HS6ST2	3.97	2	2	2	605	69.1	9.73	1.304	0.889	1.318	0.876
Q92820	Gamma-glutamyl hydrolase	GGH	36.16	11	11	98	318	35.9	7.11	0.854	0.889	0.863	0.876
P78324	Tyrosine-protein phosphatase non-receptor type substrate 1	SIRPA	35.12	5	15	51	504	54.9	6.98	1.010	0.889	1.021	0.876
P18827	Syndecan-1	SDC1	11.61	2	2	4	310	32.4	4.63	0.911	0.890	0.921	0.876
P29966	Myristoylated alanine-rich C-kinase substrate	MARCKS	26.20	5	5	11	332	31.5	4.45	0.780	0.890	0.788	0.877
P13591	Neural cell adhesion molecule 1	NCAM1	7.93	4	4	11	858	94.5	4.87	1.040	0.892	1.052	0.879
P16278	Beta-galactosidase	GLB1	21.42	11	11	66	677	76.0	6.57	0.989	0.893	1.000	0.879
O60279	Sushi domain-containing protein 5	SUSD5	7.95	4	4	13	629	68.0	4.91	0.671	0.893	0.678	0.880
P35558	Phosphoenolpyruvate carboxykinase, cytosolic [GTP]	PCK1	9.97	6	6	10	622	69.1	6.14	1.128	0.894	1.141	0.880
P51654	Glypican-3	GPC3	12.76	6	6	13	580	65.5	6.37	0.869	0.894	0.879	0.881
Q96MG2	Junctional sarcoplasmic reticulum protein 1	JSRP1	11.78	3	3	5	331	36.3	9.38	0.372	0.895	0.376	0.881
P05090	Apolipoprotein D	APOD	47.62	10	10	372	189	21.3	5.15	1.143	0.895	1.155	0.881
P29972	Aquaporin-1	AQP1	19.70	2	2	6	269	28.5	7.42	0.325	0.895	0.329	0.882
Q9Y646	Carboxypeptidase Q	CPQ	27.54	10	10	32	472	51.9	6.18	1.010	0.896	1.021	0.882
O94760	N(G),N(G)-dimethylarginine dimethylaminohydrolase 1	DDAH1	22.81	5	6	14	285	31.1	5.81	1.266	0.896	1.280	0.883
P0DP57	Secreted Ly-6/uPAR domain-containing protein 2	SLURP2	24.74	2	2	18	97	10.2	6.62	0.679	0.897	0.686	0.884
P20138	Myeloid cell surface antigen CD33	CD33	11.26	3	3	8	364	39.8	8.38	0.591	0.898	0.598	0.885
Q9NQS3	Nectin-3	NECTIN3	7.65	3	3	5	549	61.0	6.19	0.898	0.898	0.908	0.885
P02461	Collagen alpha-1(III) chain	COL3A1	1.57	2	2	10	1466	138.5	6.61	1.369	0.899	1.384	0.885
P20472	Parvalbumin alpha	PVALB	20.91	2	2	2	110	12.1	5.19	1.321	0.899	1.336	0.886
Q495M3	Proton-coupled amino acid transporter 2	SLC36A2	6.83	3	3	6	483	53.2	8.12	0.808	0.900	0.816	0.887
P32754	4-hydroxyphenylpyruvate dioxygenase	HPD	7.38	3	3	4	393	44.9	7.01	1.404	0.900	1.419	0.887
P09936	Ubiquitin carboxyl-terminal hydrolase isozyme L1	UCHL1	22.42	3	3	6	223	24.8	5.48	0.767	0.901	0.776	0.887
O43852	Calumenin	CALU	21.90	5	5	8	315	37.1	4.64	0.509	0.901	0.515	0.887
Q8IX04	Ubiquitin-conjugating enzyme E2 variant 3	UEVLD	14.23	5	5	8	471	52.2	7.09	0.955	0.901	0.965	0.887
Q92520	Protein FAM3C	FAM3C	39.21	7	7	18	227	24.7	8.29	1.279	0.901	1.293	0.887
Q8NDA2	Hemicentin-2	HMCN2	1.27	5	5	5	5059	541.6	5.87	0.823	0.903	0.832	0.889
P00918	Carbonic anhydrase 2	CA2	28.08	6	6	14	260	29.2	7.40	1.179	0.903	1.191	0.890
P50053	Ketohexokinase	KHK	17.45	4	4	5	298	32.5	6.32	0.875	0.903	0.885	0.890
P08185	Corticosteroid-binding globulin	SERPINA6	45.19	12	12	36	405	45.1	6.04	1.117	0.903	1.129	0.890
Q86VN1	Vacuolar protein-sorting-associated protein 36	VPS36	5.44	2	2	2	386	43.8	7.20	1.432	0.908	1.447	0.894
P06865	Beta-hexosaminidase subunit alpha	HEXA	26.09	12	12	18	529	60.7	5.16	0.791	0.909	0.800	0.895
P36543	V-type proton ATPase subunit E 1	ATP6V1E1	13.27	3	3	3	226	26.1	8.00	1.082	0.909	1.094	0.895
Q9HCM3	UPF0606 protein KIAA1549	KIAA1549	1.08	2	2	2	1950	210.6	6.11	1.043	0.913	1.054	0.899
P98095	Fibulin-2	FBLN2	7.52	5	5	16	1184	126.5	4.82	0.888	0.913	0.897	0.899
P00734	Prothrombin	F2	31.51	14	14	135	622	70.0	5.90	0.781	0.914	0.790	0.900
Q15113	Procollagen C-endopeptidase enhancer 1	PCOLCE	16.70	5	5	18	449	47.9	7.43	0.864	0.916	0.873	0.902
P08519	Apolipoprotein(a)	LPA	17.46	3	3	7	4548	501.0	5.88	0.489	0.916	0.494	0.902
P42685	Tyrosine-protein kinase FRK	FRK	5.74	3	3	4	505	58.2	6.67	1.357	0.917	1.372	0.903
Q9UK41	Vacuolar protein sorting-associated protein 28 homolog	VPS28	24.89	5	5	9	221	25.4	5.54	0.950	0.918	0.960	0.904
P16152	Carbonyl reductase [NADPH] 1	CBR1	29.96	7	7	14	277	30.4	8.32	0.897	0.918	0.906	0.904
Q92896	Golgi apparatus protein 1	GLG1	17.30	17	17	25	1179	134.5	6.90	0.677	0.919	0.684	0.905
P22732	Solute carrier family 2, facilitated glucose transporter member 5	SLC2A5	9.58	5	5	11	501	54.9	6.04	0.751	0.920	0.759	0.906
P02511	Alpha-crystallin B chain	CRYAB	10.86	2	2	2	175	20.1	7.33	1.315	0.921	1.329	0.907
P54289	Voltage-dependent calcium channel subunit alpha-2/delta-1	CACNA2D1	4.99	4	4	6	1103	124.5	5.27	0.492	0.921	0.497	0.907
Q8WUM4	Programmed cell death 6-interacting protein	PDCD6IP	28.23	23	23	43	868	96.0	6.52	0.881	0.922	0.890	0.908
P62070	Ras-related protein R-Ras2	RRAS2	9.80	2	2	3	204	23.4	6.01	0.788	0.923	0.796	0.909
Q86TH1	ADAMTS-like protein 2	ADAMTSL2	4.10	4	4	6	951	104.6	6.42	1.067	0.924	1.079	0.910
Q5SZK8	FRAS1-related extracellular matrix protein 2	FREM2	8.27	20	20	31	3169	350.9	5.03	0.780	0.924	0.788	0.910
Q12860	Contactin-1	CNTN1	23.97	22	22	44	1018	113.2	5.90	0.885	0.924	0.894	0.910
Q92673	Sortilin-related receptor	SORL1	14.36	25	25	47	2214	248.3	5.55	0.933	0.925	0.943	0.911
Q9UKY0	Prion-like protein doppel	PRND	21.02	4	4	7	176	20.3	9.03	0.992	0.925	1.003	0.911
Q14314	Fibroleukin	FGL2	4.10	2	2	4	439	50.2	7.39	0.476	0.925	0.481	0.911
Q9NP79	Vacuolar protein sorting-associated protein VTA1 homolog	VTA1	14.01	5	5	10	307	33.9	6.29	0.928	0.926	0.938	0.912
Q6UX73	UPF0764 protein C16orf89	C16orf89	10.95	4	4	12	402	45.4	6.19	0.596	0.927	0.603	0.913
P13645	Keratin, type I cytoskeletal 10	KRT10	43.32	16	17	66	584	58.8	5.21	1.979	0.927	2.000	0.913
Q8IUK5	Plexin domain-containing protein 1	PLXDC1	12.40	5	5	8	500	55.7	5.85	0.384	0.928	0.388	0.914
Q9NPH3	Interleukin-1 receptor accessory protein	IL1RAP	5.79	3	3	3	570	65.4	8.12	1.172	0.928	1.185	0.914
P13646	Keratin, type I cytoskeletal 13	KRT13	40.17	14	16	39	458	49.6	4.96	1.570	0.929	1.587	0.915
P05452	Tetranectin	CLEC3B	42.57	7	7	35	202	22.5	5.67	0.841	0.930	0.850	0.916
P02751	Fibronectin	FN1	21.19	36	36	109	2477	272.2	5.50	0.983	0.930	0.993	0.916
Q6UVK1	Chondroitin sulfate proteoglycan 4	CSPG4	6.68	11	11	26	2322	250.4	5.47	0.941	0.931	0.951	0.917
O75351	Vacuolar protein sorting-associated protein 4B	VPS4B	42.34	13	16	34	444	49.3	7.23	0.912	0.931	0.922	0.917
Q9UHR4	Brain-specific angiogenesis inhibitor 1-associated protein 2-like protein 1	BAIAP2L1	11.15	4	4	8	511	56.8	8.68	1.051	0.935	1.062	0.921
P15291	Beta-1,4-galactosyltransferase 1	B4GALT1	6.53	3	3	8	398	43.9	8.65	0.745	0.935	0.753	0.921
P08582	Melanotransferrin	MELTF	30.49	15	15	36	738	80.2	5.94	0.912	0.935	0.922	0.921
Q07507	Dermatopontin	DPT	13.93	2	2	9	201	24.0	4.82	1.431	0.935	1.446	0.921
P50895	Basal cell adhesion molecule	BCAM	16.88	7	7	22	628	67.4	5.81	0.886	0.936	0.896	0.922
P04216	Thy-1 membrane glycoprotein	THY1	24.84	4	4	14	161	17.9	8.73	1.112	0.936	1.124	0.922

O75347	Tubulin-specific chaperone A	TBCA	20.37	2	2	2	108	12.8	5.29	0.503	0.937	0.508	0.923
P05154	Plasma serine protease inhibitor	SERPINA5	48.03	18	18	122	406	45.6	9.26	0.880	0.942	0.889	0.927
O75936	Gamma-butyrobetaine dioxygenase	BBOX1	5.68	2	2	2	387	44.7	6.74	1.146	0.942	1.158	0.928
Q9UKU9	Angiopoietin-related protein 2	ANGPTL2	14.20	9	9	19	493	57.1	7.53	1.296	0.944	1.310	0.930
Q86VB7	Scavenger receptor cysteine-rich type 1 protein M130	CD163	2.08	3	3	3	1156	125.4	5.95	0.721	0.944	0.729	0.930
P22748	Carbonic anhydrase 4	CA4	5.77	2	2	2	312	35.0	7.83	0.935	0.945	0.945	0.931
Q07075	Glutamyl aminopeptidase	ENPEP	15.88	14	14	31	957	109.2	5.47	1.438	0.946	1.453	0.931
P08473	Neprilysin	MME	29.33	19	19	35	750	85.5	5.73	0.652	0.946	0.659	0.932
P30043	Flavin reductase (NADPH)	BLVRB	16.02	3	3	9	206	22.1	7.65	0.991	0.946	1.001	0.932
Q86V85	Integral membrane protein GPR180	GPR180	11.14	4	4	12	440	49.4	7.39	0.846	0.947	0.855	0.933
P18065	Insulin-like growth factor-binding protein 2	IGFBP2	30.77	7	7	12	325	34.8	7.50	0.815	0.947	0.824	0.933
P17342	Atrial natriuretic peptide receptor 3	NPR3	9.06	4	4	5	541	59.8	6.35	0.746	0.948	0.754	0.934
O75368	SH3 domain-binding glutamic acid-rich-like protein	SH3BGRL	20.18	3	3	5	114	12.8	5.25	1.786	0.949	1.805	0.935
Q9NZP8	Complement C1r subcomponent-like protein	C1RL	25.87	9	10	34	487	53.5	7.20	1.242	0.950	1.255	0.936
Q53GD3	Choline transporter-like protein 4	SLC44A4	6.06	5	5	8	710	79.2	8.59	0.669	0.951	0.676	0.937
P61970	Nuclear transport factor 2	NUTF2	70.87	5	5	73	127	14.5	5.38	1.273	0.951	1.287	0.937
Q86T13	C-type lectin domain family 14 member A	CLEC14A	21.43	7	7	13	490	51.6	6.35	0.894	0.952	0.904	0.937
P35754	Glutaredoxin-1	GLRX	38.68	3	3	7	106	11.8	8.09	1.267	0.952	1.280	0.937
P14550	Aldo-keto reductase family 1 member A1	AKR1A1	39.08	10	10	19	325	36.5	6.79	0.902	0.952	0.912	0.938
O95154	Aflatoxin B1 aldehyde reductase member 3	AKR7A3	10.57	2	3	3	331	37.2	7.15	0.837	0.952	0.846	0.938
P30044	Peroxiredoxin-5, mitochondrial	PRDX5	16.82	3	3	6	214	22.1	8.70	0.661	0.953	0.668	0.938
Q13421	Mesothelin	MSLN	10.48	5	5	10	630	68.9	6.38	1.390	0.953	1.404	0.938
P60953	Cell division control protein 42 homolog	CDC42	27.75	5	5	11	191	21.2	6.55	1.240	0.954	1.253	0.940
P21399	Cytoplasmic aconitate hydratase	ACO1	1.80	2	2	2	889	98.3	6.68	1.053	0.955	1.065	0.941
P08183	ATP-dependent translocase ABCB1	ABCB1	5.39	5	5	7	1280	141.4	9.00	1.124	0.956	1.136	0.941
Q9BRK5	45 kDa calcium-binding protein	SDF4	19.89	6	6	14	362	41.8	4.86	0.687	0.957	0.694	0.942
P35237	Serpin B6	SERPINB6	11.97	3	3	3	376	42.6	5.27	1.267	0.957	1.281	0.943
O00764	Pyridoxal kinase	PDXK	6.41	2	2	2	312	35.1	6.13	1.280	0.958	1.293	0.944
Q8NHJ6	Leukocyte immunoglobulin-like receptor subfamily B member 4	LILRB4	6.92	3	3	4	448	49.3	6.67	0.972	0.958	0.982	0.944
P16930	Fumarylacetoacetase	FAH	8.35	3	3	5	419	46.3	6.95	1.217	0.959	1.230	0.945
P04275	von Willebrand factor	VWF	2.49	5	5	10	2813	309.1	5.48	0.949	0.960	0.959	0.945
P98160	Basement membrane-specific heparan sulfate proteoglycan core protein	HSPG2	16.83	56	56	675	4391	468.5	6.51	1.072	0.960	1.083	0.945
P22792	Carboxypeptidase N subunit 2	CPN2	28.44	9	9	22	545	60.5	5.99	0.917	0.961	0.926	0.947
Q8N126	Cell adhesion molecule 3	CADM3	17.34	4	4	10	398	43.3	6.09	1.862	0.962	1.882	0.948
P20073	Annexin A7	ANXA7	17.21	6	6	16	488	52.7	5.68	0.765	0.962	0.773	0.948
P08174	Complement decay-accelerating factor	CD55	41.21	15	15	59	381	41.4	7.59	1.201	0.962	1.213	0.948
P24592	Insulin-like growth factor-binding protein 6	IGFBP6	14.17	3	3	4	240	25.3	7.81	1.135	0.963	1.147	0.949
Q9BTY2	Plasma alpha-L-fucosidase	FUCA2	6.85	4	4	6	467	54.0	6.25	0.971	0.966	0.981	0.952
Q15485	Ficolin-2	FCN2	14.70	3	3	6	313	34.0	6.77	1.337	0.966	1.352	0.952
Q99523	Sortilin	SORT1	6.02	4	4	8	831	92.0	5.74	0.762	0.967	0.771	0.953
Q9NPF0	CD320 antigen	CD320	20.21	6	6	11	282	29.0	4.75	0.761	0.969	0.769	0.955
Q06278	Aldehyde oxidase	AOX1	1.79	2	2	2	1338	147.8	7.17	1.623	0.970	1.640	0.955
P05413	Fatty acid-binding protein, heart	FABP3	34.59	5	5	9	133	14.8	6.80	0.920	0.970	0.930	0.955
Q9NQ36	Signal peptide, CUB and EGF-like domain-containing protein 2	SCUBE2	2.50	2	2	2	999	109.9	6.64	1.099	0.970	1.111	0.955
P41222	Prostaglandin-H2 D-isomerase	PTGDS	51.58	8	8	282	190	21.0	7.80	1.703	0.970	1.721	0.955
Q9H0W9	Ester hydrolase C11orf54	C11orf54	31.43	7	7	16	315	35.1	6.70	0.837	0.971	0.846	0.956
Q8WW52	Protein FAM151A	FAM151A	17.95	8	8	21	585	64.0	6.67	1.240	0.971	1.253	0.957
P63000	Ras-related C3 botulinum toxin substrate 1	RAC1	28.65	6	6	7	192	21.4	8.50	0.842	0.973	0.851	0.958
P19440	Glutathione hydrolase 1 proenzyme	GGT1	16.17	9	9	21	569	61.4	7.12	1.192	0.974	1.204	0.959
P19835	Bile salt-activated lipase	CEL	27.36	19	19	76	753	79.3	5.34	1.096	0.975	1.108	0.960
P02792	Ferritin light chain	FTL	41.14	6	6	11	175	20.0	5.78	0.236	0.975	0.238	0.960
P43251	Biotinidase	BTD	28.91	12	12	32	543	61.1	6.25	0.970	0.975	0.980	0.961
Q14344	Guanine nucleotide-binding protein subunit alpha-13	GNA13	5.57	2	2	2	377	44.0	8.00	0.959	0.975	0.969	0.961
P06280	Alpha-galactosidase A	GLA	34.27	11	11	25	429	48.7	5.60	0.881	0.976	0.890	0.962
P10619	Lysosomal protective protein	CTSA	17.50	8	8	27	480	54.4	6.61	0.927	0.977	0.937	0.962
P78417	Glutathione S-transferase omega-1	GSTO1	22.41	6	6	11	241	27.5	6.60	1.280	0.978	1.294	0.963
A0A0C4DH	Immunoglobulin heavy variable 5-51	IGHV5-51	37.61	3	3	8	117	12.7	8.27	2.244	0.978	2.268	0.963
Q8TCD5	5'(3')-deoxyribonucleotidase, cytosolic type	NT5C	16.92	2	2	3	201	23.4	6.64	1.356	0.978	1.371	0.963
Q7Z7M0	Multiple epidermal growth factor-like domains protein 8	MEGF8	8.26	17	17	23	2845	302.9	6.87	1.002	0.979	1.012	0.964
Q5VY43	Platelet endothelial aggregation receptor 1	PEAR1	3.28	3	3	3	1037	110.6	6.81	1.100	0.979	1.112	0.965
P19438	Tumor necrosis factor receptor superfamily member 1A	TNFRSF1A	10.33	4	4	7	455	50.5	6.64	1.148	0.981	1.160	0.967
O00161	Synaptosomal-associated protein 23	SNAP23	19.91	3	3	5	211	23.3	5.01	0.989	0.983	1.000	0.968
P10412	Histone H1.4	H1-4	14.16	2	4	6	219	21.9	11.03	1.117	0.984	1.129	0.969
P14625	Endoplasmin	HSP90B1	7.35	3	4	6	803	92.4	4.84	0.548	0.984	0.554	0.969
Q14767	Latent-transforming growth factor beta-binding protein 2	LTBP2	9.01	13	13	31	1821	194.9	5.19	0.946	0.984	0.956	0.969
P13473	Lysosome-associated membrane glycoprotein 2	LAMP2	8.78	4	4	39	410	44.9	5.63	1.014	0.984	1.025	0.969
P17813	Endoglin	ENG	4.26	2	2	3	658	70.5	6.61	0.721	0.985	0.729	0.970
P00558	Phosphoglycerate kinase 1	PGK1	30.94	10	10	20	417	44.6	8.10	0.819	0.985	0.828	0.970
Q9HC84	Mucin-5B	MUC5B	1.82	3	3	6	5762	596.0	6.64	0.204	0.987	0.206	0.972
Q9BY43	Charged multivesicular body protein 4a	CHMP4A	14.41	3	3	5	222	25.1	4.70	0.841	0.987	0.850	0.972
O43895	Xaa-Pro aminopeptidase 2	XPNPEP2	13.65	5	5	7	674	75.6	6.04	1.253	0.987	1.266	0.972
P10301	Ras-related protein R-Ras	RRAS	14.22	2	2	2	218	23.5	6.93	1.197	0.987	1.210	0.972
Q16661	Guanylate cyclase activator 2B	GUCA2B	18.75	3	3	5	112	12.1	6.48	3.515	0.987	3.552	0.973
P0DP24	Calmodulin-2	CALM2	53.69	7	7	22	149	16.8	4.22	0.866	0.988	0.875	0.973
P54107	Cysteine-rich secretory protein 1	CRISP1	13.65	3	3	8	249	28.5	5.91	0.084	0.990	0.085	0.975
P08571	Monocyte differentiation antigen CD14	CD14	31.47	11	11	105	375	40.1	6.23	1.492	0.991	1.508	0.976
Q9Y5C1	Angiopoietin-related protein 3	ANGPTL3	4.35	2	2	2	460	53.6	6.70	0.800	0.991	0.808	0.976
P08134	Rho-related GTP-binding protein RhoC	RHOC	17.62	4	4	6	193	22.0	6.58	1.231	0.992	1.244	0.977
P62937	Peptidyl-prolyl cis-trans isomerase A	PPIA	44.85	6	6	12	165	18.0	7.81	0.788	0.992	0.796	0.977
P01040	Cystatin-A	CSTA	75.51	7	7	21	98	11.0	5.50	1.509	0.993	1.525	0.978
P00491	Purine nucleoside phosphorylase	PNP	25.61	6	6	12	289	32.1	6.95	1.112	0.993	1.124	0.978
Q96CF2	Charged multivesicular body protein 4c	CHMP4C	7.30	2	2	4	233	26.4	6.07	0.942	0.993	0.952	0.978
O75487	Glypican-4	GPC4	13.85	6	6	17	556	62.4	6.68	0.920	0.995	0.930	0.980
P11021	Endoplasmic reticulum chaperone BiP	HSPA5	19.42	8	10	19	654	72.3	5.16	0.729	0.995	0.737	0.980
P05362	Intercellular adhesion molecule 1	ICAM1	14.29	7	7	15	532	57.8	7.99	1.098	0.996	1.110	0.981
O43451	Maltase-glucoamylase, intestinal	MGAM	21.11	34	34	130	1857	209.7	5.50	0.741	0.996	0.749	0.981
P15328	Folate receptor alpha	FOLR1	31.52	6	6	51	257	29.8	7.97	0.768	0.997	0.776	0.981
P38571	Lysosomal acid lipase/cholesteryl ester hydrolase	LIPA	6.77	2	2	2	399	45.4	6.92	1.948	0.997	1.969	0.982
P16870	Carboxypeptidase E	CPE	39.71	16	16	31	476	53.1	5.14	0.656	0.997	0.663	0.982

P40189	Interleukin-6 receptor subunit beta	IL6ST	10.13	7	7	14	918	103.5	5.95	0.949	0.998	0.959	0.983
P22692	Insulin-like growth factor-binding protein 4	IGFBP4	8.53	2	2	3	258	27.9	7.15	1.023	0.998	1.034	0.983
P61916	NPC intracellular cholesterol transporter 2	NPC2	67.55	9	9	36	151	16.6	7.65	0.538	0.999	0.543	0.984
P02765	Alpha-2-HS-glycoprotein	AHSG	43.05	12	12	313	367	39.3	5.72	1.413	0.999	1.429	0.984
P12111	Collagen alpha-3(VI) chain	COL6A3	7.77	21	21	51	3177	343.5	6.68	1.213	1.004	1.226	0.989
P32119	Peroxiredoxin-2	PRDX2	25.76	5	5	7	198	21.9	5.97	0.588	1.006	0.594	0.990
P07307	Asialoglycoprotein receptor 2	ASGR2	20.26	3	3	3	311	35.1	6.25	0.781	1.006	0.790	0.991
P15529	Membrane cofactor protein	CD46	4.08	2	2	2	392	43.7	6.74	1.671	1.007	1.689	0.991
P11717	Cation-independent mannose-6-phosphate receptor	IGF2R	8.03	15	15	27	2491	274.2	5.94	0.893	1.007	0.902	0.992
P22352	Glutathione peroxidase 3	GPX3	28.76	6	6	7	226	25.5	8.13	0.965	1.007	0.975	0.992
Q12913	Receptor-type tyrosine-protein phosphatase eta	PTPRJ	13.16	14	14	30	1337	145.9	5.58	0.786	1.007	0.794	0.992
O43505	Beta-1,4-glucuronyltransferase 1	B4GAT1	20.00	5	5	11	415	47.1	7.20	0.872	1.008	0.881	0.993
P16444	Dipeptidase 1	DPEP1	32.85	10	10	29	411	45.6	6.15	0.987	1.008	0.998	0.993
P29323	Ephrin type-B receptor 2	EPHB2	6.54	5	5	9	1055	117.4	6.55	1.232	1.008	1.246	0.993
Q9UK55	Protein Z-dependent protease inhibitor	SERPINA10	4.28	2	2	3	444	50.7	8.27	0.677	1.009	0.684	0.994
Q9UBI6	Guanine nucleotide-binding protein G(I)/G(S)/G(O) subunit gamma-12	GNG12	29.17	2	2	2	72	8.0	8.97	0.854	1.010	0.863	0.995
P23470	Receptor-type tyrosine-protein phosphatase gamma	PTPRG	5.26	5	5	16	1445	161.9	6.42	1.170	1.011	1.183	0.996
O75339	Cartilage intermediate layer protein 1	CILP	3.21	3	3	4	1184	132.5	8.41	0.762	1.012	0.770	0.997
Q9Y2S2	Lambda-crystallin homolog	CRYL1	20.69	5	5	10	319	35.4	6.18	0.945	1.012	0.955	0.997
P06733	Alpha-enolase	ENO1	39.63	15	15	38	434	47.1	7.39	1.143	1.012	1.155	0.997
P17181	Interferon alpha/beta receptor 1	IFNAR1	2.87	2	2	4	557	63.5	5.81	0.857	1.013	0.867	0.998
P09668	Pro-cathepsin H	CTSH	18.21	6	6	23	335	37.4	8.07	1.326	1.013	1.341	0.998
P07998	Ribonuclease pancreatic	RNASE1	55.77	5	5	195	156	17.6	8.79	1.121	1.014	1.133	0.999
Q9GZX9	Twisted gastrulation protein homolog 1	TWSG1	24.22	4	4	17	223	25.0	5.34	1.185	1.015	1.197	1.000
P12830	Cadherin-1	CDH1	14.63	8	9	35	882	97.4	4.73	1.381	1.016	1.396	1.000
Q8IV08	Phospholipase D3	PLD3	11.02	4	4	7	490	54.7	6.47	0.976	1.016	0.986	1.001
Q9UBQ7	Glyoxylate reductase/hydroxypyruvate reductase	GRHPR	10.67	2	2	2	328	35.6	7.39	0.879	1.016	0.889	1.001
Q03403	Trefoil factor 2	TFF2	40.31	4	4	149	129	14.3	5.81	1.106	1.017	1.118	1.002
P02649	Apolipoprotein E	APOE	42.90	13	13	34	317	36.1	5.73	1.435	1.018	1.450	1.002
P62491	Ras-related protein Rab-11A	RAB11A	8.33	2	2	2	216	24.4	6.57	1.043	1.018	1.054	1.003
Q15286	Ras-related protein Rab-35	RAB35	9.95	2	2	4	201	23.0	8.29	1.026	1.020	1.037	1.004
Q96EY5	Multivesicular body subunit 12A	MVB12A	22.71	4	4	5	273	28.8	8.91	1.280	1.021	1.294	1.005
P06454	Prothymosin alpha	PTMA	34.23	4	4	15	111	12.2	3.78	0.991	1.022	1.002	1.006
P60981	Destrin	DSTN	17.58	3	3	3	165	18.5	7.85	1.055	1.022	1.067	1.007
P0CG48	Polyubiquitin-C	UBC	89.49	8	8	150	685	77.0	7.66	1.405	1.023	1.420	1.008
P02749	Beta-2-glycoprotein 1	APOH	49.86	14	14	158	345	38.3	7.97	1.541	1.023	1.558	1.008
P07288	Prostate-specific antigen	KLK3	40.23	8	8	87	261	28.7	7.68	0.444	1.023	0.449	1.008
P08754	Guanine nucleotide-binding protein G(i) subunit alpha	GNAI3	12.99	4	4	4	354	40.5	5.69	0.851	1.024	0.860	1.009
P54826	Growth arrest-specific protein 1	GAS1	14.20	2	2	10	345	35.7	5.55	0.962	1.025	0.972	1.010
P12273	Prolactin-inducible protein	PIP	63.70	8	8	112	146	16.6	8.05	0.144	1.026	0.146	1.010
Q8IZF2	Adhesion G protein-coupled receptor F5	ADGRF5	5.42	6	6	16	1346	149.4	6.65	1.141	1.027	1.153	1.011
P06858	Lipoprotein lipase	LPL	6.32	2	2	4	475	53.1	8.15	0.392	1.029	0.396	1.013
P19320	Vascular cell adhesion protein 1	VCAM1	19.49	11	11	23	739	81.2	5.22	2.173	1.029	2.196	1.013
Q96KP4	Cytosolic non-specific dipeptidase	CNDP2	16.42	5	5	15	475	52.8	5.97	0.936	1.030	0.946	1.014
O95967	EGF-containing fibulin-like extracellular matrix protein 2	EFEMP2	13.09	5	5	9	443	49.4	4.94	1.309	1.032	1.323	1.017
Q14108	Lysosome membrane protein 2	SCARB2	8.79	2	2	6	478	54.3	5.14	1.136	1.036	1.148	1.020
O00560	Syntenin-1	SDCBP	31.54	5	5	15	298	32.4	7.53	0.976	1.036	0.986	1.021
P15309	Prostatic acid phosphatase	ACPP	36.01	13	13	209	386	44.5	6.24	0.396	1.037	0.400	1.021
O95497	Pantetheinase	VNN1	24.95	7	7	13	513	57.0	5.55	0.751	1.037	0.759	1.022
P30085	UMP-CMP kinase	CMPK1	35.71	5	5	8	196	22.2	5.57	0.960	1.038	0.971	1.022
P35858	Insulin-like growth factor-binding protein complex acid labile subunit	IGFALS	12.73	5	5	13	605	66.0	6.79	0.887	1.038	0.896	1.023
O94772	Lymphocyte antigen 6H	LY6H	28.57	3	3	6	140	14.7	7.25	1.237	1.040	1.250	1.024
Q96PC5	Melanoma inhibitory activity protein 2	MIA2	1.49	2	2	4	1412	159.7	4.69	0.613	1.041	0.620	1.025
P08195	4F2 cell-surface antigen heavy chain	SLC3A2	18.25	9	9	16	630	68.0	5.01	0.910	1.043	0.920	1.027
O75309	Cadherin-16	CDH16	13.15	7	7	16	829	89.9	4.96	1.341	1.045	1.355	1.029
Q13591	Semaphorin-5A	SEMA5A	1.77	2	2	6	1074	120.5	7.21	1.182	1.045	1.195	1.029
O15230	Laminin subunit alpha-5	LAMA5	2.65	7	7	9	3695	399.5	7.02	0.923	1.046	0.933	1.030
P04180	Phosphatidylcholine-sterol acyltransferase	LCAT	24.55	7	7	18	440	49.5	6.11	0.867	1.046	0.876	1.030
P08697	Alpha-2-antiplasmin	SERPINF2	29.74	9	9	16	491	54.5	6.29	1.227	1.047	1.240	1.031
Q9Y5F6	Protocadherin gamma-C5	PCDHGC5	4.24	2	2	2	944	101.9	5.03	0.651	1.047	0.658	1.031
P07741	Adenine phosphoribosyltransferase	APRT	10.56	2	2	2	180	19.6	6.02	1.242	1.048	1.255	1.032
Q9Y5K6	CD2-associated protein	CD2AP	5.79	3	3	3	639	71.4	6.40	0.783	1.049	0.791	1.033
Q9UNW1	Multiple inositol polyphosphate phosphatase 1	MINPP1	5.34	2	2	2	487	55.0	7.81	0.848	1.050	0.857	1.034
Q9UN37	Vacuolar protein sorting-associated protein 4A	VPS4A	18.54	5	8	16	437	48.9	7.80	1.049	1.051	1.061	1.035
P07602	Prosaposin	PSAP	30.73	15	15	322	524	58.1	5.17	1.021	1.051	1.032	1.035
Q12841	Follistatin-related protein 1	FSTL1	25.00	7	7	17	308	35.0	5.52	0.579	1.052	0.586	1.036
P61769	Beta-2-microglobulin	B2M	54.62	5	5	73	119	13.7	6.52	0.730	1.052	0.738	1.036
P24821	Tenascin	TNC	11.09	18	18	32	2201	240.7	4.89	0.999	1.053	1.010	1.037
Q15828	Cystatin-M	CST6	50.34	5	5	85	149	16.5	8.09	1.963	1.053	1.984	1.037
P14543	Nidogen-1	NID1	15.80	12	12	47	1247	136.3	5.29	0.761	1.053	0.769	1.037
P08253	72 kDa type IV collagenase	MMP2	6.67	3	4	9	660	73.8	5.47	1.013	1.054	1.024	1.038
P51148	Ras-related protein Rab-5C	RAB5C	22.69	4	4	7	216	23.5	8.41	0.587	1.055	0.594	1.039
P08133	Annexin A6	ANXA6	4.75	2	2	2	673	75.8	5.60	1.230	1.056	1.243	1.041
P62258	14-3-3 protein epsilon	YWHAE	54.51	11	12	32	255	29.2	4.74	0.687	1.057	0.694	1.041
P07686	Beta-hexosaminidase subunit beta	HEXB	25.00	11	11	26	556	63.1	6.76	0.934	1.058	0.944	1.042
Q969X1	Protein lifeguard 3	TMBIM1	4.82	2	2	5	311	34.6	7.72	0.765	1.058	0.773	1.042
P06396	Gelsolin	GSN	32.61	23	23	89	782	85.6	6.28	1.535	1.060	1.551	1.044
P31146	Coronin-1A	CORO1A	5.86	2	2	3	461	51.0	6.68	1.735	1.062	1.754	1.046
Q5ZPR3	CD276 antigen	CD276	14.98	3	3	7	534	57.2	4.91	1.146	1.062	1.158	1.046
P17050	Alpha-N-acetylgalactosaminidase	NAGA	14.60	4	4	9	411	46.5	5.19	1.420	1.062	1.435	1.046
Q8N6Q3	CD177 antigen	CD177	7.32	3	3	7	437	46.3	6.29	0.522	1.063	0.527	1.047
Q8NFZ8	Cell adhesion molecule 4	CADM4	20.36	6	6	24	388	42.8	6.30	0.902	1.063	0.912	1.047
P15848	Arylsulfatase B	ARSB	9.76	4	4	7	533	59.6	8.21	1.598	1.064	1.615	1.048
Q92626	Peroxidasin homolog	PXDN	2.70	3	3	4	1479	165.2	7.17	1.202	1.065	1.215	1.049
P33908	Mannosyl-oligosaccharide 1,2-alpha-mannosidase IA	MAN1A1	28.18	16	16	34	653	72.9	6.47	0.971	1.065	0.981	1.049
Q96DG6	Carboxymethylenebutenolidase homolog	CMBL	36.33	8	8	12	245	28.0	7.18	1.108	1.065	1.120	1.049
P55291	Cadherin-15	CDH15	12.16	6	6	9	814	88.9	4.98	0.408	1.066	0.413	1.050
Q08345	Epithelial discoidin domain-containing receptor 1	DDR1	8.98	6	6	12	913	101.1	6.83	0.997	1.066	1.007	1.050
P98172	Ephrin-B1	EFNB1	13.01	4	4	17	346	38.0	8.94	1.562	1.066	1.578	1.050

O75874	Isocitrate dehydrogenase [NADP] cytoplasmic	IDH1	28.99	12	12	28	414	46.6	7.01	0.415	1.067	0.419	1.051
P35241	Radixin	RDX	18.18	4	11	16	583	68.5	6.37	1.293	1.067	1.307	1.051
Q6P531	Glutathione hydrolase 6	GGT6	6.49	2	2	3	493	50.5	6.07	0.880	1.068	0.890	1.052
P0C0L4	Complement C4-A	C4A	38.36	2	48	142	1744	192.7	7.08	2.857	1.070	2.888	1.054
P78371	T-complex protein 1 subunit beta	CCT2	4.30	2	2	2	535	57.5	6.46	0.970	1.071	0.981	1.055
P63208	S-phase kinase-associated protein 1	SKP1	13.50	2	2	4	163	18.6	4.54	1.001	1.072	1.012	1.056
Q9UGT4	Sushi domain-containing protein 2	SUSD2	14.48	8	8	14	822	90.1	6.28	0.710	1.073	0.718	1.057
P08236	Beta-glucuronidase	GUSB	21.51	9	9	23	651	74.7	7.02	0.997	1.074	1.008	1.058
P12109	Collagen alpha-1(VI) chain	COL6A1	41.73	29	29	135	1028	108.5	5.43	1.040	1.075	1.051	1.058
Q8N5I2	Arrestin domain-containing protein 1	ARRDC1	7.39	2	2	4	433	46.0	7.02	1.142	1.075	1.154	1.059
Q9UBG0	C-type mannose receptor 2	MRC2	1.89	2	2	2	1479	166.6	5.83	1.132	1.076	1.144	1.060
Q8TCT8	Signal peptide peptidase-like 2A	SPPL2A	4.04	2	2	3	520	58.1	8.32	0.925	1.078	0.935	1.062
P80723	Brain acid soluble protein 1	BASP1	69.60	9	9	16	227	22.7	4.63	0.428	1.079	0.432	1.063
P63092	Guanine nucleotide-binding protein G(s) subunit alpha isoforms short	GNAS	17.51	6	6	9	394	45.6	5.82	0.730	1.080	0.738	1.063
P38606	V-type proton ATPase catalytic subunit A	ATP6V1A	10.21	4	4	6	617	68.3	5.52	0.841	1.080	0.850	1.063
Q9HCN6	Platelet glycoprotein VI	GP6	15.04	4	4	17	339	36.8	9.20	0.775	1.081	0.783	1.064
Q9BYJ0	Fibroblast growth factor-binding protein 2	FGFBP2	13.90	2	2	5	223	24.6	8.87	0.931	1.081	0.941	1.065
P54793	Arylsulfatase F	ARSF	8.64	3	3	3	590	65.9	7.21	0.958	1.083	0.968	1.067
P54710	Sodium/potassium-transporting ATPase subunit gamma	FXYD2	39.39	2	2	3	66	7.3	8.16	0.417	1.087	0.422	1.070
Q9UKK9	ADP-sugar pyrophosphatase	NUDT5	11.42	2	2	2	219	24.3	4.94	0.685	1.089	0.692	1.072
Q9NZU0	Leucine-rich repeat transmembrane protein FLRT3	FLRT3	4.01	2	2	2	649	73.0	7.64	1.786	1.090	1.805	1.073
P17174	Aspartate aminotransferase, cytoplasmic	GOT1	12.35	4	4	7	413	46.2	7.01	0.830	1.091	0.839	1.075
Q8N307	Mucin-20	MUC20	29.90	4	4	7	709	71.9	5.07	0.346	1.091	0.350	1.075
P07711	Cathepsin L1	CTSL	29.73	7	7	15	333	37.5	5.45	1.235	1.092	1.248	1.075
P22223	Cadherin-3	CDH3	4.10	2	3	7	829	91.4	4.75	1.267	1.093	1.281	1.076
Q9H9H4	Vacuolar protein sorting-associated protein 37B	VPS37B	25.96	4	4	5	285	31.3	7.34	1.177	1.093	1.189	1.077
P17931	Galectin-3	LGALS3	28.40	6	6	15	250	26.1	8.56	1.053	1.094	1.064	1.077
P47756	F-actin-capping protein subunit beta	CAPZB	13.36	3	3	5	277	31.3	5.59	1.950	1.094	1.971	1.077
Q9HC38	Glyoxalase domain-containing protein 4	GLOD4	16.61	5	5	9	313	34.8	5.60	0.877	1.094	0.886	1.078
Q7Z4W1	L-xylulose reductase	DCXR	18.44	3	3	3	244	25.9	8.10	0.713	1.097	0.721	1.080
P07900	Heat shock protein HSP 90-alpha	HSP90AA1	19.67	7	11	16	732	84.6	5.02	0.596	1.097	0.602	1.080
Q9H3G5	Probable serine carboxypeptidase CPVL	CPVL	30.88	11	11	34	476	54.1	5.62	1.167	1.097	1.179	1.080
P54802	Alpha-N-acetylglucosaminidase	NAGLU	35.94	18	18	83	743	82.2	6.65	1.173	1.098	1.185	1.081
P21333	Filamin-A	FLNA	8.05	15	15	23	2647	280.6	6.06	1.040	1.100	1.051	1.083
Q68D85	Natural cytotoxicity triggering receptor 3 ligand 1	NCR3LG1	12.33	4	4	7	454	50.8	5.59	0.520	1.100	0.525	1.084
P62820	Ras-related protein Rab-1A	RAB1A	13.17	3	3	5	205	22.7	6.21	0.655	1.100	0.662	1.084
Q96FN5	Kinesin-like protein KIF12	KIF12	5.73	3	3	6	646	70.6	8.85	1.297	1.101	1.311	1.084
P78552	Interleukin-13 receptor subunit alpha-1	IL13RA1	6.32	2	2	3	427	48.7	6.01	1.282	1.103	1.296	1.086
P13647	Keratin, type II cytoskeletal 5	KRT5	27.97	9	19	39	590	62.3	7.74	1.712	1.104	1.730	1.087
P49221	Protein-glutamine gamma-glutamyltransferase 4	TGM4	11.11	6	6	13	684	77.1	6.76	0.235	1.104	0.237	1.088
Q07654	Trefoil factor 3	TFF3	21.28	3	3	5	94	10.2	6.92	1.949	1.105	1.970	1.088
O43866	CD5 antigen-like	CD5L	12.39	3	3	3	347	38.1	5.47	1.059	1.107	1.071	1.090
Q9Y376	Calcium-binding protein 39	CAB39	12.61	4	4	8	341	39.8	6.89	1.306	1.109	1.320	1.092
P11142	Heat shock cognate 71 kDa protein	HSPA8	30.65	14	18	37	646	70.9	5.52	1.114	1.109	1.126	1.093
P62993	Growth factor receptor-bound protein 2	GRB2	11.98	2	2	2	217	25.2	6.32	1.328	1.110	1.342	1.093
O60635	Tetraspanin-1	TSPAN1	8.71	2	2	9	241	26.3	5.25	1.407	1.110	1.422	1.093
O15031	Plexin-B2	PLXNB2	1.85	3	3	3	1838	205.0	6.24	1.181	1.111	1.193	1.094
Q6UY11	Protein delta homolog 2	DLK2	13.32	4	4	11	383	40.5	6.54	0.710	1.111	0.718	1.095
P09923	Intestinal-type alkaline phosphatase	ALPI	3.98	2	2	2	528	56.8	5.86	1.875	1.112	1.895	1.095
P15941	Mucin-1	MUC1	6.14	6	6	29	1255	122.0	7.47	1.143	1.113	1.155	1.096
Q9UBX5	Fibulin-5	FBLN5	17.63	7	7	17	448	50.1	4.73	1.188	1.115	1.201	1.098
Q9UL25	Ras-related protein Rab-21	RAB21	8.89	2	2	2	225	24.3	7.94	1.737	1.116	1.756	1.099
Q02383	Semenogelin-2	SEMG2	66.84	26	30	352	582	65.4	9.07	0.169	1.116	0.171	1.100
P50995	Annexin A11	ANXA11	26.53	11	11	30	505	54.4	7.65	0.739	1.117	0.747	1.100
Q92954	Proteoglycan 4	PRG4	4.34	7	7	13	1404	151.0	9.51	1.419	1.117	1.435	1.101
Q12805	EGF-containing fibulin-like extracellular matrix protein 1	EFEMP1	42.19	16	16	69	493	54.6	5.07	1.189	1.118	1.202	1.101
A0M8Q6	Immunoglobulin lambda constant 7	IGLC7	60.38	2	6	76	106	11.2	8.29	2.261	1.118	2.285	1.101
Q07837	Neutral and basic amino acid transport protein rBAT	SLC3A1	8.76	5	5	7	685	78.8	5.96	0.858	1.119	0.867	1.102
P27487	Dipeptidyl peptidase 4	DPP4	25.20	17	17	59	766	88.2	6.04	1.001	1.124	1.012	1.107
P01859	Immunoglobulin heavy constant gamma 2	IGHG2	38.96	4	12	320	326	35.9	7.59	1.789	1.126	1.808	1.109
Q9NY33	Dipeptidyl peptidase 3	DPP3	4.88	2	2	2	737	82.5	5.10	1.246	1.129	1.259	1.112
P35908	Keratin, type II cytoskeletal 2 epidermal	KRT2	43.51	18	24	52	639	65.4	8.00	2.364	1.129	2.390	1.112
P07948	Tyrosine-protein kinase Lyn	LYN	3.71	2	2	2	512	58.5	7.11	1.157	1.132	1.170	1.115
P04066	Tissue alpha-L-fucosidase	FUCA1	5.36	2	2	7	466	53.7	6.84	1.083	1.132	1.094	1.115
P15586	N-acetylglucosamine-6-sulfatase	GNS	29.17	14	14	67	552	62.0	8.31	1.389	1.134	1.404	1.117
O75354	Ectonucleoside triphosphate diphosphohydrolase 6	ENTPD6	5.37	2	2	2	484	53.2	9.26	0.726	1.135	0.734	1.118
P68104	Elongation factor 1-alpha 1	EEF1A1	19.26	8	8	19	462	50.1	9.01	0.699	1.136	0.707	1.118
Q9UQ52	Contactin-6	CNTN6	3.21	2	2	2	1028	113.9	6.00	1.558	1.136	1.575	1.118
P10643	Complement component C7	C7	13.88	11	11	35	843	93.5	6.48	1.064	1.138	1.076	1.120
P29622	Kallistatin	SERPINA4	40.05	12	12	21	427	48.5	7.75	1.244	1.138	1.257	1.121
Q14624	Inter-alpha-trypsin inhibitor heavy chain H4	ITIH4	32.58	21	21	436	930	103.3	6.98	1.405	1.139	1.420	1.122
P50990	T-complex protein 1 subunit theta	CCT8	3.65	2	2	2	548	59.6	5.60	0.749	1.139	0.757	1.122
P53634	Dipeptidyl peptidase 1	CTSC	15.55	5	5	19	463	51.8	6.99	1.157	1.140	1.169	1.123
O00241	Signal-regulatory protein beta-1	SIRPB1	38.69	9	13	42	398	43.2	6.52	1.032	1.140	1.043	1.123
Q16651	Prostasin	PRSS8	11.66	3	3	25	343	36.4	5.85	1.103	1.140	1.115	1.123
Q9UHL4	Dipeptidyl peptidase 2	DPP7	33.33	11	11	24	492	54.3	6.32	1.062	1.141	1.073	1.123
P04279	Semenogelin-1	SEMG1	60.61	23	27	651	462	52.1	9.29	0.113	1.143	0.115	1.126
P08758	Annexin A5	ANXA5	30.00	9	9	13	320	35.9	5.05	1.103	1.144	1.115	1.127
P25311	Zinc-alpha-2-glycoprotein	AZGP1	53.36	17	17	396	298	34.2	6.05	1.037	1.149	1.048	1.131
P61224	Ras-related protein Rap-1b	RAP1B	29.89	5	5	11	184	20.8	5.78	1.017	1.149	1.027	1.132
P60174	Triosephosphate isomerase	TPI1	38.11	9	9	34	286	30.8	5.92	1.166	1.150	1.178	1.132
P55268	Laminin subunit beta-2	LAMB2	1.11	2	2	2	1798	195.9	6.52	0.806	1.150	0.814	1.133
Q92597	Protein NDRG1	NDRG1	8.38	2	2	3	394	42.8	5.82	0.453	1.150	0.458	1.133
Q99988	Growth/differentiation factor 15	GDF15	13.31	3	3	6	308	34.1	9.66	1.211	1.151	1.224	1.134
O14773	Tripeptidyl-peptidase 1	TPP1	16.16	6	6	21	563	61.2	6.48	1.204	1.155	1.217	1.137
P46108	Adapter molecule crk	CRK	10.86	2	2	2	304	33.8	5.55	0.939	1.163	0.949	1.145
Q86VP6	Cullin-associated NEDD8-dissociated protein 1	CAND1	2.60	2	2	2	1230	136.3	5.78	0.482	1.163	0.487	1.146
O60704	Protein-tyrosine sulfotransferase 2	TPST2	7.96	2	2	2	377	41.9	9.09	1.004	1.163	1.014	1.146
Q9HAT2	Sialate O-acetylesterase	SIAE	33.08	15	15	34	523	58.3	7.33	1.334	1.164	1.349	1.146

P12277	Creatine kinase B-type	CKB	22.83	5	5	10	381	42.6	5.59	0.570	1.164	0.576	1.147
P13489	Ribonuclease inhibitor	RNH1	8.03	2	2	2	461	49.9	4.82	0.777	1.165	0.785	1.147
P41271	Neuroblastoma suppressor of tumorigenicity 1	NBL1	7.73	2	2	2	181	19.4	5.29	0.512	1.165	0.517	1.147
P26927	Hepatocyte growth factor-like protein	MST1	9.28	4	4	5	711	80.3	7.68	1.313	1.167	1.327	1.150
O00115	Deoxyribonuclease-2-alpha	DNASE2	7.22	2	2	4	360	39.6	8.05	1.686	1.168	1.704	1.151
Q9BXP8	Pappalysin-2	PAPPA2	8.43	13	13	22	1791	198.4	5.47	1.265	1.169	1.278	1.151
Q9UM22	Mammalian ependymin-related protein 1	EPDR1	23.66	5	5	11	224	25.4	6.60	1.744	1.175	1.763	1.157
Q9HBR0	Putative sodium-coupled neutral amino acid transporter 10	SLC38A10	6.34	5	5	7	1119	119.7	5.73	0.752	1.176	0.760	1.159
P25788	Proteasome subunit alpha type-3	PSMA3	9.41	2	2	2	255	28.4	5.33	1.085	1.177	1.097	1.159
P07437	Tubulin beta chain	TUBB	8.56	2	2	2	444	49.6	4.89	0.693	1.180	0.700	1.162
P13798	Acylamino-acid-releasing enzyme	APEH	4.51	3	3	5	732	81.2	5.48	0.742	1.182	0.750	1.164
Q13510	Acid ceramidase	ASAH1	40.25	14	14	35	395	44.6	7.62	1.150	1.184	1.162	1.166
P23528	Cofilin-1	CFL1	57.23	9	9	15	166	18.5	8.09	0.822	1.185	0.831	1.167
Q15847	Adipogenesis regulatory factor	ADIRF	19.74	2	2	2	76	7.8	5.31	0.621	1.185	0.628	1.168
Q9UKU6	Thyrotropin-releasing hormone-degrading ectoenzyme	TRHDE	5.08	4	4	7	1024	116.9	6.99	0.914	1.187	0.924	1.169
P13861	cAMP-dependent protein kinase type II-alpha regulatory subunit	PRKAR2A	6.44	2	2	2	404	45.5	5.07	0.851	1.188	0.860	1.170
P34059	N-acetylgalactosamine-6-sulfatase	GALNS	13.03	5	6	13	522	58.0	6.74	1.393	1.189	1.408	1.171
Q9HD89	Resistin	RETN	42.59	4	4	10	108	11.4	6.86	0.919	1.189	0.929	1.172
Q3L8U1	Chromodomain-helicase-DNA-binding protein 9	CHD9	0.69	2	2	2	2897	325.8	7.01	0.894	1.190	0.904	1.172
P17900	Ganglioside GM2 activator	GM2A	33.16	6	6	52	193	20.8	5.31	1.804	1.191	1.823	1.173
O75131	Copine-3	CPNE3	16.01	8	8	12	537	60.1	5.85	0.983	1.191	0.993	1.173
P17936	Insulin-like growth factor-binding protein 3	IGFBP3	19.24	5	5	14	291	31.7	8.69	0.883	1.191	0.892	1.174
P00746	Complement factor D	CFD	22.13	4	4	7	253	27.0	7.71	1.524	1.192	1.541	1.174
O75503	Ceroid-lipofuscinosis neuronal protein 5	CLN5	13.41	4	4	10	358	41.5	7.40	0.858	1.196	0.867	1.178
P02760	Protein AMBP	AMBP	60.51	21	21	1294	352	39.0	6.25	1.630	1.197	1.647	1.178
P10721	Mast/stem cell growth factor receptor Kit	KIT	1.95	2	2	2	976	109.8	6.98	1.033	1.197	1.044	1.179
P02750	Leucine-rich alpha-2-glycoprotein	LRG1	44.96	11	11	71	347	38.2	6.95	2.790	1.197	2.820	1.179
P04899	Guanine nucleotide-binding protein G(i) subunit alpha-2	GNAI2	15.49	4	4	4	355	40.4	5.54	1.118	1.203	1.130	1.184
P30626	Sorcin	SRI	16.16	2	2	4	198	21.7	5.59	1.009	1.203	1.020	1.185
P18510	Interleukin-1 receptor antagonist protein	IL1RN	29.94	3	3	10	177	20.0	6.19	1.179	1.205	1.191	1.187
O95164	Ubiquitin-like protein 3	UBL3	16.24	2	2	2	117	13.1	6.77	1.458	1.205	1.473	1.187
P61981	14-3-3 protein gamma	YWHAG	31.58	5	8	24	247	28.3	4.89	0.786	1.207	0.794	1.189
P02533	Keratin, type I cytoskeletal 14	KRT14	17.16	4	6	10	472	51.5	5.16	2.264	1.208	2.288	1.190
Q9Y5Z4	Heme-binding protein 2	HEBP2	22.93	4	4	5	205	22.9	4.63	0.952	1.208	0.962	1.190
P07737	Profilin-1	PFN1	50.00	6	6	13	140	15.0	8.27	2.196	1.208	2.219	1.190
P0DOY2	Immunoglobulin lambda constant 2	IGLC2	96.23	4	10	415	106	11.3	7.24	2.096	1.211	2.118	1.192
Q96IU4	Protein ABHD14B	ABHD14B	39.52	6	6	17	210	22.3	6.40	1.249	1.213	1.262	1.194
P51149	Ras-related protein Rab-7a	RAB7A	17.87	3	3	6	207	23.5	6.70	1.073	1.214	1.084	1.195
O95274	Ly6/PLAUR domain-containing protein 3	LYPD3	17.92	6	6	16	346	35.9	7.75	1.218	1.214	1.231	1.195
Q9UBR2	Cathepsin Z	CTSZ	9.24	3	3	6	303	33.8	7.11	0.911	1.214	0.921	1.196
P30101	Protein disulfide-isomerase A3	PDIA3	6.73	3	3	5	505	56.7	6.35	0.818	1.214	0.827	1.196
Q96NY8	Nectin-4	NECTIN4	21.18	8	8	25	510	55.4	5.38	0.932	1.215	0.942	1.197
P19021	Peptidyl-glycine alpha-amidating monooxygenase	PAM	8.12	7	7	10	973	108.3	6.42	1.239	1.217	1.253	1.199
Q14508	WAP four-disulfide core domain protein 2	WFDC2	32.26	3	3	31	124	13.0	4.84	1.025	1.220	1.036	1.202
P07108	Acyl-CoA-binding protein	DBI	58.62	4	4	10	87	10.0	6.57	0.920	1.223	0.930	1.204
P07996	Thrombospondin-1	THBS1	13.85	13	13	27	1170	129.3	4.94	0.841	1.224	0.850	1.205
P27797	Calreticulin	CALR	18.47	7	7	15	417	48.1	4.44	0.833	1.225	0.842	1.206
P15311	Ezrin	EZR	34.47	14	20	40	586	69.4	6.27	1.127	1.225	1.139	1.206
Q9BZR6	Reticulon-4 receptor	RTN4R	4.86	2	2	2	473	50.7	8.87	0.957	1.226	0.967	1.208
P01871	Immunoglobulin heavy constant mu	IGHM	30.91	13	13	185	453	49.4	6.77	2.627	1.227	2.655	1.209
Q9BRF8	Serine/threonine-protein phosphatase CPPED1	CPPED1	8.92	2	2	3	314	35.5	6.20	2.888	1.229	2.919	1.210
P04070	Vitamin K-dependent protein C	PROC	5.42	2	2	2	461	52.0	6.28	0.746	1.229	0.754	1.211
P04062	Lysosomal acid glucosylceramidase	GBA	6.53	3	3	3	536	59.7	7.61	1.019	1.230	1.029	1.211
P09211	Glutathione S-transferase P	GSTP1	46.67	7	7	15	210	23.3	5.64	1.432	1.232	1.447	1.214
Q14914	Prostaglandin reductase 1	PTGR1	27.36	7	7	10	329	35.8	8.29	0.893	1.233	0.903	1.214
P07358	Complement component C8 beta chain	C8B	4.23	2	2	3	591	67.0	8.13	1.049	1.233	1.060	1.215
Q16706	Alpha-mannosidase 2	MAN2A1	1.84	2	2	2	1144	131.1	7.58	1.215	1.236	1.228	1.217
Q15181	Inorganic pyrophosphatase	PPA1	10.73	3	3	5	289	32.6	5.86	1.157	1.236	1.169	1.217
P61088	Ubiquitin-conjugating enzyme E2 N	UBE2N	13.82	2	2	6	152	17.1	6.57	1.056	1.239	1.067	1.220
P04406	Glyceraldehyde-3-phosphate dehydrogenase	GAPDH	38.51	10	10	25	335	36.0	8.46	0.970	1.239	0.980	1.221
O94919	Endonuclease domain-containing 1 protein	ENDOD1	18.40	8	8	48	500	55.0	5.71	1.471	1.240	1.487	1.221
O15484	Calpain-5	CAPN5	6.88	3	3	3	640	73.1	7.64	1.289	1.240	1.302	1.222
Q9Y2E5	Epididymis-specific alpha-mannosidase	MAN2B2	13.38	10	10	20	1009	113.9	7.24	1.072	1.240	1.083	1.222
Q9BYE9	Cadherin-related family member 2	CDHR2	10.92	11	11	26	1310	141.5	4.50	0.979	1.240	0.990	1.222
O00391	Sulfhydryl oxidase 1	QSOX1	35.74	21	21	66	747	82.5	8.92	1.155	1.244	1.167	1.225
P07204	Thrombomodulin	THBD	11.13	3	3	4	575	60.3	4.92	0.269	1.245	0.271	1.226
P01833	Polymeric immunoglobulin receptor	PIGR	42.93	27	27	468	764	83.2	5.74	1.271	1.245	1.285	1.227
P31946	14-3-3 protein beta/alpha	YWHAB	24.39	2	7	23	246	28.1	4.83	1.004	1.249	1.014	1.230
P04004	Vitronectin	VTN	16.32	7	7	110	478	54.3	5.80	1.415	1.250	1.430	1.231
P26038	Moesin	MSN	35.18	16	23	40	577	67.8	6.40	1.343	1.252	1.357	1.233
Q8WVQ1	Soluble calcium-activated nucleotidase 1	CANT1	15.21	4	4	7	401	44.8	6.09	0.930	1.253	0.940	1.234
Q9UHG2	ProSAAS	PCSK1N	26.54	4	4	10	260	27.4	6.62	0.630	1.254	0.636	1.235
O75594	Peptidoglycan recognition protein 1	PGLYRP1	57.14	7	7	77	196	21.7	8.59	1.457	1.254	1.473	1.235
Q99969	Retinoic acid receptor responder protein 2	RARRES2	21.47	3	3	6	163	18.6	9.09	0.986	1.257	0.997	1.238
P00338	L-lactate dehydrogenase A chain	LDHA	25.30	7	8	11	332	36.7	8.27	0.946	1.258	0.956	1.239
P04792	Heat shock protein beta-1	HSPB1	27.32	6	6	24	205	22.8	6.40	0.951	1.259	0.961	1.240
P27348	14-3-3 protein theta	YWHAQ	20.00	2	6	17	245	27.7	4.78	0.782	1.259	0.791	1.240
O00754	Lysosomal alpha-mannosidase	MAN2B1	2.57	2	2	3	1011	113.7	7.28	1.383	1.260	1.398	1.241
O75752	UDP-GalNAc:beta-1,3-N-acetylgalactosaminyltransferase 1	B3GALNT1	10.27	2	2	2	331	39.5	7.80	0.738	1.261	0.746	1.242
Q9BQE3	Tubulin alpha-1C chain	TUBA1C	9.13	3	3	6	449	49.9	5.10	0.717	1.264	0.725	1.245
O00264	Membrane-associated progesterone receptor component 1	PGRMC1	14.36	2	2	2	195	21.7	4.70	0.816	1.265	0.824	1.245
P07858	Cathepsin B	CTSB	30.68	8	8	56	339	37.8	6.30	1.254	1.267	1.267	1.248
P15428	15-hydroxyprostaglandin dehydrogenase [NAD(+)]	HPGD	9.77	2	2	2	266	29.0	5.86	0.282	1.268	0.285	1.249
P13667	Protein disulfide-isomerase A4	PDIA4	8.22	5	5	8	645	72.9	5.07	0.946	1.269	0.956	1.250
P17858	ATP-dependent 6-phosphofructokinase, liver type	PFKL	6.67	3	3	3	780	85.0	7.50	1.177	1.270	1.190	1.250
Q9NR34	Mannosyl-oligosaccharide 1,2-alpha-mannosidase IC	MAN1C1	6.35	3	3	5	630	70.9	7.46	0.641	1.271	0.648	1.252
Q14697	Neutral alpha-glucosidase AB	GANAB	5.61	3	3	4	944	106.8	6.14	0.621	1.273	0.627	1.254
P01624	Immunoglobulin kappa variable 3-15	IGKV3-15	26.09	2	2	6	115	12.5	5.19	1.686	1.274	1.704	1.255
P00749	Urokinase-type plasminogen activator	PLAU	38.28	14	14	52	431	48.5	8.41	0.856	1.275	0.865	1.255

O75340	Programmed cell death protein 6	PDCD6	16.75	3	3	5	191	21.9	5.40	0.873	1.277	0.883	1.258
P15144	Aminopeptidase N	ANPEP	34.44	34	34	146	967	109.5	5.48	0.943	1.277	0.953	1.258
O75083	WD repeat-containing protein 1	WDR1	11.55	6	6	12	606	66.2	6.65	0.830	1.277	0.839	1.258
Q9NP84	Tumor necrosis factor receptor superfamily member 12A	TNFRSF12A	13.95	2	2	2	129	13.9	8.95	1.466	1.277	1.482	1.258
Q99584	Protein S100-A13	S100A13	30.61	3	3	8	98	11.5	6.16	1.096	1.280	1.108	1.261
Q8NCC3	Group XV phospholipase A2	PLA2G15	23.79	8	8	12	412	46.6	6.73	1.919	1.281	1.939	1.262
P29992	Guanine nucleotide-binding protein subunit alpha-11	GNA11	9.19	3	3	6	359	42.1	5.69	1.044	1.281	1.055	1.262
O75223	Gamma-glutamylcyclotransferase	GGCT	9.04	2	2	3	188	21.0	5.14	1.076	1.282	1.087	1.262
Q14956	Transmembrane glycoprotein NMB	GPNMB	4.37	2	2	2	572	63.9	6.64	1.265	1.284	1.278	1.264
Q13404	Ubiquitin-conjugating enzyme E2 variant 1	UBE2V1	9.52	2	2	2	147	16.5	7.93	0.983	1.288	0.993	1.268
P10909	Clusterin	CLU	34.52	15	15	146	449	52.5	6.27	0.764	1.289	0.773	1.270
Q12907	Vesicular integral-membrane protein VIP36	LMAN2	55.34	14	14	234	356	40.2	6.95	1.452	1.290	1.468	1.270
P15289	Arylsulfatase A	ARSA	27.61	10	11	48	507	53.6	6.07	1.217	1.291	1.230	1.271
P19801	Amiloride-sensitive amine oxidase [copper-containing]	AOC1	5.33	4	4	9	751	85.3	7.09	0.808	1.292	0.816	1.273
P54803	Galactocerebrosidase	GALC	4.67	2	2	4	685	77.0	6.64	1.165	1.295	1.178	1.275
Q92484	Acid sphingomyelinase-like phosphodiesterase 3a	SMPDL3A	3.75	2	2	4	453	51.2	6.33	1.513	1.295	1.530	1.276
Q92485	Acid sphingomyelinase-like phosphodiesterase 3b	SMPDL3B	11.87	4	4	5	455	50.8	5.64	1.439	1.299	1.455	1.279
Q9GZM7	Tubulointerstitial nephritis antigen-like	TINAGL1	8.78	2	2	3	467	52.4	6.99	0.605	1.299	0.611	1.279
P04746	Pancreatic alpha-amylase	AMY2A	48.14	4	17	427	511	57.7	7.05	1.042	1.299	1.053	1.279
Q08380	Galectin-3-binding protein	LGALS3BP	38.29	17	17	109	585	65.3	5.27	1.188	1.303	1.201	1.283
P27482	Calmodulin-like protein 3	CALML3	34.90	5	5	8	149	16.9	4.42	1.265	1.306	1.278	1.286
O94985	Calsyntenin-1	CLSTN1	2.24	2	2	2	981	109.7	4.91	1.676	1.307	1.693	1.287
O75787	Renin receptor	ATP6AP2	20.86	5	5	13	350	39.0	6.10	0.634	1.307	0.641	1.288
P61204	ADP-ribosylation factor 3	ARF3	23.76	4	4	9	181	20.6	7.43	1.038	1.308	1.049	1.288
Q15121	Astrocytic phosphoprotein PEA-15	PEA15	16.92	2	2	4	130	15.0	5.02	0.830	1.308	0.839	1.289
O96009	Napsin-A	NAPSA	8.33	5	5	13	420	45.4	6.61	1.161	1.309	1.174	1.290
P42785	Lysosomal Pro-X carboxypeptidase	PRCP	28.23	10	10	22	496	55.8	7.21	1.046	1.310	1.057	1.290
P50395	Rab GDP dissociation inhibitor beta	GDI2	17.30	6	6	12	445	50.6	6.47	0.950	1.316	0.960	1.296
Q5KU26	Collectin-12	COLEC12	4.99	4	4	11	742	81.5	5.69	1.694	1.317	1.712	1.297
P98164	Low-density lipoprotein receptor-related protein 2	LRP2	21.55	80	80	258	4655	521.6	5.08	1.439	1.318	1.455	1.299
P09525	Annexin A4	ANXA4	26.65	8	8	14	319	35.9	6.13	1.070	1.319	1.081	1.299
P06744	Glucose-6-phosphate isomerase	GPI	7.17	3	3	4	558	63.1	8.32	0.964	1.319	0.974	1.299
Q5R3I4	Tetratricopeptide repeat protein 38	TTC38	6.18	2	2	2	469	52.8	5.99	0.710	1.320	0.718	1.300
P27105	Erythrocyte band 7 integral membrane protein	STOM	21.18	5	5	8	288	31.7	7.88	1.492	1.320	1.508	1.300
Q8WWA1	Transmembrane protein 40	TMEM40	13.73	2	2	4	233	25.5	5.59	1.277	1.327	1.290	1.307
Q96KN2	Beta-Ala-His dipeptidase	CNDP1	7.10	2	2	2	507	56.7	5.30	1.836	1.327	1.855	1.307
O95398	Rap guanine nucleotide exchange factor 3	RAPGEF3	4.23	2	2	2	923	103.7	7.56	1.708	1.327	1.726	1.307
P03951	Coagulation factor XI	F11	4.64	2	2	2	625	70.1	8.10	0.836	1.333	0.845	1.313
P23142	Fibulin-1	FBLN1	12.38	6	6	13	703	77.2	5.22	0.692	1.336	0.699	1.316
O60494	Cubilin	CUBN	21.72	59	59	179	3623	398.5	5.35	1.292	1.337	1.306	1.317
P51688	N-sulphoglucosamine sulphohydrolase	SGSH	7.37	3	3	10	502	56.7	6.95	1.328	1.338	1.342	1.317
Q9NPY3	Complement component C1q receptor	CD93	5.98	3	3	3	652	68.5	5.44	0.903	1.347	0.913	1.326
P36405	ADP-ribosylation factor-like protein 3	ARL3	19.78	3	3	5	182	20.4	7.24	1.614	1.347	1.631	1.327
P09466	Glycodelin	PAEP	23.89	3	3	8	180	20.6	5.57	0.150	1.349	0.152	1.329
P03973	Antileukoproteinase	SLPI	36.36	5	5	12	132	14.3	8.75	0.172	1.349	0.173	1.329
Q99497	Protein/nucleic acid deglycase DJ-1	PARK7	19.05	3	3	6	189	19.9	6.79	0.592	1.350	0.598	1.330
P60709	Actin, cytoplasmic 1	ACTB	51.73	14	14	89	375	41.7	5.48	1.560	1.352	1.577	1.331
Q9H6S3	Epidermal growth factor receptor kinase substrate 8-like protein 2	EPS8L2	10.21	5	5	9	715	80.6	6.84	1.275	1.354	1.289	1.334
O00584	Ribonuclease T2	RNASET2	31.25	8	8	29	256	29.5	7.08	1.448	1.355	1.464	1.335
P15313	V-type proton ATPase subunit B, kidney isoform	ATP6V1B1	6.24	2	2	2	513	56.8	5.66	0.938	1.355	0.948	1.335
P21266	Glutathione S-transferase Mu 3	GSTM3	47.56	12	12	22	225	26.5	5.54	0.509	1.358	0.515	1.338
Q14210	Lymphocyte antigen 6D	LY6D	23.44	3	3	5	128	13.3	8.21	1.937	1.365	1.957	1.344
Q02388	Collagen alpha-1(VII) chain	COL7A1	1.43	3	3	3	2944	295.0	6.27	1.122	1.366	1.134	1.345
P40926	Malate dehydrogenase, mitochondrial	MDH2	8.58	2	2	4	338	35.5	8.68	1.046	1.367	1.057	1.346
Q9UL46	Proteasome activator complex subunit 2	PSME2	10.46	2	2	2	239	27.4	5.73	0.816	1.368	0.825	1.347
P40121	Macrophage-capping protein	CAPG	22.99	7	7	19	348	38.5	6.19	0.790	1.368	0.799	1.347
Q9ULZ3	Apoptosis-associated speck-like protein containing a CARD	PYCARD	8.72	2	2	2	195	21.6	6.34	2.668	1.373	2.697	1.352
P36955	Pigment epithelium-derived factor	SERPINF1	21.77	9	9	15	418	46.3	6.38	1.438	1.378	1.453	1.357
P07476	Involucrin	IVL	37.26	16	16	36	585	68.4	4.61	1.651	1.381	1.669	1.360
O95980	Reversion-inducing cysteine-rich protein with Kazal motifs	RECK	6.90	5	5	8	971	106.4	6.74	0.990	1.383	1.000	1.362
P08670	Vimentin	VIM	15.02	6	6	10	466	53.6	5.12	3.678	1.384	3.717	1.363
P04083	Annexin A1	ANXA1	46.24	13	13	40	346	38.7	7.02	1.727	1.387	1.745	1.366
P61160	Actin-related protein 2	ACTR2	7.11	2	2	3	394	44.7	6.74	1.477	1.389	1.493	1.368
Q9UNN8	Endothelial protein C receptor	PROCR	25.63	5	5	81	238	26.7	7.18	0.820	1.391	0.829	1.370
P01782	Immunoglobulin heavy variable 3-9	IGHV3-9	25.42	2	2	3	118	12.9	7.08	1.984	1.394	2.006	1.373
P61026	Ras-related protein Rab-10	RAB10	15.00	3	3	9	200	22.5	8.38	0.787	1.397	0.796	1.375
P16066	Atrial natriuretic peptide receptor 1	NPR1	3.11	2	2	2	1061	118.8	6.64	1.125	1.397	1.137	1.376
P28799	Progranulin	GRN	19.22	8	8	36	593	63.5	6.83	0.968	1.398	0.978	1.377
Q9NQ38	Serine protease inhibitor Kazal-type 5	SPINK5	11.56	9	9	20	1064	120.6	8.06	1.974	1.401	1.995	1.380
Q02413	Desmoglein-1	DSG1	4.10	3	3	5	1049	113.7	5.03	1.107	1.401	1.119	1.380
Q9UKS6	Protein kinase C and casein kinase substrate in neurons protein 3	PACSIN3	6.37	2	2	2	424	48.5	6.18	1.029	1.402	1.040	1.380
Q9H4M9	EH domain-containing protein 1	EHD1	15.73	7	7	9	534	60.6	6.83	1.317	1.402	1.331	1.381
P01019	Angiotensinogen	AGT	33.20	10	10	52	485	53.1	6.32	1.964	1.403	1.985	1.382
Q09666	Neuroblast differentiation-associated protein AHNAK	AHNAK	11.70	17	17	27	5890	628.7	6.15	1.234	1.405	1.247	1.384
Q12794	Hyaluronidase-1	HYAL1	26.21	8	8	17	435	48.3	6.77	1.345	1.406	1.359	1.385
P04080	Cystatin-B	CSTB	58.16	3	3	6	98	11.1	7.56	1.926	1.409	1.946	1.387
Q96RF0	Sorting nexin-18	SNX18	7.17	2	2	3	628	68.9	5.68	1.031	1.409	1.042	1.388
P50897	Palmitoyl-protein thioesterase 1	PPT1	20.26	5	5	11	306	34.2	6.52	1.531	1.411	1.547	1.390
O14786	Neuropilin-1	NRP1	3.25	2	2	2	923	103.1	5.88	0.926	1.414	0.936	1.392
P09543	2',3'-cyclic-nucleotide 3'-phosphodiesterase	CNP	8.55	2	2	4	421	47.5	9.07	0.771	1.422	0.779	1.400
P67936	Tropomyosin alpha-4 chain	TPM4	15.73	5	5	9	248	28.5	4.69	0.976	1.426	0.986	1.404
P04217	Alpha-1B-glycoprotein	A1BG	27.07	10	10	81	495	54.2	5.86	2.279	1.426	2.303	1.405
Q06323	Proteasome activator complex subunit 1	PSME1	17.67	4	4	6	249	28.7	6.02	1.381	1.428	1.396	1.406
P07339	Cathepsin D	CTSD	33.25	11	11	65	412	44.5	6.54	1.574	1.428	1.591	1.407
P09960	Leukotriene A-4 hydrolase	LTA4H	5.40	3	3	5	611	69.2	6.18	2.524	1.428	2.551	1.407
O75326	Semaphorin-7A	SEMA7A	3.15	2	2	2	666	74.8	7.64	1.063	1.432	1.074	1.411
P12814	Alpha-actinin-1	ACTN1	13.34	4	10	20	892	103.0	5.41	0.791	1.432	0.799	1.411
O75891	Cytosolic 10-formyltetrahydrofolate dehydrogenase	ALDH1L1	2.55	2	2	2	902	98.8	5.94	1.397	1.433	1.412	1.411
Q8NHP8	Putative phospholipase B-like 2	PLBD2	15.28	7	7	19	589	65.4	6.80	1.471	1.433	1.486	1.411

Q96PD5	N-acetylmuramoyl-L-alanine amidase	PGLYRP2	27.26	8	8	27	576	62.2	7.55	1.708	1.439	1.727	1.417
P0DMV9	Heat shock 70 kDa protein 1B	HSPA1B	22.93	10	13	26	641	70.0	5.66	1.228	1.440	1.241	1.418
Q9Y490	Talin-1	TLN1	1.65	2	2	2	2541	269.6	6.07	0.832	1.442	0.841	1.420
A0A075B6	Immunoglobulin lambda variable 7-46	IGLV7-46	15.38	2	2	4	117	12.5	7.20	2.603	1.442	2.631	1.420
P04278	Sex hormone-binding globulin	SHBG	13.68	3	3	4	402	43.8	6.71	3.008	1.445	3.040	1.423
P62805	Histone H4	H4C1	31.07	3	3	11	103	11.4	11.36	1.955	1.446	1.976	1.424
P46940	Ras GTPase-activating-like protein IQGAP1	IQGAP1	2.90	3	3	4	1657	189.1	6.48	1.791	1.446	1.811	1.424
P18669	Phosphoglycerate mutase 1	PGAM1	31.10	6	6	11	254	28.8	7.18	0.876	1.447	0.885	1.426
O15197	Ephrin type-B receptor 6	EPHB6	8.81	6	6	11	1021	110.6	6.65	1.037	1.449	1.048	1.427
P06312	Immunoglobulin kappa variable 4-1	IGKV4-1	17.36	2	2	14	121	13.4	5.25	1.823	1.455	1.843	1.433
O00468	Agrin	AGRN	7.35	11	11	19	2068	217.2	6.39	1.322	1.458	1.336	1.436
B9A064	Immunoglobulin lambda-like polypeptide 5	IGLL5	40.19	2	8	337	214	23.0	8.84	1.999	1.459	2.020	1.437
Q6XQN6	Nicotinate phosphoribosyltransferase	NAPRT	11.71	4	4	4	538	57.5	5.68	0.856	1.460	0.865	1.438
Q8NBS9	Thioredoxin domain-containing protein 5	TXNDC5	8.56	3	3	3	432	47.6	5.97	0.701	1.473	0.708	1.451
P10599	Thioredoxin	TXN	40.95	4	4	31	105	11.7	4.92	1.304	1.480	1.318	1.457
Q9Y6X5	Bis(5'-adenosyl)-triphosphatase ENPP4	ENPP4	7.06	3	3	4	453	51.6	6.15	2.702	1.480	2.731	1.458
Q10588	ADP-ribosyl cyclase/cyclic ADP-ribose hydrolase 2	BST1	26.10	6	6	9	318	35.7	7.80	1.011	1.488	1.022	1.466
P03950	Angiogenin	ANG	15.65	2	2	2	147	16.5	9.64	0.860	1.490	0.869	1.468
Q86UN3	Reticulon-4 receptor-like 2	RTN4RL2	11.67	4	4	6	420	46.1	7.62	1.147	1.491	1.159	1.468
P62873	Guanine nucleotide-binding protein G(I)/G(S)/G(T) subunit beta-1	GNB1	14.41	2	5	6	340	37.4	6.00	1.550	1.492	1.567	1.470
Q6PCB0	von Willebrand factor A domain-containing protein 1	VWA1	8.31	2	2	3	445	46.8	7.68	0.995	1.494	1.006	1.471
Q92956	Tumor necrosis factor receptor superfamily member 14	TNFRSF14	9.54	2	2	6	283	30.4	7.15	0.767	1.498	0.775	1.476
P19957	Elafin	PI3	28.21	3	3	6	117	12.3	8.82	1.365	1.502	1.379	1.479
P02545	Prelamin-A/C	LMNA	3.31	2	2	2	664	74.1	7.02	1.394	1.509	1.409	1.486
P08294	Extracellular superoxide dismutase [Cu-Zn]	SOD3	30.42	6	6	27	240	25.8	6.61	1.545	1.511	1.562	1.488
P01619	Immunoglobulin kappa variable 3-20	IGKV3-20	46.55	3	4	48	116	12.5	4.96	2.612	1.522	2.640	1.499
P54108	Cysteine-rich secretory protein 3	CRISP3	9.80	3	3	5	245	27.6	7.80	0.467	1.523	0.472	1.500
P31949	Protein S100-A11	S100A11	42.86	4	4	13	105	11.7	7.12	1.416	1.527	1.431	1.504
Q9NUM4	Transmembrane protein 106B	TMEM106B	6.57	2	2	2	274	31.1	6.99	2.075	1.528	2.097	1.505
Q6W4X9	Mucin-6	MUC6	3.53	7	7	11	2439	256.9	7.39	0.143	1.530	0.145	1.507
O60814	Histone H2B type 1-K	H2BC12	33.33	4	4	12	126	13.9	10.32	1.670	1.533	1.687	1.510
P04040	Catalase	CAT	15.18	8	8	10	527	59.7	7.39	3.008	1.533	3.040	1.510
P13688	Carcinoembryonic antigen-related cell adhesion molecule 1	CEACAM1	10.46	3	3	3	526	57.5	5.97	1.483	1.534	1.499	1.511
P07355	Annexin A2	ANXA2	62.83	20	20	61	339	38.6	7.75	1.266	1.535	1.279	1.512
P08238	Heat shock protein HSP 90-beta	HSP90AB1	10.22	2	7	12	724	83.2	5.03	0.545	1.535	0.551	1.512
P25325	3-mercaptopyruvate sulfurtransferase	MPST	41.08	8	8	12	297	33.2	6.60	1.124	1.536	1.136	1.513
P17516	Aldo-keto reductase family 1 member C4	AKR1C4	6.50	2	2	2	323	37.0	6.93	1.418	1.536	1.433	1.513
Q03405	Urokinase plasminogen activator surface receptor	PLAUR	15.52	3	3	7	335	37.0	6.65	1.306	1.537	1.320	1.514
P01011	Alpha-1-antichymotrypsin	SERPINA3	45.15	18	18	119	423	47.6	5.52	1.588	1.538	1.605	1.515
P01780	Immunoglobulin heavy variable 3-7	IGHV3-7	32.48	3	4	13	117	12.9	6.57	2.105	1.542	2.127	1.519
P01834	Immunoglobulin kappa constant	IGKC	85.98	8	8	631	107	11.8	6.52	1.927	1.553	1.948	1.530
P01594	Immunoglobulin kappa variable 1-33	IGKV1-33	34.19	2	2	19	117	12.8	4.78	1.665	1.556	1.682	1.533
Q9H756	Leucine-rich repeat-containing protein 19	LRRC19	12.43	3	3	8	370	42.3	5.12	0.864	1.557	0.873	1.534
P0C0L5	Complement C4-B	C4B	39.97	3	49	156	1744	192.6	7.27	2.004	1.562	2.025	1.538
P20061	Transcobalamin-1	TCN1	13.39	4	4	6	433	48.2	5.03	1.176	1.563	1.188	1.540
P63104	14-3-3 protein zeta/delta	YWHAZ	37.55	6	9	22	245	27.7	4.79	0.895	1.563	0.905	1.540
P36952	Serpin B5	SERPINB5	9.33	3	3	3	375	42.1	6.05	1.343	1.565	1.357	1.542
P52566	Rho GDP-dissociation inhibitor 2	ARHGDIB	14.93	2	2	5	201	23.0	5.21	0.742	1.568	0.750	1.545
P51159	Ras-related protein Rab-27A	RAB27A	16.29	3	3	3	221	24.9	5.22	0.568	1.568	0.574	1.545
O00299	Chloride intracellular channel protein 1	CLIC1	15.77	3	3	4	241	26.9	5.17	0.701	1.569	0.708	1.545
P18206	Vinculin	VCL	5.73	6	6	10	1134	123.7	5.66	1.511	1.570	1.527	1.547
P60660	Myosin light polypeptide 6	MYL6	18.54	3	3	3	151	16.9	4.65	0.934	1.587	0.944	1.564
Q9UBX7	Kallikrein-11	KLK11	20.57	5	5	7	282	31.0	8.94	0.447	1.588	0.452	1.564
P99999	Cytochrome c	CYCS	37.14	4	4	9	105	11.7	9.57	1.069	1.591	1.080	1.567
Q9H0B8	Cysteine-rich secretory protein LCCL domain-containing 2	CRISPLD2	5.23	2	2	4	497	55.9	8.02	0.594	1.592	0.601	1.568
P02458	Collagen alpha-1(II) chain	COL2A1	2.42	2	3	5	1487	141.7	6.92	1.275	1.605	1.288	1.581
P20142	Gastricsin	PGC	5.15	2	2	7	388	42.4	4.46	0.096	1.608	0.097	1.583
P62328	Thymosin beta-4	TMSB4X	40.91	2	2	5	44	5.0	5.06	4.848	1.612	4.899	1.587
P59665	Neutrophil defensin 1	DEFA1	20.21	3	3	9	94	10.2	6.99	12.482	1.613	12.615	1.589
P20810	Calpastatin	CAST	8.90	5	5	6	708	76.5	5.07	1.365	1.617	1.379	1.593
P04155	Trefoil factor 1	TFF1	29.76	2	2	10	84	9.1	4.35	2.073	1.624	2.095	1.599
P07237	Protein disulfide-isomerase	P4HB	20.47	11	11	23	508	57.1	4.87	0.948	1.628	0.958	1.604
P01009	Alpha-1-antitrypsin	SERPINA1	66.75	29	29	738	418	46.7	5.59	2.249	1.629	2.274	1.605
P02671	Fibrinogen alpha chain	FGA	26.33	18	18	111	866	94.9	6.01	1.361	1.630	1.375	1.606
P35579	Myosin-9	MYH9	8.06	12	12	21	1960	226.4	5.60	1.055	1.632	1.066	1.607
P17405	Sphingomyelin phosphodiesterase	SMPD1	3.65	2	2	2	631	69.9	7.28	1.131	1.635	1.143	1.610
P11684	Uteroglobin	SCGB1A1	19.78	3	3	56	91	10.0	5.06	0.785	1.639	0.794	1.615
Q9UKR3	Kallikrein-13	KLK13	21.30	5	5	9	277	30.6	8.46	2.296	1.641	2.320	1.616
P29401	Transketolase	TKT	5.94	4	4	4	623	67.8	7.66	1.848	1.642	1.868	1.617
Q13822	Ectonucleotide pyrophosphatase/phosphodiesterase family member 2	ENPP2	4.98	3	3	4	863	98.9	7.37	1.097	1.645	1.109	1.620
P14618	Pyruvate kinase PKM	PKM	31.26	13	13	23	531	57.9	7.84	1.250	1.646	1.263	1.621
Q92876	Kallikrein-6	KLK6	26.64	5	5	10	244	26.8	7.44	2.246	1.647	2.270	1.622
Q9UBC9	Small proline-rich protein 3	SPRR3	76.33	10	10	135	169	18.1	8.57	1.823	1.655	1.842	1.630
Q9H223	EH domain-containing protein 4	EHD4	9.61	4	4	7	541	61.1	6.76	1.233	1.656	1.246	1.631
P19652	Alpha-1-acid glycoprotein 2	ORM2	43.78	6	9	147	201	23.6	5.11	1.970	1.657	1.991	1.632
O00187	Mannan-binding lectin serine protease 2	MASP2	11.08	8	8	59	686	75.7	5.63	1.928	1.664	1.949	1.639
P07911	Uromodulin	UMOD	44.38	26	26	1057	640	69.7	5.24	0.851	1.665	0.860	1.640
P06703	Protein S100-A6	S100A6	40.00	4	4	14	90	10.2	5.48	0.639	1.665	0.645	1.640
Q13938	Calcyphosin	CAPS	14.91	4	4	6	275	30.2	6.04	0.693	1.667	0.700	1.641
P13797	Plastin-3	PLS3	5.56	2	3	3	630	70.8	5.60	1.334	1.667	1.348	1.642
P02766	Transthyretin	TTR	64.63	7	7	84	147	15.9	5.76	1.574	1.669	1.591	1.644
P01876	Immunoglobulin heavy constant alpha 1	IGHA1	56.37	10	16	244	353	37.6	6.51	1.814	1.683	1.833	1.658
P12821	Angiotensin-converting enzyme	ACE	9.95	8	8	10	1306	149.6	6.39	0.695	1.689	0.703	1.663
P15121	Aldo-keto reductase family 1 member B1	AKR1B1	11.71	4	4	6	316	35.8	6.98	1.010	1.690	1.021	1.665
P04075	Fructose-bisphosphate aldolase A	ALDOA	32.97	10	11	20	364	39.4	8.09	1.462	1.707	1.478	1.682
Q9Y6R7	IgGFc-binding protein	FCGBP	4.83	12	12	19	5405	571.6	5.34	0.674	1.721	0.681	1.695
O60437	Periplakin	PPL	3.30	6	6	9	1756	204.6	5.60	1.812	1.723	1.831	1.697
P00450	Ceruloplasmin	CP	30.42	25	25	112	1065	122.1	5.72	2.628	1.726	2.656	1.700
O14745	Na(+)/H(+) exchange regulatory cofactor NHE-RF1	SLC9A3R1	20.39	5	5	7	358	38.8	5.77	0.867	1.732	0.876	1.706

P31944	Caspase-14	CASP14	35.95	8	8	14	242	27.7	5.58	2.081	1.740	2.103	1.714
A0A0B4J1U	Immunoglobulin heavy variable 6-1	IGHV6-1	21.49	3	3	6	121	13.5	9.20	1.594	1.742	1.611	1.716
P50502	Hsc70-interacting protein	ST13	7.05	2	2	2	369	41.3	5.27	0.938	1.743	0.948	1.717
P13164	Interferon-induced transmembrane protein 1	IFITM1	13.60	2	2	2	125	14.0	7.93	1.461	1.743	1.476	1.717
P30047	GTP cyclohydrolase 1 feedback regulatory protein	GCHFR	70.24	3	3	12	84	9.7	6.54	1.608	1.744	1.625	1.717
P05156	Complement factor I	CFI	25.56	11	11	28	583	65.7	7.50	1.728	1.746	1.747	1.720
P49908	Selenoprotein P	SELENOP	4.20	2	2	2	381	43.2	7.87	1.116	1.765	1.128	1.738
Q9UBG3	Cornulin	CRNN	37.37	11	11	27	495	53.5	6.10	1.886	1.768	1.907	1.742
P09871	Complement C1s subcomponent	C1S	9.59	5	5	6	688	76.6	4.96	1.362	1.781	1.376	1.754
Q6UX06	Olfactomedin-4	OLFM4	24.51	11	11	26	510	57.2	5.69	2.081	1.785	2.103	1.758
O95490	Adhesion G protein-coupled receptor L2	ADGRL2	1.78	2	2	2	1459	163.2	6.43	0.594	1.789	0.601	1.762
Q92692	Nectin-2	NECTIN2	8.92	4	4	15	538	57.7	4.82	1.219	1.802	1.232	1.775
P37837	Transaldolase	TALDO1	15.73	5	5	9	337	37.5	6.81	3.075	1.809	3.108	1.782
Q9NP55	BPI fold-containing family A member 1	BPIFA1	11.33	2	2	4	256	26.7	5.76	2.602	1.832	2.630	1.804
P00915	Carbonic anhydrase 1	CA1	14.18	3	3	7	261	28.9	7.12	4.314	1.848	4.360	1.820
P52209	6-phosphogluconate dehydrogenase, decarboxylating	PGD	21.12	8	8	15	483	53.1	7.23	1.628	1.854	1.646	1.827
P36980	Complement factor H-related protein 2	CFHR2	20.00	2	5	9	270	30.6	6.38	4.712	1.858	4.762	1.830
P04632	Calpain small subunit 1	CAPNS1	7.46	2	2	2	268	28.3	5.20	1.152	1.872	1.165	1.844
P02753	Retinol-binding protein 4	RBP4	58.21	9	9	43	201	23.0	6.07	1.960	1.872	1.981	1.844
P22392	Nucleoside diphosphate kinase B	NME2	35.53	4	4	5	152	17.3	8.41	1.406	1.882	1.421	1.854
Q9UHI8	A disintegrin and metalloproteinase with thrombospondin motifs 1	ADAMTS1	4.45	3	3	6	967	105.3	6.83	0.415	1.894	0.419	1.865
P29508	Serpin B3	SERPINB3	51.28	14	23	63	390	44.5	6.81	1.851	1.900	1.871	1.872
P32926	Desmoglein-3	DSG3	7.11	4	4	6	999	107.5	5.00	2.629	1.906	2.657	1.877
P00747	Plasminogen	PLG	39.14	26	26	74	810	90.5	7.24	2.173	1.923	2.197	1.894
P02790	Hemopexin	HPX	42.21	16	16	117	462	51.6	7.02	2.158	1.952	2.181	1.923
P29034	Protein S100-A2	S100A2	8.16	2	2	2	98	11.1	4.78	1.645	1.964	1.663	1.934
Q16610	Extracellular matrix protein 1	ECM1	12.22	6	6	12	540	60.6	6.71	0.248	1.973	0.250	1.943
P01768	Immunoglobulin heavy variable 3-30	IGHV3-30	24.79	2	3	7	117	12.9	8.92	2.132	2.004	2.155	1.974
O95445	Apolipoprotein M	APOM	27.66	4	4	9	188	21.2	6.01	2.505	2.008	2.532	1.978
P61626	Lysozyme C	LYZ	18.24	3	3	3	148	16.5	9.16	4.462	2.012	4.510	1.982
A0A0C4DH	Immunoglobulin kappa variable 1-8	IGKV1-8	26.96	2	2	2	115	12.5	9.01	1.396	2.017	1.411	1.986
P01033	Metalloproteinase inhibitor 1	TIMP1	23.19	4	4	8	207	23.2	8.10	0.345	2.045	0.349	2.014
Q9UIV8	Serpin B13	SERPINB13	18.16	6	6	7	391	44.2	5.71	2.072	2.049	2.094	2.018
P35321	Cornifin-A	SPRR1A	77.53	4	5	10	89	9.9	8.48	1.790	2.067	1.810	2.036
Q15848	Adiponectin	ADIPOQ	12.30	2	2	6	244	26.4	5.74	2.246	2.073	2.270	2.042
P05451	Lithostathine-1-alpha	REG1A	51.81	8	8	71	166	18.7	5.94	3.359	2.080	3.394	2.048
O43707	Alpha-actinin-4	ACTN4	15.70	6	12	18	911	104.8	5.44	1.309	2.093	1.323	2.061
P37802	Transgelin-2	TAGLN2	34.67	5	5	7	199	22.4	8.25	1.374	2.099	1.389	2.067
P06702	Protein S100-A9	S100A9	81.58	8	8	234	114	13.2	6.13	2.863	2.102	2.894	2.070
P05783	Keratin, type I cytoskeletal 18	KRT18	4.42	2	2	2	430	48.0	5.45	1.345	2.103	1.359	2.071
P01861	Immunoglobulin heavy constant gamma 4	IGHG4	43.73	4	10	199	327	35.9	7.36	1.970	2.104	1.991	2.072
Q32MZ4	Leucine-rich repeat flightless-interacting protein 1	LRRFIP1	4.70	3	3	3	808	89.2	4.65	1.480	2.112	1.495	2.080
P30740	Leukocyte elastase inhibitor	SERPINB1	25.33	9	9	16	379	42.7	6.28	2.485	2.156	2.512	2.124
P01614	Immunoglobulin kappa variable 2D-40	IGKV2D-40	28.10	3	3	62	121	13.3	4.61	2.358	2.160	2.383	2.128
A0A075B6	Immunoglobulin lambda variable 3-9	IGLV3-9	23.48	2	2	7	115	12.3	7.39	2.980	2.174	3.011	2.141
P49913	Cathelicidin antimicrobial peptide	CAMP	36.47	8	8	14	170	19.3	9.41	1.693	2.180	1.711	2.148
P05109	Protein S100-A8	S100A8	55.91	7	7	98	93	10.8	7.03	3.522	2.186	3.560	2.153
P02774	Vitamin D-binding protein	GC	58.02	28	28	115	474	52.9	5.45	3.289	2.188	3.324	2.155
P29373	Cellular retinoic acid-binding protein 2	CRABP2	28.99	4	4	4	138	15.7	5.40	1.579	2.214	1.596	2.180
P12429	Annexin A3	ANXA3	15.48	5	5	7	323	36.4	5.92	2.168	2.217	2.191	2.184
P01877	Immunoglobulin heavy constant alpha 2	IGHA2	43.82	5	11	102	340	36.6	6.27	1.100	2.217	1.111	2.184
P43652	Afamin	AFM	32.89	19	19	47	599	69.0	5.90	2.504	2.235	2.531	2.201
O75636	Ficolin-3	FCN3	6.69	2	2	2	299	32.9	6.67	3.327	2.239	3.362	2.205
A8K2U0	Alpha-2-macroglobulin-like protein 1	A2ML1	14.58	18	18	31	1454	161.0	5.73	2.275	2.244	2.299	2.211
P02768	Serum albumin	ALB	91.63	79	79	7760	609	69.3	6.28	2.741	2.246	2.770	2.212
P01008	Antithrombin-III	SERPINC1	44.18	18	18	61	464	52.6	6.71	2.882	2.274	2.913	2.240
P15169	Carboxypeptidase N catalytic chain	CPN1	5.46	2	2	2	458	52.3	7.34	4.526	2.284	4.575	2.250
P02763	Alpha-1-acid glycoprotein 1	ORM1	46.77	8	11	357	201	23.5	5.02	2.461	2.322	2.488	2.287
P08727	Keratin, type I cytoskeletal 19	KRT19	20.00	4	6	8	400	44.1	5.14	1.130	2.328	1.142	2.293
A0A0C4DH	Immunoglobulin heavy variable 1-3	IGHV1-3	28.21	3	3	7	117	13.0	9.55	1.904	2.342	1.924	2.307
A0A0B4J1	Immunoglobulin heavy variable 3-72	IGHV3-72	26.05	3	3	5	119	13.2	7.85	2.189	2.347	2.212	2.311
P33241	Lymphocyte-specific protein 1	LSP1	16.81	3	3	4	339	37.2	4.74	5.465	2.353	5.524	2.318
P01591	Immunoglobulin J chain	JCHAIN	36.48	5	5	23	159	18.1	5.24	1.154	2.369	1.167	2.333
P68871	Hemoglobin subunit beta	HBB	82.99	5	11	84	147	16.0	7.28	3.524	2.376	3.561	2.340
P06681	Complement C2	C2	5.72	4	4	8	752	83.2	7.42	3.588	2.417	3.626	2.380
P69905	Hemoglobin subunit alpha	HBA1	59.15	7	7	37	142	15.2	8.68	3.940	2.418	3.982	2.382
P00748	Coagulation factor XII	F12	4.39	2	2	2	615	67.7	7.74	2.164	2.440	2.187	2.403
Q9UHG3	Prenylcysteine oxidase 1	PCYOX1	4.36	2	2	2	505	56.6	6.18	1.840	2.448	1.859	2.411
P02042	Hemoglobin subunit delta	HBD	61.90	3	9	39	147	16.0	8.05	2.207	2.495	2.231	2.458
P48637	Glutathione synthetase	GSS	5.27	2	2	2	474	52.4	5.92	3.334	2.496	3.369	2.458
P14780	Matrix metalloproteinase-9	MMP9	27.16	14	14	28	707	78.4	6.06	2.499	2.497	2.525	2.459
P05164	Myeloperoxidase	MPO	12.48	7	7	12	745	83.8	8.97	5.194	2.516	5.250	2.478
P24158	Myeloblastin	PRTN3	7.81	2	2	3	256	27.8	8.35	2.792	2.566	2.822	2.527
P02787	Serotransferrin	TF	66.62	48	48	1020	698	77.0	7.12	3.611	2.583	3.650	2.544
Q01469	Fatty acid-binding protein 5	FABP5	50.37	8	8	45	135	15.2	7.01	1.602	2.601	1.619	2.562
P00736	Complement C1r subcomponent	C1R	6.38	4	5	7	705	80.1	6.21	3.680	2.606	3.720	2.566
P14207	Folate receptor beta	FOLR2	12.94	2	2	2	255	29.3	7.53	1.579	2.614	1.596	2.575
P80188	Neutrophil gelatinase-associated lipocalin	LCN2	50.00	8	8	46	198	22.6	8.91	3.059	2.650	3.091	2.610
Q6JBY9	CapZ-interacting protein	RCSD1	6.01	2	2	2	416	44.5	5.40	3.001	2.655	3.033	2.615
P07357	Complement component C8 alpha chain	C8A	5.14	3	3	5	584	65.1	6.47	3.011	2.655	3.043	2.615
P06727	Apolipoprotein A-IV	APOA4	34.60	12	12	30	396	45.4	5.38	3.765	2.662	3.805	2.622
Q14520	Hyaluronan-binding protein 2	HABP2	5.54	3	3	3	560	62.6	6.54	3.470	2.723	3.507	2.682
P55000	Secreted Ly-6/uPAR-related protein 1	SLURP1	15.53	2	2	3	103	11.2	5.33	1.260	2.742	1.273	2.701
P31151	Protein S100-A7	S100A7	67.33	8	8	210	101	11.5	6.77	1.863	2.768	1.883	2.727
P01031	Complement C5	C5	10.92	14	14	23	1676	188.2	6.52	3.407	2.788	3.444	2.746
P01857	Immunoglobulin heavy constant gamma 1	IGHG1	63.03	6	15	619	330	36.1	8.19	2.992	2.835	3.024	2.792
P36222	Chitinase-3-like protein 1	CHI3L1	6.53	2	2	2	383	42.6	8.46	3.044	2.864	3.077	2.821
P13671	Complement component C6	C6	7.07	7	7	13	934	104.7	6.76	4.491	2.876	4.539	2.833
P31947	14-3-3 protein sigma	SFN	43.55	8	9	20	248	27.8	4.74	1.779	2.922	1.798	2.877

P13796	Plastin-2	LCP1	36.36	17	18	31	627	70.2	5.43	3.786	2.964	3.827	2.919
P02748	Complement component C9	C9	11.63	6	6	13	559	63.1	5.59	4.134	2.986	4.178	2.941
P00751	Complement factor B	CFB	21.99	18	18	50	764	85.5	7.06	3.900	3.033	3.942	2.988
P05546	Heparin cofactor 2	SERPIND1	13.03	6	6	12	499	57.0	6.90	4.709	3.033	4.760	2.988
P05160	Coagulation factor XIII B chain	F13B	2.42	2	2	2	661	75.5	6.39	4.467	3.076	4.515	3.029
P20160	Azurocidin	AZU1	13.55	3	3	8	251	26.9	9.50	4.721	3.094	4.771	3.048
Q96RM1	Small proline-rich protein 2F	SPRR2F	43.06	2	3	9	72	7.8	8.31	2.811	3.102	2.841	3.055
P06331	Immunoglobulin heavy variable 4-34	IGHV4-34	37.40	3	3	4	123	13.8	9.33	2.615	3.112	2.643	3.066
P02743	Serum amyloid P-component	APCS	19.73	4	4	8	223	25.4	6.54	2.267	3.114	2.291	3.067
P09237	Matrilysin	MMP7	25.84	6	7	12	267	29.7	7.91	2.568	3.148	2.596	3.101
P26447	Protein S100-A4	S100A4	19.80	2	2	3	101	11.7	6.11	2.729	3.170	2.758	3.123
A0A0C4DI	Immunoglobulin heavy variable 1-18	IGHV1-18	23.93	2	2	3	117	12.8	8.84	1.838	3.348	1.857	3.298
P22532	Small proline-rich protein 2D	SPRR2D	54.17	3	4	24	72	7.9	8.37	2.252	3.385	2.276	3.334
P02647	Apolipoprotein A-I	APOA1	65.92	20	20	183	267	30.8	5.76	6.163	3.410	6.229	3.359
P06731	Carcinoembryonic antigen-related cell adhesion molecule 5	CEACAM5	4.56	2	3	3	702	76.7	5.92	1.738	3.543	1.757	3.489
P49411	Elongation factor Tu, mitochondrial	TUFM	3.54	2	2	2	452	49.5	7.61	6.219	3.602	6.285	3.548
P04114	Apolipoprotein B-100	APOB	10.69	43	43	58	4563	515.3	7.05	5.678	3.609	5.739	3.555
P05787	Keratin, type II cytoskeletal 8	KRT8	7.66	2	3	3	483	53.7	5.59	1.430	3.639	1.446	3.584
P02652	Apolipoprotein A-II	APOA2	41.00	5	5	14	100	11.2	6.62	11.010	3.739	11.127	3.683
P04003	C4b-binding protein alpha chain	C4BPA	7.04	4	4	9	597	67.0	7.30	2.549	3.769	2.577	3.712
P02654	Apolipoprotein C-I	APOC1	30.12	3	3	4	83	9.3	8.47	9.914	3.821	10.020	3.763
P04196	Histidine-rich glycoprotein	HRG	15.81	8	8	19	525	59.5	7.50	4.393	3.993	4.440	3.933
P19823	Inter-alpha-trypsin inhibitor heavy chain H2	ITIH2	12.37	10	10	21	946	106.4	6.86	5.049	4.027	5.103	3.967
Q08188	Protein-glutamine gamma-glutamyltransferase E	TGM3	11.83	7	7	13	693	76.6	5.86	2.200	4.110	2.223	4.048
P01023	Alpha-2-macroglobulin	A2M	38.60	46	46	168	1474	163.2	6.46	5.353	4.145	5.410	4.083
A0A075B6	Immunoglobulin lambda variable 3-10	IGLV3-10	20.87	2	2	2	115	12.4	4.83	6.293	4.250	6.360	4.186
Q96IY4	Carboxypeptidase B2	CPB2	4.96	2	2	2	423	48.4	7.71	4.828	4.261	4.880	4.197
P01860	Immunoglobulin heavy constant gamma 3	IGHG3	53.32	6	16	349	377	41.3	7.90	2.696	4.739	2.725	4.668
P03952	Plasma kallikrein	KLKB1	8.31	5	5	7	638	71.3	8.22	4.782	5.003	4.833	4.927
P02788	Lactotransferrin	LTF	64.23	40	40	166	710	78.1	8.12	1.463	5.083	1.479	5.007
P27169	Serum paraoxonase/arylesterase 1	PON1	7.04	2	2	2	355	39.7	5.22	11.571	5.245	11.695	5.166
P08603	Complement factor H	CFH	17.95	16	19	41	1231	139.0	6.61	3.934	5.262	3.976	5.183
P00739	Haptoglobin-related protein	HPR	46.55	5	16	217	348	39.0	7.09	4.740	5.270	4.790	5.191
P01024	Complement C3	C3	51.11	69	69	184	1663	187.0	6.40	4.531	5.410	4.579	5.328
P01880	Immunoglobulin heavy constant delta	IGHD	16.41	5	5	9	384	42.3	8.12	4.641	5.616	4.691	5.532
P22894	Neutrophil collagenase	MMP8	11.99	5	5	7	467	53.4	6.87	6.071	5.690	6.136	5.604
P0DJI8	Serum amyloid A-1 protein	SAA1	23.77	2	2	3	122	13.5	6.79	5.405	6.304	5.462	6.209
P00738	Haptoglobin	HP	62.32	12	23	413	406	45.2	6.58	6.891	6.323	6.965	6.228
P35542	Serum amyloid A-4 protein	SAA4	15.38	2	2	8	130	14.7	9.07	12.380	6.952	12.512	6.847
P02675	Fibrinogen beta chain	FGB	42.36	15	15	39	491	55.9	8.27	4.192	7.659	4.236	7.544
P19827	Inter-alpha-trypsin inhibitor heavy chain H1	ITIH1	8.89	8	8	17	911	101.3	6.79	8.133	8.117	8.219	7.995
P02679	Fibrinogen gamma chain	FGG	39.74	15	15	48	453	51.5	5.62	5.197	9.941	5.252	9.791
Q9NZT1	Calmodulin-like protein 5	CALML5	26.03	2	2	2	146	15.9	4.44		0.944		0.930
O76070	Gamma-synuclein	SNCG	23.62	2	2	2	127	13.3	4.86		0.966		0.952
P22528	Cornifin-B	SPRR1B	51.69	2	3	6	89	9.9	8.48		1.752		1.726
P48594	Serpin B4	SERPINB4	34.10	3	12	15	390	44.8	6.21		2.173		2.140
O76076	WNT1-inducible-signaling pathway protein 2	WISP2	15.60	3	3	4	250	26.8	7.88	0.873		0.882	
Q1KMD3	Heterogeneous nuclear ribonucleoprotein U-like protein 2	HNRNPUL2	3.61	2	2	2	747	85.1	4.91	1.089		1.101	

Table S5. List of the upregulated proteins in urine samples from ketamine abusers

Male ketamine abusers				
No.	Accession no.	Gene name	Fold change	Log2 (Fold change)
1	P59665	DEFA1	12.615	3.657
2	P35542	SAA4	12.512	3.645
3	P27169	PON1	11.695	3.548
4	P02652	APOA2	11.127	3.476
5	P02654	APOC1	10.020	3.325
6	P19827	ITIH1	8.219	3.039
7	P00738	HP	6.965	2.800
8	A0A075B6K4	IGLV3-10	6.360	2.669
9	P49411	TUFM	6.285	2.652
10	P02647	APOA1	6.229	2.639
11	P22894	MMP8	6.136	2.617
12	P04114	APOB	5.739	2.521
13	P33241	LSP1	5.524	2.466
14	P0DJI8	SAA1	5.462	2.450
15	P01023	A2M	5.410	2.436
16	P02679	FGG	5.252	2.393
17	P05164	MPO	5.250	2.392
18	P19823	ITIH2	5.103	2.351
19	P62328	TMSB4X	4.899	2.293
20	Q96IY4	CPB2	4.880	2.287
21	P03952	KLKB1	4.833	2.273
22	P00739	HPR	4.790	2.260
23	P20160	AZU1	4.771	2.254
24	P36980	CFHR2	4.762	2.252
25	P05546	SERPIND1	4.760	2.251
26	P01880	IGHD	4.691	2.230
27	P01024	C3	4.579	2.195
28	P15169	CPN1	4.575	2.194
29	P13671	C6	4.539	2.182
30	P05160	F13B	4.515	2.175
31	P61626	LYZ	4.510	2.173
32	P04196	HRG	4.440	2.150
33	P00915	CA1	4.360	2.124
34	P02675	FGB	4.236	2.083
35	P02748	C9	4.178	2.063
36	P69905	HBA1	3.982	1.994
37	P08603	CFH	3.976	1.991
38	P00751	CFB	3.942	1.979
39	P13796	LCP1	3.827	1.936
40	P06727	APOA4	3.805	1.928
41	P00736	C1R	3.720	1.895
42	P08670	VIM	3.717	1.894
43	P02787	TF	3.650	1.868
44	P06681	C2	3.626	1.858
45	P68871	HBB	3.561	1.832
46	P05109	S100A8	3.560	1.832
47	Q16661	GUCA2B	3.552	1.829
48	Q14520	HABP2	3.507	1.810
49	P01031	C5	3.444	1.784
50	P05451	REG1A	3.394	1.763
51	P48637	GSS	3.369	1.752
52	O75636	FCN3	3.362	1.749
53	P02774	GC	3.324	1.733
54	P37837	TALDO1	3.108	1.636
55	P80188	LCN2	3.091	1.628
56	P36222	CHI3L1	3.077	1.621
57	P07357	C8A	3.043	1.606
58	P04040	CAT	3.040	1.604
59	P04278	SHBG	3.040	1.604
60	Q6JBY9	RCSD1	3.033	1.601
61	P01857	IGHG1	3.024	1.596
62	A0A075B6K5	IGLV3-9	3.011	1.590
63	Q9BRF8	CPPED1	2.919	1.545
64	P01008	SERPINC1	2.913	1.542
65	P06702	S100A9	2.894	1.533
66	P0C0L4	C4A	2.888	1.530
67	Q96RM1	SPRR2F	2.841	1.506
68	P24158	PRTN3	2.822	1.497
69	P02750	LRG1	2.820	1.496
70	P02768	ALB	2.770	1.470
71	P26447	S100A4	2.758	1.464

Female ketamine abusers				
No.	Accession no.	Gene name	Fold change	Log2 (Fold change)
1	P02679	FGG	9.791	3.292
2	P19827	ITIH1	7.995	2.999
3	P02675	FGB	7.544	2.915
4	P35542	SAA4	6.847	2.776
5	P00738	HP	6.228	2.639
6	P0DJI8	SAA1	6.209	2.634
7	P22894	MMP8	5.604	2.487
8	P01880	IGHD	5.532	2.468
9	P01024	C3	5.328	2.414
10	P00739	HPR	5.191	2.376
11	P08603	CFH	5.183	2.374
12	P27169	PON1	5.166	2.369
13	P02788	LTF	5.007	2.324
14	P03952	KLKB1	4.927	2.301
15	P01860	IGHG3	4.668	2.223
16	Q96IY4	CPB2	4.197	2.069
17	A0A075B6K4	IGLV3-10	4.186	2.066
18	P01023	A2M	4.083	2.030
19	Q08188	TGM3	4.048	2.017
20	P19823	ITIH2	3.967	1.988
21	P04196	HRG	3.933	1.975
22	P02654	APOC1	3.763	1.912
23	P04003	C4BPA	3.712	1.892
24	P02652	APOA2	3.683	1.881
25	P05787	KRT8	3.584	1.842
26	P04114	APOB	3.555	1.830
27	P49411	TUFM	3.548	1.827
28	P06731	CEACAM5	3.489	1.803
29	P02647	APOA1	3.359	1.748
30	P22532	SPRR2D	3.334	1.737
31	A0A0C4DH31	IGHV1-18	3.298	1.721
32	P26447	S100A4	3.123	1.643
33	P09237	MMP7	3.101	1.633
34	P02743	APCS	3.067	1.617
35	P06331	IGHV4-34	3.066	1.616
36	Q96RM1	SPRR2F	3.055	1.611
37	P20160	AZU1	3.048	1.608
38	P05160	F13B	3.029	1.599
39	P05546	SERPIND1	2.988	1.579
40	P00751	CFB	2.988	1.579
41	P02748	C9	2.941	1.556
42	P13796	LCP1	2.919	1.546
43	P31947	SFN	2.877	1.525
44	P13671	C6	2.833	1.502
45	P36222	CHI3L1	2.821	1.496
46	P01857	IGHG1	2.792	1.481
47	P01031	C5	2.746	1.457
48	P31151	S100A7	2.727	1.447
49	P55000	SLURP1	2.701	1.433
50	Q14520	HABP2	2.682	1.423
51	P06727	APOA4	2.622	1.391
52	P07357	C8A	2.615	1.387
53	Q6JBY9	RCSD1	2.615	1.387
54	P80188	LCN2	2.610	1.384
55	P14207	FOLR2	2.575	1.364
56	P00736	C1R	2.566	1.360
57	Q01469	FABP5	2.562	1.357
58	P02787	TF	2.544	1.347
59	P24158	PRTN3	2.527	1.337
60	P05164	MPO	2.478	1.309
61	P14780	MMP9	2.459	1.298
62	P48637	GSS	2.458	1.298
63	P02042	HBD	2.458	1.297
64	Q9UHG3	PCYOX1	2.411	1.270
65	P00748	F12	2.403	1.265
66	P69905	HBA1	2.382	1.252
67	P06681	C2	2.380	1.251
68	P68871	HBB	2.340	1.227
69	P01591	JCHAIN	2.333	1.222
70	P33241	LSP1	2.318	1.213
71	A0A0B4J1Y9	IGHV3-72	2.311	1.209

72	Q9Y6X5	ENPP4	2.731	1.450
73	P01860	IGHG3	2.725	1.446
74	Q9ULZ3	PYCARD	2.697	1.431
75	P32926	DSG3	2.657	1.410
76	P00450	CP	2.656	1.409
77	P01871	IGHM	2.655	1.409
78	P06331	IGHV4-34	2.643	1.402
79	P01619	IGKV3-20	2.640	1.401
80	A0A075B6I9	IGLV7-46	2.631	1.396
81	Q9NP55	BPIFA1	2.630	1.395
82	P09237	MMP7	2.596	1.376
83	P04003	C4BPA	2.577	1.365
84	P09960	LTA4H	2.551	1.351
85	O95445	APOM	2.532	1.340
86	P43652	AFM	2.531	1.340
87	P14780	MMP9	2.525	1.336
88	P30740	SERPINB1	2.512	1.329
89	P02763	ORM1	2.488	1.315
90	P19013	KRT4	2.445	1.290
91	P35908	KRT2	2.390	1.257
92	P01614	IGKV2D-40	2.383	1.253
93	Q9UKR3	KLK13	2.320	1.214
94	P04217	A1BG	2.303	1.204
95	A8K2U0	A2ML1	2.299	1.201
96	P02743	APCS	2.291	1.196
97	P02533	KRT14	2.288	1.194
98	A0M8Q6	IGLC7	2.285	1.192
99	A0A0B4J1X5	IGHV3-74	2.283	1.191
100	P22532	SPRR2D	2.276	1.186
101	P01009	SERPINA1	2.274	1.185
102	Q92876	KLK6	2.270	1.183
103	Q15848	ADIPOQ	2.270	1.183
104	A0A0C4DH38	IGHV5-51	2.268	1.182
105	P02042	HBD	2.231	1.158
106	Q08188	TGM3	2.223	1.153
107	P07737	PFN1	2.219	1.150
108	A0A0B4J1Y9	IGHV3-72	2.212	1.145
109	P00747	PLG	2.197	1.135
110	P19320	VCAM1	2.196	1.135
111	P12429	ANXA3	2.191	1.132
112	P00748	F12	2.187	1.129
113	P02790	HPX	2.181	1.125
114	P01768	IGHV3-30	2.155	1.108
115	P01780	IGHV3-7	2.127	1.089
116	P0DOY2	IGLC2	2.118	1.083
117	P31944	CASP14	2.103	1.072
118	Q6UX06	OLFM4	2.103	1.072
119	Q9NUM4	TMEM106B	2.097	1.068
120	P04155	TFF1	2.095	1.067
121	Q9UIV8	SERPINB13	2.094	1.066
122	P0C0L5	C4B	2.025	1.018
123	B9A064	IGLL5	2.020	1.014
124	P01782	IGHV3-9	2.006	1.004
125	P13645	KRT10	2.000	1.000
126	Q9NQ38	SPINK5	1.995	0.996
127	P01861	IGHG4	1.991	0.994
128	P19652	ORM2	1.991	0.993
129	P01019	AGT	1.985	0.989
130	Q15828	CST6	1.984	0.989
131	P02753	RBP4	1.981	0.986
132	P62805	H4C1	1.976	0.982
133	P47756	CAPZB	1.971	0.979
134	Q07654	TFF3	1.970	0.978
135	P38571	LIPA	1.969	0.977
136	Q14210	LY6D	1.957	0.969
137	O00187	MASP2	1.949	0.963
138	P01834	IGKC	1.948	0.962
139	P04080	CSTB	1.946	0.961
140	Q8NCC3	PLA2G15	1.939	0.955
141	P04264	KRT1	1.931	0.950
142	A0A0C4DH29	IGHV1-3	1.924	0.944
143	P02538	KRT6A	1.920	0.941

72	A0A0C4DH29	IGHV1-3	2.307	1.206
73	P08727	KRT19	2.293	1.197
74	P02763	ORM1	2.287	1.193
75	P15169	CPN1	2.250	1.170
76	P01008	SERPINC1	2.240	1.163
77	P02768	ALB	2.212	1.146
78	A8K2U0	A2ML1	2.211	1.144
79	O75636	FCN3	2.205	1.141
80	P43652	AFM	2.201	1.138
81	P01877	IGHA2	2.184	1.127
82	P12429	ANXA3	2.184	1.127
83	P29373	CRABP2	2.180	1.124
84	P02774	GC	2.155	1.108
85	P05109	S100A8	2.153	1.107
86	P49913	CAMP	2.148	1.103
87	A0A075B6K5	IGLV3-9	2.141	1.098
88	P48594	SERPINB4	2.140	1.098
89	P01614	IGKV2D-40	2.128	1.089
90	P30740	SERPINB1	2.124	1.086
91	Q32MZ4	LRRFIP1	2.080	1.057
92	P01861	IGHG4	2.072	1.051
93	P05783	KRT18	2.071	1.050
94	P06702	S100A9	2.070	1.050
95	P37802	TAGLN2	2.067	1.048
96	O43707	ACTN4	2.061	1.043
97	P05451	REG1A	2.048	1.034
98	Q15848	ADIPOQ	2.042	1.030
99	P35321	SPRR1A	2.036	1.026
100	Q9UIV8	SERPINB13	2.018	1.013
101	P01033	TIMP1	2.014	1.010
102	A0A0C4DH67	IGKV1-8	1.986	0.990
103	P61626	LYZ	1.982	0.987
104	O95445	APOM	1.978	0.984
105	P01768	IGHV3-30	1.974	0.981
106	Q16610	ECM1	1.943	0.958
107	P29034	S100A2	1.934	0.952
108	P02790	HPX	1.923	0.943
109	P00747	PLG	1.894	0.921
110	P32926	DSG3	1.877	0.909
111	P29508	SERPINB3	1.872	0.904
112	Q9UHI8	ADAMTS1	1.865	0.899
113	P22392	NME2	1.854	0.891
114	P02753	RBP4	1.844	0.883
115	P04632	CAPNS1	1.844	0.883
116	P36980	CFHR2	1.830	0.872
117	P52209	PGD	1.827	0.869
118	P00915	CA1	1.820	0.864
119	Q9NP55	BPIFA1	1.804	0.851
120	P37837	TALDO1	1.782	0.833
121	Q92692	NECTIN2	1.775	0.828
122	O95490	ADGRL2	1.762	0.817
123	Q6UX06	OLFM4	1.758	0.814
124	P09871	C1S	1.754	0.811
125	Q9UBG3	CRNN	1.742	0.800
126	P49908	SELENOP	1.738	0.797
127	P22528	SPRR1B	1.726	0.787
128	P05156	CFI	1.720	0.782
129	P30047	GCHFR	1.717	0.780
130	P13164	IFITM1	1.717	0.780
131	P50502	ST13	1.717	0.780
132	A0A0B4J1U7	IGHV6-1	1.716	0.779
133	P31944	CASP14	1.714	0.777
134	O14745	SLC9A3R1	1.706	0.771
135	P00450	CP	1.700	0.766
136	O60437	PPL	1.697	0.763
137	Q9Y6R7	FCGBP	1.695	0.762

Table S6. List of the downregulated proteins in urine samples from ketamine abusers

Male ketamine abusers				
No.	Accession no.	Gene name	Fold change	Log2 (Fold change)
1	P54107	CRISP1	0.085	-3.554
2	P80303	NUCB2	0.092	-3.439
3	P20142	PGC	0.097	-3.367
4	P04279	SEMG1	0.115	-3.126
5	Q8N4F0	BPIFB2	0.137	-2.867
6	P01036	CST4	0.140	-2.836
7	Q6W4X9	MUC6	0.145	-2.787
8	P12273	PIP	0.146	-2.777
9	P09466	PAEP	0.152	-2.722
10	Q02383	SEMG2	0.171	-2.552
11	P03973	SLPI	0.173	-2.527
12	Q9HC84	MUC5B	0.206	-2.280
13	A6NL88	SHISA7	0.216	-2.211
14	P49221	TGM4	0.237	-2.076
15	P02792	FTL	0.238	-2.070
16	Q16610	ECM1	0.250	-1.998
17	O95460	MATN4	0.269	-1.892
18	P07204	THBD	0.271	-1.881
19	Q8WWV6	FCAMR	0.279	-1.840
20	P15428	HPGD	0.285	-1.809
21	P56537	EIF6	0.312	-1.682
22	P29972	AQP1	0.329	-1.606
23	P01033	TIMP1	0.349	-1.520
24	Q8N307	MUC20	0.350	-1.515
25	Q96MG2	JSRP1	0.376	-1.411
26	Q8IUK5	PLXDC1	0.388	-1.365
27	P21802	FGFR2	0.392	-1.353
28	Q9Y653	ADGRG1	0.394	-1.345
29	Q13449	LSAMP	0.394	-1.342
30	P06858	LPL	0.396	-1.335
31	P15309	ACPP	0.400	-1.321
32	P55291	CDH15	0.413	-1.277
33	O75874	IDH1	0.419	-1.255
34	Q16363	LAMA4	0.419	-1.254
35	Q9UHI8	ADAMTS1	0.419	-1.254
36	Q8WWA0	ITLN1	0.420	-1.250
37	P54710	FXYD2	0.422	-1.246
38	P80723	BASP1	0.432	-1.210
39	P07288	KLK3	0.449	-1.156
40	Q9ULI3	HEG1	0.449	-1.154
41	Q9UBX7	KLK11	0.452	-1.146
42	Q92597	NDRG1	0.458	-1.127
43	P01210	PENK	0.460	-1.119
44	P25940	COL5A3	0.462	-1.114
45	Q86TY3	ARMH4	0.463	-1.111
46	P39059	COL15A1	0.470	-1.090
47	P54108	CRISP3	0.472	-1.084
48	Q96GW7	BCAN	0.477	-1.068
49	P09958	FURIN	0.477	-1.067
50	Q5TFQ8	SIRPB1	0.478	-1.064
51	Q14314	FGL2	0.481	-1.056
52	Q86VP6	CAND1	0.487	-1.039
53	P08519	LPA	0.494	-1.017
54	Q16849	PTPRN	0.496	-1.012
55	P54289	CACNA2D	0.497	-1.008
56	Q08174	PCDH1	0.499	-1.004
57	P15151	PVR	0.501	-0.997
58	P31431	SDC4	0.501	-0.997
59	Q8IYS5	OSCAR	0.501	-0.996
60	O75347	TBCA	0.508	-0.976
61	Q96FE7	PIK3IP1	0.510	-0.973
62	Q92859	NEO1	0.510	-0.972
63	P21266	GSTM3	0.515	-0.959
64	O43852	CALU	0.515	-0.959
65	P41271	NBL1	0.517	-0.951
66	Q96MU8	KREMEN1	0.518	-0.948
67	P23471	PTPRZ1	0.524	-0.932

Female ketamine abusers				
No.	Accession no.	Gene name	Fold change	Log2 (Fold change)
1	Q8WZ42	TTN	0.281	-1.832
2	Q71RC9	SMIM5	0.390	-1.358
3	Q16849	PTPRN	0.411	-1.281
4	P23471	PTPRZ1	0.422	-1.244
5	P31431	SDC4	0.426	-1.232
6	Q16363	LAMA4	0.428	-1.223
7	Q86Y38	XYLT1	0.443	-1.175
8	Q86X29	LSR	0.444	-1.171
9	P80370	DLK1	0.450	-1.152
10	Q16832	DDR2	0.479	-1.062
11	O95865	DDAH2	0.483	-1.050
12	Q96GW7	BCAN	0.490	-1.028
13	Q9BRT3	MIEN1	0.504	-0.989
14	P05060	CHGB	0.520	-0.942
15	P25940	COL5A3	0.530	-0.916
16	P22891	PROZ	0.533	-0.908
17	Q9NNX6	CD209	0.535	-0.903
18	P39059	COL15A1	0.535	-0.903
19	Q8TDY8	IGDCC4	0.541	-0.887
20	Q6FHJ7	SFRP4	0.544	-0.879
21	Q9Y279	VSIG4	0.550	-0.862
22	A6NL88	SHISA7	0.563	-0.830
23	P35613	BSG	0.566	-0.821
24	Q8WWV6	FCAMR	0.567	-0.819
25	O14798	TNFRSF10	0.568	-0.816
26	P51693	APLP1	0.573	-0.803
27	Q14982	OPCML	0.575	-0.799
28	P13727	PRG2	0.576	-0.796
29	Q96FE7	PIK3IP1	0.578	-0.791
30	Q86TY3	ARMH4	0.579	-0.788
31	Q96RW7	HMCN1	0.582	-0.781
32	Q9ULI3	HEG1	0.584	-0.775
33	O15240	VGF	0.589	-0.763
34	Q9P121	NTM	0.591	-0.760
35	Q8N3J6	CADM2	0.592	-0.757
36	P01210	PENK	0.592	-0.756
37	P05026	ATP1B1	0.595	-0.748
38	P15151	PVR	0.596	-0.746
39	P21695	GPD1	0.597	-0.743
40	P10451	SPP1	0.600	-0.738
41	P09210	GSTA2	0.600	-0.737
42	P21810	BGN	0.600	-0.737
43	Q93088	BHMT	0.603	-0.730
44	Q96DA0	ZG16B	0.603	-0.730
45	Q8WU39	MZB1	0.605	-0.724
46	P09564	CD7	0.607	-0.721
47	P55287	CDH11	0.607	-0.720
48	P41217	CD200	0.612	-0.709
49	Q9H159	CDH19	0.612	-0.707
50	P09619	PDGFRB	0.613	-0.707
51	P40197	GP5	0.613	-0.707
52	Q16769	QPCT	0.615	-0.700
53	Q13445	TMED1	0.616	-0.699
54	P31997	CEACAM8	0.618	-0.694
55	P05937	CALB1	0.619	-0.693
56	P16070	CD44	0.620	-0.689
57	O94856	NFASC	0.621	-0.688
58	Q6UXB8	PI16	0.624	-0.681
59	P36639	NUDT1	0.625	-0.677
60	Q6UXB4	CLEC4G	0.629	-0.669
61	Q9NU53	GINM1	0.631	-0.664
62	P21802	FGFR2	0.633	-0.660
63	P19013	KRT4	0.634	-0.657
64	Q08334	IL10RB	0.637	-0.651
65	Q96J84	KIRREL1	0.639	-0.646
66	P07148	FABP1	0.640	-0.645
67	A6NI73	LILRA5	0.640	-0.643

68	Q68D85	NCR3LG1	0.525	-0.929
69	Q8N6Q3	CD177	0.527	-0.923
70	Q8WVV5	BTN2A2	0.528	-0.921
71	O00499	BIN1	0.529	-0.920
72	P55259	GP2	0.529	-0.918
73	Q16832	DDR2	0.531	-0.913
74	P10451	SPP1	0.533	-0.908
75	Q13445	TMED1	0.534	-0.904
76	Q9NY25	CLEC5A	0.538	-0.893
77	Q96JQ0	DCHS1	0.539	-0.891
78	Q06828	FMOD	0.540	-0.890
79	O75144	ICOSLG	0.541	-0.887
80	P61916	NPC2	0.543	-0.880
81	Q6UXD5	SEZ6L2	0.544	-0.877
82	P22891	PROZ	0.547	-0.869
83	Q96DR8	MUCL1	0.550	-0.861
84	P08238	HSP90AB1	0.551	-0.860
85	P14625	HSP90B1	0.554	-0.852
86	Q5JXA9	SIRPB2	0.565	-0.824
87	Q8NBJ4	GOLM1	0.568	-0.817
88	P01034	CST3	0.570	-0.812
89	P51159	RAB27A	0.574	-0.801
90	P13611	VCAN	0.575	-0.799
91	P12277	CKB	0.576	-0.796
92	P22304	IDS	0.576	-0.795
93	O14798	TNFRSF10	0.578	-0.790
94	P36639	NUDT1	0.579	-0.787
95	P05060	CHGB	0.580	-0.785
96	Q9UN70	PCDHGC3	0.583	-0.778
97	P80370	DLK1	0.584	-0.775
98	P10586	PTPRF	0.585	-0.774
99	P05067	APP	0.585	-0.773
100	Q12841	FSTL1	0.586	-0.772
101	Q14118	DAG1	0.589	-0.763
102	P51148	RAB5C	0.594	-0.752
103	Q8TBP5	FAM174A	0.594	-0.752
104	Q8TDY8	IGDCC4	0.594	-0.752

68	Q6UXD5	SEZ6L2	0.644	-0.636
69	P11362	FGFR1	0.644	-0.635
70	P05062	ALDOB	0.646	-0.631
71	P56537	EIF6	0.646	-0.630
72	Q07954	LRP1	0.646	-0.629
73	O43493	TGOLN2	0.648	-0.625
74	Q96DR8	MUCL1	0.651	-0.620
75	P51884	LUM	0.651	-0.619
76	O00592	PODXL	0.652	-0.617
77	Q96JQ0	DCHS1	0.652	-0.617
78	Q496F6	CD300E	0.654	-0.612
79	P45877	PPIC	0.657	-0.605
80	P13611	VCAN	0.660	-0.599
81	P06734	FCER2	0.661	-0.597
82	Q15746	MYLK	0.663	-0.594
83	Q6GTX8	LAIR1	0.663	-0.593
84	P02462	COL4A1	0.665	-0.590
85	Q9UGM3	DMBT1	0.666	-0.587
86	Q8TAB3	PCDH19	0.666	-0.587
87	P01589	IL2RA	0.666	-0.586
88	P05556	ITGB1	0.667	-0.585
89	P22304	IDS	0.667	-0.585
90	Q12864	CDH17	0.670	-0.578
91	Q96S96	PEBP4	0.670	-0.577
92	P49747	COMP	0.670	-0.577
93	Q9BY67	CADM1	0.671	-0.575
94	P11279	LAMP1	0.673	-0.571
95	Q9BRK3	MXRA8	0.675	-0.567
96	P19256	CD58	0.676	-0.566
97	O95460	MATN4	0.676	-0.565
98	Q6UXE8	BTNL3	0.676	-0.564
99	P07195	LDHB	0.678	-0.561
100	P55017	SLC12A3	0.680	-0.556
101	Q04756	HGFAC	0.681	-0.553
102	Q9H6X2	ANTXR1	0.682	-0.552
103	Q14894	CRYM	0.683	-0.550
104	P01034	CST3	0.684	-0.549
105	P78380	OLR1	0.684	-0.548
106	P25189	MPZ	0.685	-0.546
107	P30086	PEBP1	0.687	-0.543
108	P08123	COL1A2	0.688	-0.540
109	P16112	ACAN	0.688	-0.539
110	Q14019	COTL1	0.689	-0.538
111	Q8WZ75	ROBO4	0.689	-0.537
112	Q8TDQ0	HAVCR2	0.690	-0.535
113	P09958	FURIN	0.691	-0.534
114	Q96C23	GALM	0.692	-0.531
115	P36957	DLST	0.693	-0.530
116	Q9NY15	STAB1	0.695	-0.526
117	P49189	ALDH9A1	0.696	-0.523
118	P02538	KRT6A	0.698	-0.520

Table S7 . GO process network of enriched urine proteins in ketamine abusers of both genders generated using DAVID Bioinformatics resources

#	GO Term	Count	%	P-Value	Genes	Benjamini	FDR
1	complement activation	15	11.90	9.80E-16	FCN3, C5, IGHG3, IGHG4, C8A, IGKV2D-40, IGHV4-34, CFH, C6, CFB, IGHV3-30, IGHG1, C3, C1R, C2	8.27E-13	1.54E-12
2	complement activation, classical pathway	15	11.90	6.62E-15	C5, IGHG3, IGHG4, C8A, C4BPA, IGKV2D-40, IGHV4-34, C9, C6, IGHV3-30, IGHG1, IGHD, C3, C1R, C2	2.76E-12	1.03E-11
3	regulation of complement	9	7.14	2.25E-11	C5, CFB, C8A, C4BPA, C9, CFH, C6, C3,	6.20E-09	3.46E-08
4	proteolysis	21	16.67	2.40E-10	FCN3, IGHG3, IGHG4, IGKV2D-40, IGHV4-34, PRTN3, AZU1, MMP9, CASP14, MMP7, CFB, IGHV3-30, HABP2, IGHG1, MMP8, KLKB1, C3, PLG,	4.98E-08	3.71E-07
5	negative regulation of endopeptidase activity	12	9.52	7.82E-10	HRG, C5, SERPINB1, SERPIND1, SERPINC1, ITIH1, A2ML1, C3, ITIH2, A2M, SERPINB13, FURIN	1.30E-07	1.21E-06
6	extracellular matrix disassembly	10	7.94	2.73E-09	BCAN, MMP8, MMP9, KLKB1, SPP1, PLG, LCP1, A2M, MMP7, FURIN	3.77E-07	4.21E-06
7	fibrinolysis	7	5.56	5.01E-09	HRG, FGG, FGB, KLKB1, F12, PLG, CPB2	5.93E-07	7.72E-06
8	complement activation, alternative pathway	6	4.76	1.85E-08	C5, CFB, C8A, C9, CFH, C3	1.92E-06	2.86E-05
9	receptor-mediated endocytosis	12	9.52	7.17E-08	APOB, HBA1, IGHV3-30, SAA1, IGKV2D-40, ALB, IGHV4-34, HPR, HP, HPX, HBB, APOA1	6.60E-06	1.11E-04
10	lipoprotein metabolic process	7	5.56	2.31E-07	APOB, ALB, APOA4, APOM, APOA1, APOA2, APOC1	1.92E-05	3.57E-04
11	innate immune response	16	12.70	3.04E-07	IGHG3, S100A9, IGHG4, LCN2, SAA1, C4BPA, FGB, S100A8, C6, APCS, IGHG1, F12, IGHD, BPIFA1, C1R, C2	2.29E-05	4.69E-04
12	platelet degranulation	9	7.14	5.98E-07	HRG, ORM1, FGG, TF, FGB, ALB, APOA1, PLG, A2M	4.13E-05	9.22E-04
13	blood coagulation	11	8.73	6.33E-07	FGG, FGB, SERPIND1, PROZ, SERPINC1, PRTN3, HBD, F13B, HBB, PLG, CPB2	4.03E-05	9.76E-04
14	cholesterol efflux	6	4.76	7.16E-07	APOB, APOA4, APOM, APOA1, APOA2, APOC1	4.24E-05	0.0011
15	high-density lipoprotein particle remodeling	5	3.97	2.88E-06	APOA4, APOM, APOA1, APOA2, APOC1	1.59E-04	0.0044
16	retinoid metabolic process	7	5.56	4.09E-06	APOB, RBP4, SDC4, APOA4, APOM, APOA1, APOA2	2.11E-04	0.0063
17	collagen catabolic process	7	5.56	5.42E-06	COL5A3, MMP8, PRTN3, MMP9, MMP7, FURIN, COL15A1	2.64E-04	0.0084
18	defense response to bacterium	9	7.14	7.86E-06	MPO, IGHG3, S100A9, IGHG4, IGHG1, ANXA3, HP, S100A8, IGHD	3.61E-04	0.0121
19	extracellular matrix organization	10	7.94	9.33E-06	FGG, COL5A3, BCAN, FGB, MATN4, DDR2, SPP1, LAMA4, VCAN, FURIN	4.07E-04	0.0144
20	high-density lipoprotein particle assembly	4	3.17	1.80E-05	APOA4, APOM, APOA1, APOA2	7.45E-04	0.0277

Table S8. KEGG pathway of enriched urine proteins in ketamine abusers of both genders generated using DAVID Bioinformatics resources

#	Term	Count	%	P Value	Genes	List Total	Pop Hits	Pop Total	Fold Enrichment	Bonferroni	Benjamini	FDR
1	hsa04610: Complement and coagulation cascades	20	15.9	1.00E-25	A2M, CPB2, F12, F13B, C1R, C2, C3, C5, C6, C8A, C9, C4BPA, CFB, CFH, FGB, FGG, KLKB1, PLG, SERPINC1, SERPIND1	55	69	6879	36.25	7.21E-24	7.21E-24	1.04E-22
2	hsa05150: Staphylococcus aureus infection	8	6.35	1.64E-07	C5, FGG, CFB, CFH,C3,PLG, C2, C1R	55	54	6879	18.53	1.18E-05	5.90E-06	1.71E-04

Table S9. List of fibrosis-related proteins among the differentially expressed urinary proteins of the KA group

No.	Accession no.	Gene name	Protein name	Ratio of 115/114 in male group [a]	Ratio of 117/116 in female group [b]	Disease [c]	Biomarker	Validated evidence (from Pubmed)
1	P27169	PON1	Serum paraoxonase/arylesterase	11.69	5.17	(chronic liver diseases)		" Furthermore, IL28B non-major genotype (rs8099917 TG/GG) was associated with low levels of serum apo B and high levels of apoA-II, and advanced fibrosis was associated with low levels of apo B and C-II in G1b infection." PMID:16510204(https://pubmed.ncbi.nlm.nih.gov/16510204/)Title:Paraoxonase-1 is associated with oxidative stress, fibrosis and FAS expression in chronic liver diseases
2	P02652	APOA2	Apolipoprotein A-II	11.13	3.68	(chronic HCV infection)		" Furthermore, IL28B non-major genotype (rs8099917 TG/GG) was associated with low levels of serum apo B and high levels of apoA-II, and advanced fibrosis was associated with low levels of apo B and C-II in G1b infection." PMID:26202760(https://pubmed.ncbi.nlm.nih.gov/26202760/)Title:Assessment of the features of serum apolipoprotein profiles in chronic HCV infection: difference between HCV genotypes 1b and 2
3	P02654	APOC1	Apolipoprotein C-I	10.02	3.76	Hepatic Fibrosis		" The 6646 Da marker protein for liver fibrosis was identified as apolipoprotein C-I." PMID:17171788(https://pubmed.ncbi.nlm.nih.gov/17171788/)Title:New multi protein patterns differentiate liver fibrosis stages and hepatocellular carcinoma in chronic hepatitis C serum samples
4	P00738	HP	Haptoglobin	6.96	6.23	Hepatic Fibrosis	" All the patients and the control group were submitted to full clinical history and examination, abdominal ultrasonography, CBC, liver biochemical profile and fibrosis biomarkers (apolipoprotein A1, haptoglobin,a2 marcoglobulin, GGT)." PMID: 24842131(https://pubmed.ncbi.nlm.nih.gov/24842131/) Title :Non-invasive fibrosis seromarkers as a predictor of liver fibrosis in chronic hepatitis C and/or non-alcoholic steatohepatitis	" These data demonstrate that haptoglobin and hemopexin together are essential for protection from splenomegaly and liver fibrosis resulting from intravascular hemolysis." PMID:12393471(https://pubmed.ncbi.nlm.nih.gov/12393471/)Title:Enhanced splenomegaly and severe liver inflammation in haptoglobin/hemopexin double-null mice after acute hemolysis
5	P02647	APOA1	Apolipoprotein A-I	6.23	3.36	Idiopathic Plumonary Fibrosis ;Pulmonary Fibrosis	" All the patients and the control group were submitted to full clinical history and examination, abdominal ultrasonography, CBC, liver biochemical profile and fibrosis biomarkers (apolipoprotein A1, haptoglobin, ,a2 marcoglobulin, GGT)." PMID: 24842131(https://pubmed.ncbi.nlm.nih.gov/24842131/) Title : Non-invasive fibrosis seromarkers as a predictor of liver fibrosis in chronic	" Role of lung apolipoprotein A-I in idiopathic pulmonary fibrosis: antiinflammatory and antifibrotic effect on experimental lung injury and fibrosis. " PMID:20463180(https://pubmed.ncbi.nlm.nih.gov/20463180/)Title:Role of lung apolipoprotein A-I in idiopathic pulmonary fibrosis: antiinflammatory and antifibrotic effect on experimental lung injury and fibrosis
6	P22894	MMP8	Neutrophil collagenase	6.14	5.60	(hypertension)		" In contrast, estrogen attenuated fibrosis by increasing MMP-8 concentrations and increasing collagen III fragments. " PMID:18420168(https://pubmed.ncbi.nlm.nih.gov/18420168/) Title:Estrogen effects on MMP-13 and MMP-14 regulation of left ventricular mass in Dahl salt-induced hypertension
7	P04114	APOB	Apolipoprotein B-100	5.74	3.55	(congenital hypocholesterolemia)		" Homozygous MTTP and APOB mutations may lead to hepatic steatosis and fibrosis despite metabolic differences in congenital hypocholesterolemia." PMID:24842304(https://pubmed.ncbi.nlm.nih.gov/24842304/)Title:Homozygous MTTP and APOB mutations may lead to hepatic steatosis and fibrosis despite metabolic differences in congenital hypocholesterolemia
8	P33241	LSP1	Lymphocyte-specific protein 1	5.52	2.32	(skin)		" These results demonstrate that the absence of LSP1 promotes fibrosis in the skin." PMID:18580965(18580965)Title:Increased severity of bleomycin-induced skin fibrosis in mice with leukocyte-specific protein 1 deficiency
9	P01023	A2M	Alpha-2-macroglobulin	5.41	4.08	Hepatic Fibrosis	"Novel biomarkers predict liver fibrosis in hepatitis C patients: alpha 2 macroglobulin, vitamin D binding protein and apolipoprotein AI." PMID: 20630109(https://pubmed.ncbi.nlm.nih.gov/20630109/)Title :Novel biomarkers predict liver fibrosis in hepatitis C patients: alpha 2 macroglobulin, vitamin D binding protein and apolipoprotein AI	" RESULTS: Hyaluronic acid, TIMP-1 andalpha2-macroglobulin were selected as having the best predictive accuracy for F2-F4 fibrosis (combined AUROC = 0.831)." PMID:15582126(https://pubmed.ncbi.nlm.nih.gov/15582126/)Title:Evaluation of a panel of non-invasive serum markers to differentiate mild from moderate-to-advanced liver fibrosis in chronic hepatitis C patients
10	P02679	FGG	Fibrinogen gamma chain	5.25	9.79	Hepatic Fibrosis		"RESULTS:Smurf2, FGG, PTAFR, CYP2D6, among others, increased in the fibrosis liver and a semi-quantitative RT-PCR confirmed the reliability of the cDNA microarray analysis." PMID:17054587(https://pubmed.ncbi.nlm.nih.gov/17054587/)Title:Abnormal expression of Smurf2 during the process of rat liver fibrosis
11	P05164	MPO	Myeloperoxidase	5.25	2.48	Cystic Fibrosis		"Levels of MPO were also increased in cystic fibrosis." PMID:8016773(https://pubmed.ncbi.nlm.nih.gov/8016773/)Title:Eosinophilic activation in cystic fibrosis
12	Q96IY4	CPB2	Carboxypeptidase B2	4.88	4.20	Pulmonary Fibrosis		" These results suggest that the anti-fibrinolytic activity of TAFI promotes lung fibrosis by hindering the rate at which fibrin is degraded. " PMID:16565485(https://pubmed.ncbi.nlm.nih.gov/16565485/) Title:Thrombin-activatable fibrinolysis inhibitor deficiency attenuates bleomycin-induced lung fibrosis
13	P01024	C3	Complement C3	4.58	5.33	(liver fibrosis)		" Western blot validation of all candidate markers using plasma samples from patients across all Ishak fibrosis scores showed that the markers which changed with increasing fibrosis most consistently included lipid transfer inhibitor protein, complement C3d, corticosteroid-binding globulin, apolipoprotein J and apolipoprotein L1." PMID:22761838(https://pubmed.ncbi.nlm.nih.gov/22761838/)Title:Discovery of novel biomarker candidates for liver fibrosis in hepatitis C patients: a preliminary study
14	P05160	F13B	Coagulation factor XIII B chain	4.52	3.03	Cystic Fibrosis		" Cystic fibrosis; hint of linkage with F13B." PMID:3856494(https://pubmed.ncbi.nlm.nih.gov/3856494/)Title:Cystic fibrosis; hint of linkage with F13B
15	P04196	HRG	Histidine-rich glycoprotein	4.44	3.93	Idiopathic Plumonary Fibrosis ;Pulmonary Fibrosis		" Because these processes are involved in the pathogeneses of lung fibrosis we here analyzed a possible link between HRG and idiopathic pulmonary fibrosis (IPF). " PMID:26525373(https://pubmed.ncbi.nlm.nih.gov/26525373/)Title:Histidine-rich glycoprotein and idiopathic pulmonary fibrosis
16	P00751	CFB	Complement factor B	3.94	2.99	(cardiac hypertrophy)		" In post-MI WT hearts expression of pro-inflammatory genes TNF-?? (Tnfa), complement factor B (Cfb), myocyte death and fibrosis were significantly increased, but increases were significantly less in MyD88(-/-) hearts after MI." PMID:22326848 (https://pubmed.ncbi.nlm.nih.gov/22326848/)Title:MyD88 mediated inflammatory signaling leads to CaMKII oxidation, cardiac hypertrophy and death after myocardial infarction
17	P02787	TF	Serotransferrin	3.65	2.54	(ferroportin disease)(liver fibrosis)	" Serum transferrin as a liver fibrosis biomarker in patients with chronic hepatitis B. BACKGROUND/AIMS:Transferrin and alpha-1 antitrypsin are reportedly associated with liver fibrosis. " PMID: 25548740(https://pubmed.ncbi.nlm.nih.gov/25548740/) Title :Serum transferrin as a liver fibrosis biomarker in patients with chronic hepatitis B	" Data indicated that transferrin saturation, which correlated with fibrosis and levels of alanine aminotransferase, might be a marker of disease severity. " PMID:21199650 (https://pubmed.ncbi.nlm.nih.gov/21199650/) Title: Sex and acquired cofactors determine phenotypes of ferroportin disease
18	P06681	C2	Complement C2	3.63	2.38	Cystic Fibrosis		" Serum complement C2 levels in patients suffering from cystic fibrosis (CF)." PMID:3269267 (https://pubmed.ncbi.nlm.nih.gov/3269267/) Title:Serum complement C2 levels in patients suffering from cystic fibrosis (CF)
19	P68871	HBB	Hemoglobin subunit beta	3.56	2.34	Cystic Fibrosis		" CONCLUSIONS: Heterozygosis forbeta-globin mutations is a novel risk factor for both hepatic iron accumulation and the progression to fibrosis in patients with CHC." PMID:17135308(https://pubmed.ncbi.nlm.nih.gov/17135308/)Title:Heterozygous beta-globin gene mutations as a risk factor for iron accumulation and liver fibrosis in chronic hepatitis C
20	P05109	S100A8	Protein S100-A8	3.56	2.15	Cystic Fibrosis		" S100A8 and S100A9 Are Induced by Decreased Hydration in the Epidermis and Promote Fibroblast Activation and Fibrosis in the Dermis." PMID:26597884(https://pubmed.ncbi.nlm.nih.gov/26597884/)Title:S100A8 and S100A9 Are Induced by Decreased Hydration in the Epidermis and Promote Fibroblast Activation and Fibrosis in the Dermis
21	Q14520	HABP2	Hyaluronan-binding protein 2	3.51	2.68	Hepatic Fibrosis		" A single nucleotide polymorphism (G534E, Marburg I, MI-SNP) in the gene encoding FSAP (HABP2) leads to lower enzymatic activity and is associated with enhanced liver fibrosis in humans." PMID:22989567(https://pubmed.ncbi.nlm.nih.gov/22989567/)Title:Factor VII activating protease (FSAP) exerts anti-inflammatory and anti-fibrotic effects in liver fibrosis in mice and men
22	P01031	C5	Complement C5	3.44	2.75			" Complement C5 mediates experimental tubulointerstitial fibrosis." PMID:17389734 (https://pubmed.ncbi.nlm.nih.gov/17389734/) Title:Complement C5 mediates experimental tubulointerstitial fibrosis
23	P02774	GC	Vitamin D-binding protein	3.32	2.16	Hepatic Fibrosis	" Novel biomarkers predict liver fibrosis in hepatitis C patients: alpha 2 macroglobulin, vitamin D binding protein and apolipoprotein AI. " PMID: 20630109(https://pubmed.ncbi.nlm.nih.gov/20630109/) Title: Novel biomarkers predict liver fibrosis in hepatitis C patients: alpha 2 macroglobulin, vitamin D binding protein and apolipoprotein AI	" Novel biomarkers predict liver fibrosis in hepatitis C patients: alpha 2 macroglobulin, vitamin D binding protein and apolipoprotein AI." PMID:20630109(https://pubmed.ncbi.nlm.nih.gov/20630109/)Title: Novel biomarkers predict liver fibrosis in hepatitis C patients: alpha 2 macroglobulin, vitamin D binding protein and apolipoprotein AI
24	P80188	LCN2	Neutrophil gelatinase-associated	3.09	2.61	Renal Fibrosis		" Accelerated renal fibrosis in cardiorenal syndrome is associated with long-term increase in urine neutrophil gelatinase-associated lipocalin levels." PMID:22889806(https://pubmed.ncbi.nlm.nih.gov/22889806/)Title:Accelerated renal fibrosis in cardiorenal syndrome is associated with long-term increase in urine neutrophil gelatinase-associated lipocalin levels
25	P36222	CHI3L1	Chitinase-3-like protein 1	3.08	2.82	Hepatic Fibrosis; Pulmonary Fibrosis	" Finally, we showed that CHI3L1 is superior to hyaluronic acid (HA), type III procollagen (PCIII), laminin (LN), and type IV collagen (CIV), which are also serum biomarkers of liver fibrosis, in identifying advanced liver fibrosis in patients with HBV-related liver fibrosis in China." PMID: 26415140 (https://pubmed.ncbi.nlm.nih.gov/26415140/) Title:CHI3L1 Is a Liver-Enriched, Noninvasive Biomarker That Can Be Used to Stage and Diagnose Substantial Hepatic Fibrosis	" These data demonstrate that CHI3L1-dependent pathways exacerbate pulmonary fibrosis and suggest CHI3L1 as a potential biomarker for pulmonary fibrosis progression and severity in HPS." PMID:26121745(https://pubmed.ncbi.nlm.nih.gov/26121745/)Title: Chitinase 3-like-1 and its receptors in Hermansky-Pudlak syndrome-associated lung disease
26	P01857	IGHG1	Immunoglobulin heavy constant	3.02	2.79	Cystic Fibrosis		" Whereas healthy adults and non-infected cystic fibrosis patients revealed mostly negative IgG subclass levels to the four antigens, infected cystic fibrosis patients had significantly elevated IgG1, IgG2, IgG3 and IgG4 levels to both the protein antigens as well as the polysaccharide antigen." PMID:2482060 (https://pubmed.ncbi.nlm.nih.gov/2482060/) Title:Increased levels of IgG subclasses specific for Pseudomonas aeruginosa exoenzyme and polysaccharide antigens in chronically infected
27	P06702	S100A9	Protein S100-A9	2.89	2.07	Idiopathic Pulmonary Fibrosis;Pulmonary Fibrosis;Renal Fibrosis	" S100A9 in BALF is a candidate biomarker of idiopathic pulmonary fibrosis." PMID:22209187(https://pubmed.ncbi.nlm.nih.gov/22209187/) Title:S100A9 in BALF is a candidate biomarker of idiopathic pulmonary fibrosis	" S100A9 in BALF is a candidate biomarker of idiopathic pulmonary fibrosis." PMID:22209187(https://pubmed.ncbi.nlm.nih.gov/22209187/)Title:S100A9 in BALF is a candidate biomarker of idiopathic pulmonary fibrosis
28	P24158	PRTN3	Myeloblastin	2.82	2.53	Cystic Fibrosis		" Proteinase 3 (PR3), a serine proteinase which can degrade lung tissue, is present in the cystic fibrosis (CF) sputum." PMID:10471824 (https://pubmed.ncbi.nlm.nih.gov/10471824/) Title:Proteinase 3 mRNA expression is induced in monocytes but not in neutrophils of patients with
29	P02768	ALB	Serum albumin	2.77	2.21	(chronic hepatitis B)		" RESULTS: Body mass index (BMI), platelet count,serum albumin, and total bilirubin levels were identified as independent predictors of bridging fibrosis or cirrhosis (Ishak stage 3-6)." PMID:15743360(https://pubmed.ncbi.nlm.nih.gov/15743360/)Title:Identification of chronic hepatitis B patients without significant liver fibrosis by a simple noninvasive predictive model
30	P26447	S100A4	Protein S100-A4	2.76	3.12	Hepatic Fibrosis		" S100A4 promotes liver fibrosis via activation of hepatic stellate cells." PMID:25111176(https://pubmed.ncbi.nlm.nih.gov/25111176/)Title:S100A4 promotes liver fibrosis via activation of hepatic stellate cells
31	P01860	IGHG3	Immunoglobulin heavy constant	2.72	4.67	Cystic Fibrosis;Hepatic Fibrosis		" These results imply that IgG3 against PM may not only provoke age-dependent resistance to S. japonicum infection but also enhance liver fibrosis." PMID:17297052 (https://pubmed.ncbi.nlm.nih.gov/17297052/) Title:Antibody isotype responses to paramyosin, a vaccine candidate for schistosomiasis, and their correlations with resistance and fibrosis in patients infected with Schistosoma japonicum in Leyte, The Philippines
32	P09237	MMP7	Matrilysin	2.60	3.10	Idiopathic Pulmonary Fibrosis;Pulmonary Fibrosis	" MMP1 and MMP7 as potential peripheral blood biomarkers in idiopathic pulmonary fibrosis." PMID: 18447576(https://pubmed.ncbi.nlm.nih.gov/18447576/) Title: MMP1 and MMP7 as potential peripheral blood biomarkers in idiopathic pulmonary fibrosis	" CONCLUSIONS: Hemodynamic overload of the atria is an important pathogenic factor of fibrosis; MMP-7 appears to be involved in the early stage of this tissue remodeling process." PMID:12875773(https://pubmed.ncbi.nlm.nih.gov/12875773/)Title:Fibrosis of the left atria during progression of heart failure is associated with increased matrix metalloproteinases in the rat
33	P14780	MMP9	Matrix metalloproteinase-9	2.53	2.46	Cardiac Fibrosis (Myocardial Fibrosis);Hepatic Fibrosis		" CONCLUSIONS: Intrahepatic COX-2, MMP-2, and MMP-9 overexpression is associated with progressive hepatic fibrosis in chronic HCV infection, suggesting their pathogenic role in fibrogenesis." PMID: 15479690 (https://pubmed.ncbi.nlm.nih.gov/15479690/)Title:ncreased intrahepatic cyclooxygenase 2, matrix metalloproteinase 2, and matrix metalloproteinase 9 expression is associated with progressive liver disease in chronic hepatitis C virus infection: role of viral core and NS5A proteins
34	P02763	ORM1	Alpha-1-acid glycoprotein 1	2.49	2.29	(liver diseases)		" The putative use of alpha-1-acid glycoprotein as a non-invasive marker of fibrosis." PMID:17004233(https://pubmed.ncbi.nlm.nih.gov/17004233/)Title:The putative use of alpha-1-acid glycoprotein as a non-invasive marker of fibrosis
35	Q15848	ADIPOQ	Adiponectin	2.27	2.04	Hepatic Fibrosis		"CONCLUSIONS: Levels ofadiponectin, reflecting PPAR-gamma activity, are correlated with skin fibrosis and might have potential utility as a biomarker in SSc. " PMID:22548780(https://pubmed.ncbi.nlm.nih.gov/22548780/)Title:Levels of adiponectin, a marker for PPAR-gamma activity, correlate with skin fibrosis in systemic sclerosis: potential utility as biomarker?
36	P02042	HBD	Hemoglobin subunit delta	2.23	2.46	Cystic Fibrosis		" Previous studies have implicated the novel peptide antibiotic human beta-defensin 1 (hBD-1) in the pathogenesis of cystic fibrosis. " PMID:9727055(https://pubmed.ncbi.nlm.nih.gov/9727055/) Title:Human beta-defensin 2 is a salt-sensitive peptide antibiotic expressed in human lung

37	P02790	HPX	Hemopexin	2.18	1.92	Hepatic Fibrosis	"Ten new **biomarkers** including protein disulfide isomerase A3, selenium-binding protein 1, glutamine synthetase, senescence marker protein 30, **hemopexin**, keratin 8, keratin 18, vimentin, Annexin A5 and dermatopontin associated with liver fibrosis were found and validated, and new insights through affecting multiple drug targets and biological processes were also provided to reveal the mechanisms of dioscin against **hepatic fibrosis** for the first time." PMID: 26069897 (https://pubmed.ncbi.nlm.nih.gov/26069897/) Title: Quantitative chemical proteomics for investigating the biomarkers of dioscin against liver fibrosis caused by CCl4 in rats	" These data demonstrate that **haptoglobin** and **hemopexin** together are essential for protection from splenomegaly and liver **fibrosis** resulting from intravascular hemolysis." **PMID**:12393471(https://pubmed.ncbi.nlm.nih.gov/12393471/)**Title**:Enhanced splenomegaly and severe liver inflammation in haptoglobin/hemopexin double-null mice after acute hemolysis
38	P01861	IGHG4	Immunoglobulin heavy constant	1.99	2.07	Cystic Fibrosis		"Whereas healthy adults and non-infected **cystic fibrosis** patients revealed mostly negative **IgG** subclass levels to the four antigens, infected **cystic fibrosis** patients had significantly elevated **IgG1**, **IgG**2, **IgG3** and **IgG4** levels to both the protein antigens as well as the polysaccharide antigen." **PMID**:2482060(https://pubmed.ncbi.nlm.nih.gov/2482060/)**Title**:Increased levels of IgG subclasses specific for Pseudomonas aeruginosa exoenzyme and polysaccharide antigens in chronically infected patients with cystic fibrosis
39	P02753	RBP4	Retinol-binding protein 4	1.98	1.84	(postoperative biliary atresia)	" **RBP4** may play a role in the pathogenesis of hepatic **fibrosis** and serve as a possible **biomarker** reflecting disease severity in postoperative BA patients." PMID: 21484122 (https://pubmed.ncbi.nlm.nih.gov/21484122/) Title :Serum retinol binding protein 4 and clinical outcome in postoperative biliary atresia	"Plasma **RBP4** levels tended to decrease concomitantly with the grade of histological **fibrosis**, activity, and steatosis." **PMID**:19302338(https://pubmed.ncbi.nlm.nih.gov/19302338/)**Title**:Patients achieving clearance of HCV with interferon therapy recover from decreased retinol-binding protein 4 levels
40	P80370	DLK1	Protein delta homolog 1	0.58	0.45	Hepatic Fibrosis		" Our findings provide a novel role of **Dlk1** in liver **fibrosis** leading to a better understanding of the molecular basis in **fibrosis** and cirrhosis and also give insights into the cellular and molecular mechanisms of MSC biology in liver repair. " **PMID**:21239501(https://pubmed.ncbi.nlm.nih.gov/21239501/)**Title**:Delta-like 1 serves as a new target and contributor to liver fibrosis down-regulated by mesenchymal stem cell transplantation
41	P13611	VCAN	Versican core protein	0.57	0.66	Acute Interstitial Pneumonia		" Glycoproteins (eg, thrombospondins, secreted protein acidic and rich in cysteine, tenascins), proteoglycans (eg, **versican**, syndecans, biglycan), and glycosaminoglycans (eg, hyaluronan, heparan sulfate) are upregulated on cardiac injury and regulate key processes in the remodeling myocardium such as inflammation, **fibrosis**, and angiogenesis. " **PMID**:24577967(https://pubmed.ncbi.nlm.nih.gov/24577967/)**Title**:Myocardial extracellular matrix: an ever-changing and diverse entity
42	P10451	SPP1	Osteopontin	0.53	0.60	Cardiac Fibrosis (Myocardial Fibrosis); Hepatic Fibrosis	" Serum **osteopontin** predicts degree of hepatic **fibrosis** and serves as a **biomarker** in patients with hepatitis C virus infection." PMID: 25760884(https://pubmed.ncbi.nlm.nih.gov/25760884/) Title:Serum osteopontin predicts degree of hepatic fibrosis and serves as a biomarker in patients with hepatitis C virus infection	**" Osteopontin** activates integrins that regulate cell adhesion, migration, and growth, thus implicating OPN in the process of cardiac **fibrosis**. " **PMID**:15120833(https://pubmed.ncbi.nlm.nih.gov/15120833/)**Title:Osteopontin modulates angiotensin II-induced fibrosis in the intact murine heart**
43	Q16832	DDR2	Discoidin domain-containing rec	0.53	0.48	Hepatic Fibrosis		" In conclusion, loss of **DDR2** promotes chronic liver **fibrosis** after CCl(4) injury." **PMID**:22019896 (https://pubmed.ncbi.nlm.nih.gov/22019896/) **Title**:Loss of discoidin domain receptor 2 promotes hepatic fibrosis after chronic carbon tetrachloride through altered paracrine interactions between hepatic stellate cells and liver-associated macrophages
44	P31431	SDC4	Syndecan-4	0.50	0.43	Pulmonary Fibrosis		" Collectively, these data suggest that the direct interaction of **syndecan-4** and CXCL10 in the lung interstitial compartment serves to inhibit fibroblast recruitment and subsequent **fibrosis**. " **PMID**:20484822 (https://pubmed.ncbi.nlm.nih.gov/20484822/)**Title**:nhibition of pulmonary fibrosis in mice by CXCL10 requires glycosaminoglycan binding and syndecan-4
45	P39059	COL15A1	Collagen alpha-1(XV) chain	0.47	0.53	(biliary or hepatocellular injury)		" **A** marked increase in **COL15A1** expression occurred together with that of the endothelial marker, von Willebrand factor, in human and rat liver tissue, at advanced stages of **fibrosis** caused by either biliary or hepatocellular injury. " **PMID**:25043701 (https://pubmed.ncbi.nlm.nih.gov/25043701/) **Title**:Portal myofibroblasts promote vascular remodeling underlying cirrhosis formation through the release of microparticles
46	Q16363	LAMA4	Laminin subunit alpha-4	0.42	0.43	(glomerular and tubulointerstitial fibrosis in Kidney)		" Although null mutations in the majority of laminin chains lead to specific developmental abnormalities in the kidney, **Lama4**-/- mice have progressive glomerular and tubulointerstitial **fibrosis**." **PMID**:20035058 (https://pubmed.ncbi.nlm.nih.gov/20035058/)**Title**:Laminin alpha4-null mutant mice develop chronic kidney disease with persistent overexpression of platelet-derived growth factor

a.115/114: the ratio of male ketamine abusers to male healthy controls

b.117/116: the ratio of female ketamine abusers to female healthy controls

c. All fibrosis-related proteins in urine samples of ketamine abusers were selected from FibroAtlas database 1.0 (http://biokb.ncpsb.org/fibroatlas/), which contained 1,439 manually curated fibrosis-related genes by literature mining.

Fig. S1

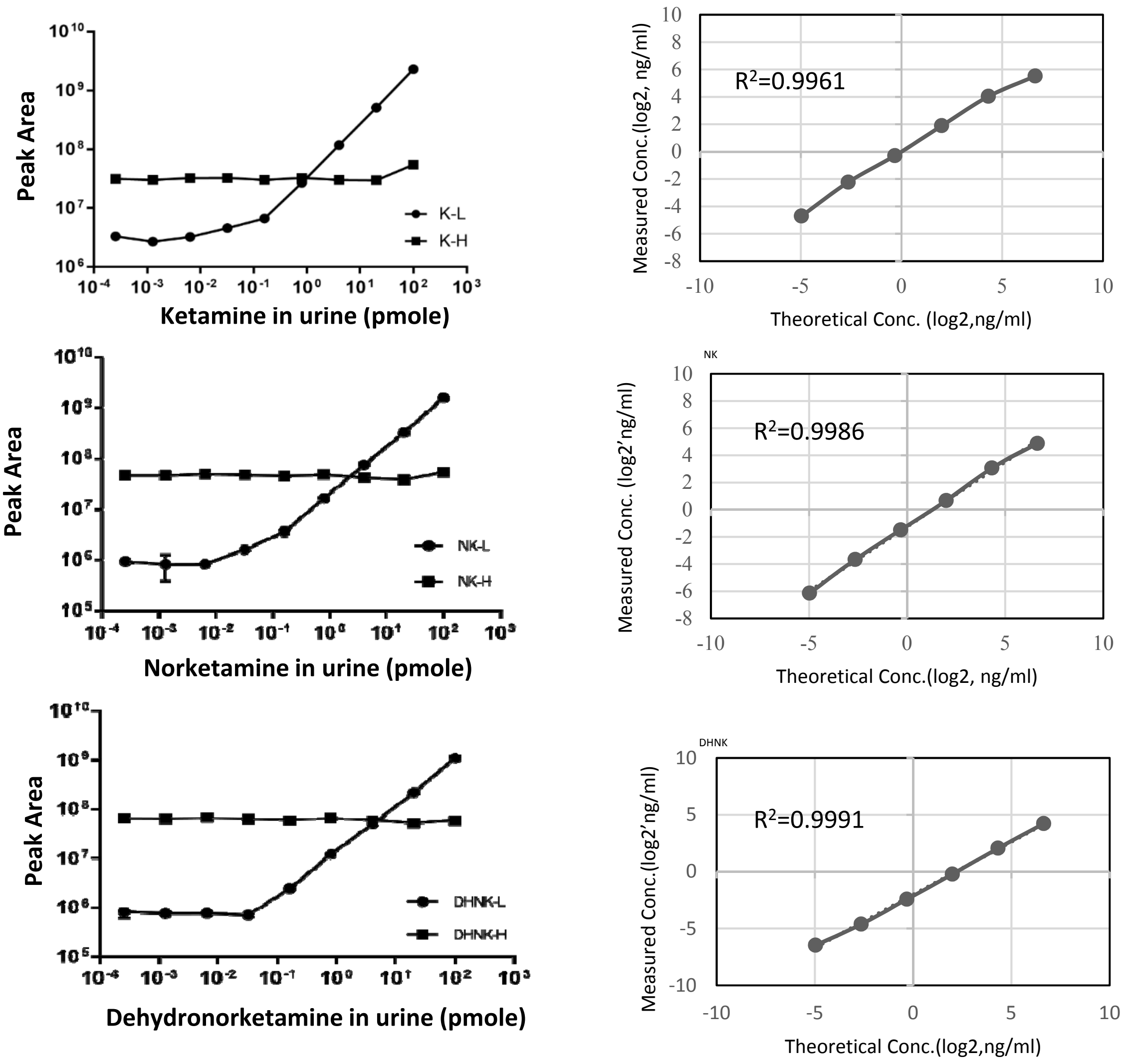

Figure S1. Response curves for quantification of ketamine, norketamine and dehydronorketamine generated via LC-SRM-MS analysis.

Fig. S2

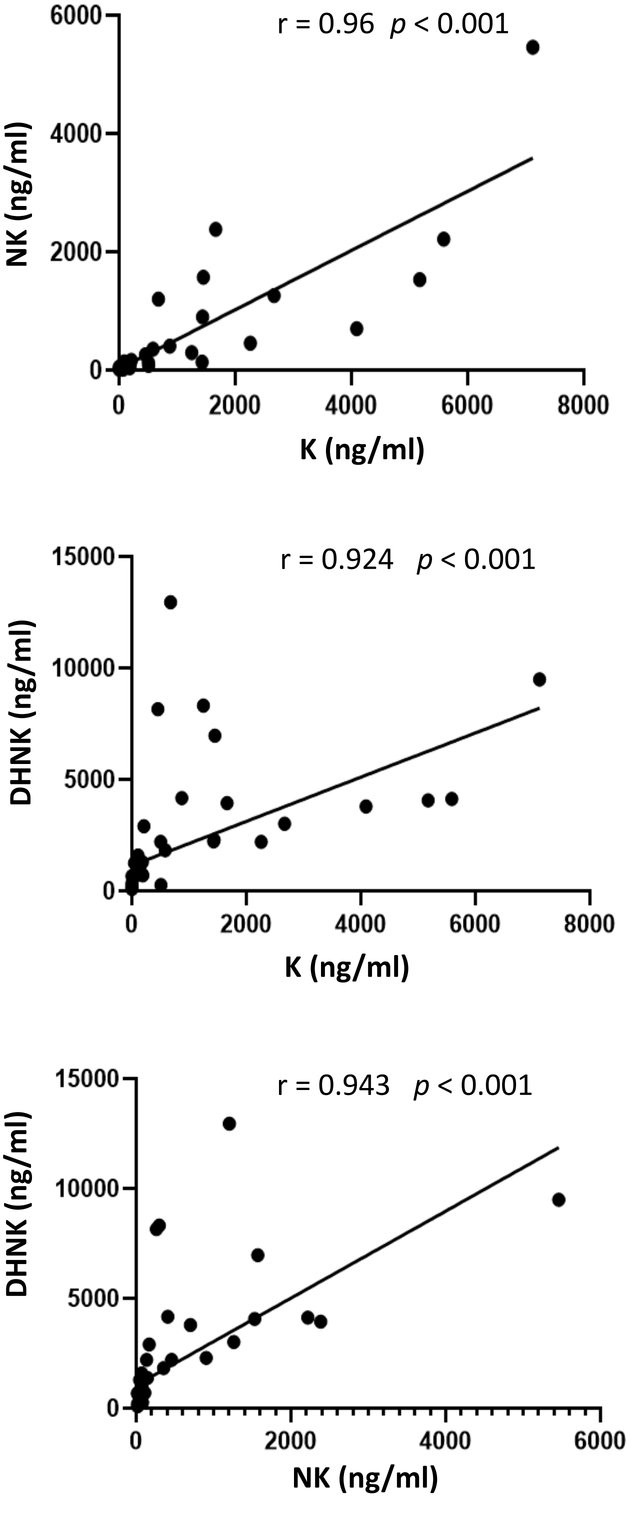

Figure S2. Correlations of urinary levels of ketamine (K), norketamine NK) and dehydronorketamine (DHNK) in ketamine abusers.

Fig. S3

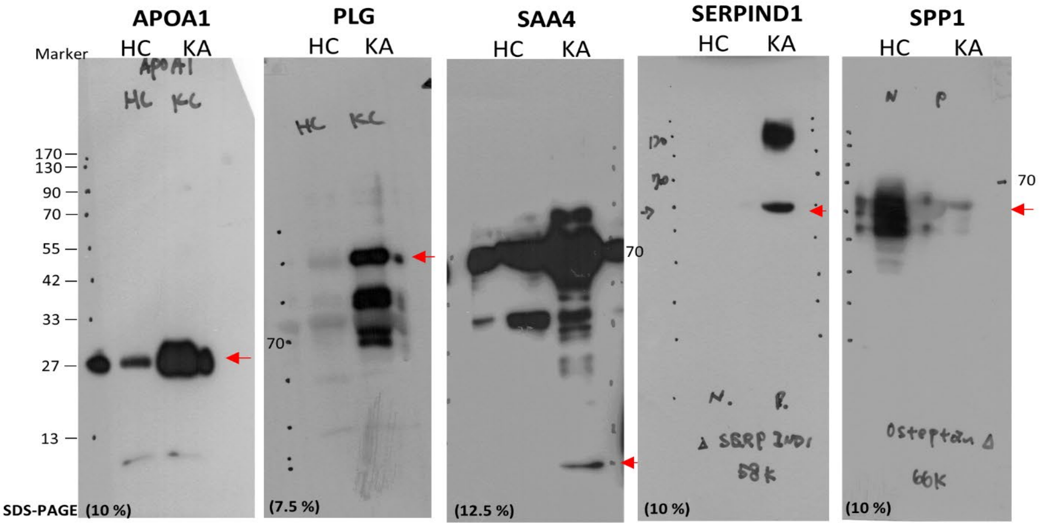

Figure S3. Detection of 5 candidate proteins in ketamine abuser's urine samples by Western blotting. Urine samples collected from health controls (HC) and ketamine abuser (KA) were analyzed by Western blotting using specific antibodies against APOA1 (30 KD), PLG (130 KD), SAA4 (12 KD), SERPIND1 (58 KD) or SPP1 (66 KD). Proteins (50 µg) from pooled urine samples used for the original iTRAQ experiment were subjected to Western blot analysis.
